# Supplementary material for: Nuclear genetic regulation of the human mitochondrial transcriptome
Source: eLife. 2019 Feb 18;8:e41927. doi: 10.7554/eLife.41927 (PMC6420317; doi:10.7554/eLife.41927)
Supplement: Supplementary file 6. — Each plot contains Beta estimates and confidence intervals for each of the datasets and tissues considered in the study. [file elife-41927-supp6.pdf]

Figure 2-figure supplement 1: Forest plots for each peak variant detected in association analyses. Each plot contains Beta estimates and confidence intervals for each of the datasets and tissues considered in the study.

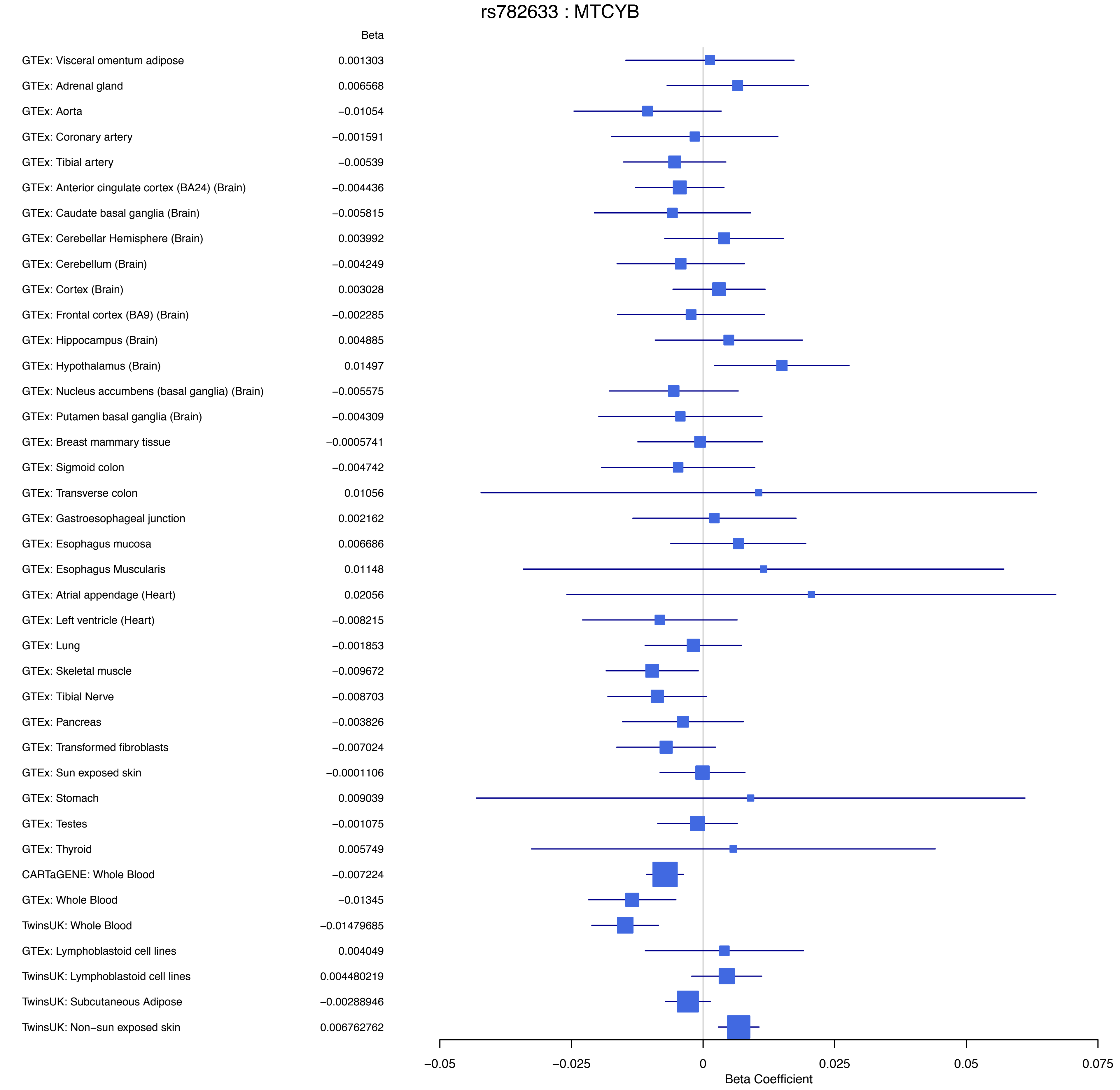
rs782633 : MTCYB
Beta
GTEx: Visceral omentum adipose 0.001303
GTEx: Adrenal gland 0.006568
GTEx: Aorta −0.01054
GTEx: Coronary artery −0.001591
GTEx: Tibial artery −0.00539
GTEx: Anterior cingulate cortex (BA24) (Brain) −0.004436
GTEx: Caudate basal ganglia (Brain) −0.005815
GTEx: Cerebellar Hemisphere (Brain) 0.003992
GTEx: Cerebellum (Brain) −0.004249
GTEx: Cortex (Brain) 0.003028
GTEx: Frontal cortex (BA9) (Brain) −0.002285
GTEx: Hippocampus (Brain) 0.004885
GTEx: Hypothalamus (Brain) 0.01497
GTEx: Nucleus accumbens (basal ganglia) (Brain) −0.005575
GTEx: Putamen basal ganglia (Brain) −0.004309
GTEx: Breast mammary tissue −0.0005741
GTEx: Sigmoid colon −0.004742
GTEx: Transverse colon 0.01056
GTEx: Gastroesophageal junction 0.002162
GTEx: Esophagus mucosa 0.006686
GTEx: Esophagus Muscularis 0.01148
GTEx: Atrial appendage (Heart) 0.02056
GTEx: Left ventricle (Heart) −0.008215
GTEx: Lung −0.001853
GTEx: Skeletal muscle −0.009672
GTEx: Tibial Nerve −0.008703
GTEx: Pancreas −0.003826
GTEx: Transformed fibroblasts −0.007024
GTEx: Sun exposed skin −0.0001106
GTEx: Stomach 0.009039
GTEx: Testes −0.001075
GTEx: Thyroid 0.005749
CARTaGENE: Whole Blood −0.007224
GTEx: Whole Blood −0.01345
TwinsUK: Whole Blood −0.01479685
GTEx: Lymphoblastoid cell lines 0.004049
TwinsUK: Lymphoblastoid cell lines 0.004480219
TwinsUK: Subcutaneous Adipose −0.00288946
TwinsUK: Non−sun exposed skin 0.006762762
−0.05
−0.025
0
0.025
0.05
0.075
Beta Coefficient

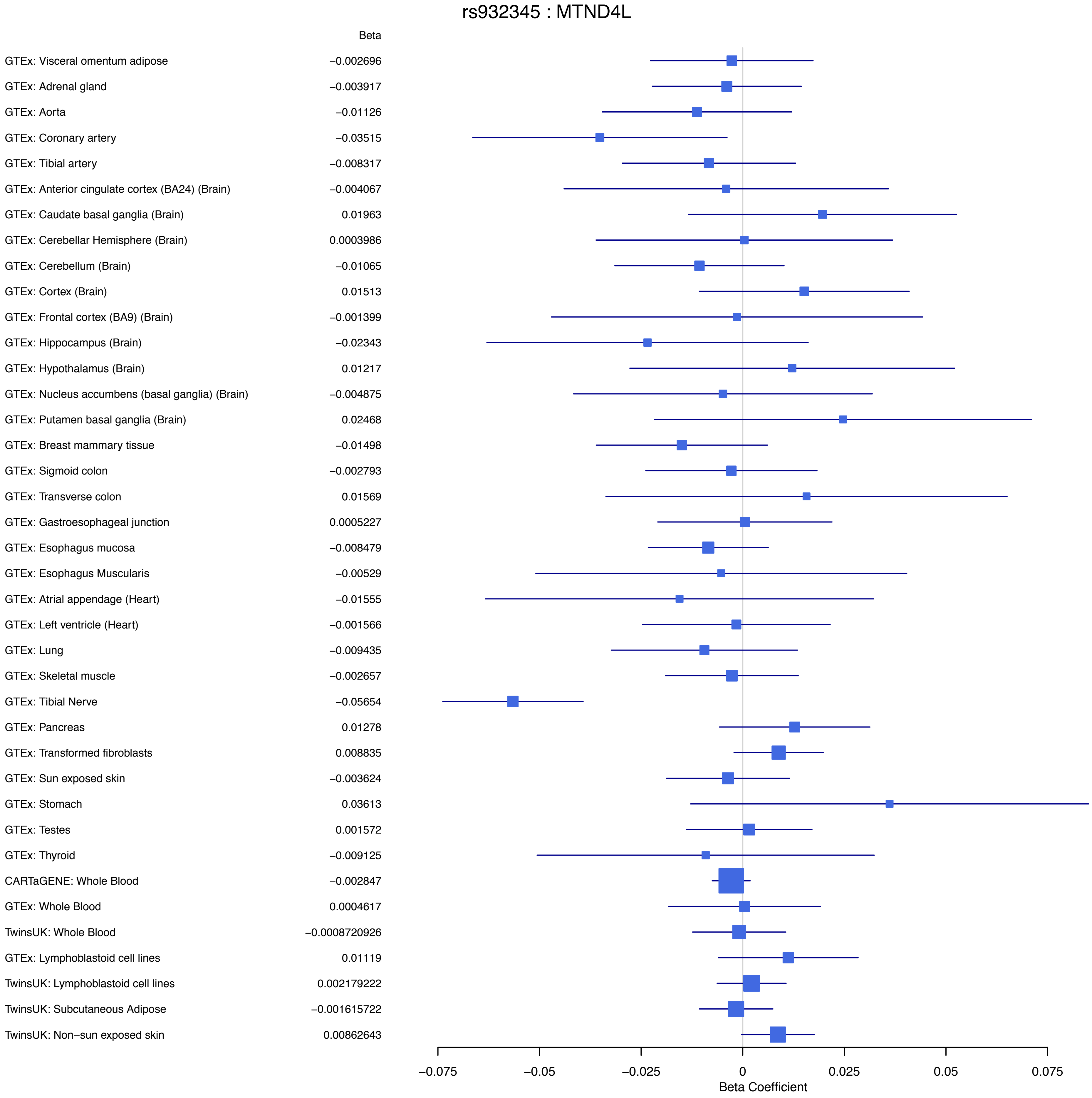

rs932345 : MTND4L
Beta
GTEx: Visceral omentum adipose −0.002696
GTEx: Adrenal gland −0.003917
GTEx: Aorta −0.01126
GTEx: Coronary artery −0.03515
GTEx: Tibial artery −0.008317
GTEx: Anterior cingulate cortex (BA24) (Brain) −0.004067
GTEx: Caudate basal ganglia (Brain) 0.01963
GTEx: Cerebellar Hemisphere (Brain) 0.0003986
GTEx: Cerebellum (Brain) −0.01065
GTEx: Cortex (Brain) 0.01513
GTEx: Frontal cortex (BA9) (Brain) −0.001399
GTEx: Hippocampus (Brain) −0.02343
GTEx: Hypothalamus (Brain) 0.01217
GTEx: Nucleus accumbens (basal ganglia) (Brain) −0.004875
GTEx: Putamen basal ganglia (Brain) 0.02468
GTEx: Breast mammary tissue −0.01498
GTEx: Sigmoid colon −0.002793
GTEx: Transverse colon 0.01569
GTEx: Gastroesophageal junction 0.0005227
GTEx: Esophagus mucosa −0.008479
GTEx: Esophagus Muscularis −0.00529
GTEx: Atrial appendage (Heart) −0.01555
GTEx: Left ventricle (Heart) −0.001566
GTEx: Lung −0.009435
GTEx: Skeletal muscle −0.002657
GTEx: Tibial Nerve −0.05654
GTEx: Pancreas 0.01278
GTEx: Transformed fibroblasts 0.008835
GTEx: Sun exposed skin −0.003624
GTEx: Stomach 0.03613
GTEx: Testes 0.001572
GTEx: Thyroid −0.009125
CARTaGENE: Whole Blood −0.002847
GTEx: Whole Blood 0.0004617
TwinsUK: Whole Blood −0.0008720926
GTEx: Lymphoblastoid cell lines 0.01119
TwinsUK: Lymphoblastoid cell lines 0.002179222
TwinsUK: Subcutaneous Adipose −0.001615722
TwinsUK: Non−sun exposed skin 0.00862643
−0.075
−0.05
−0.025
0
0.025
0.05
0.075
Beta Coefficient

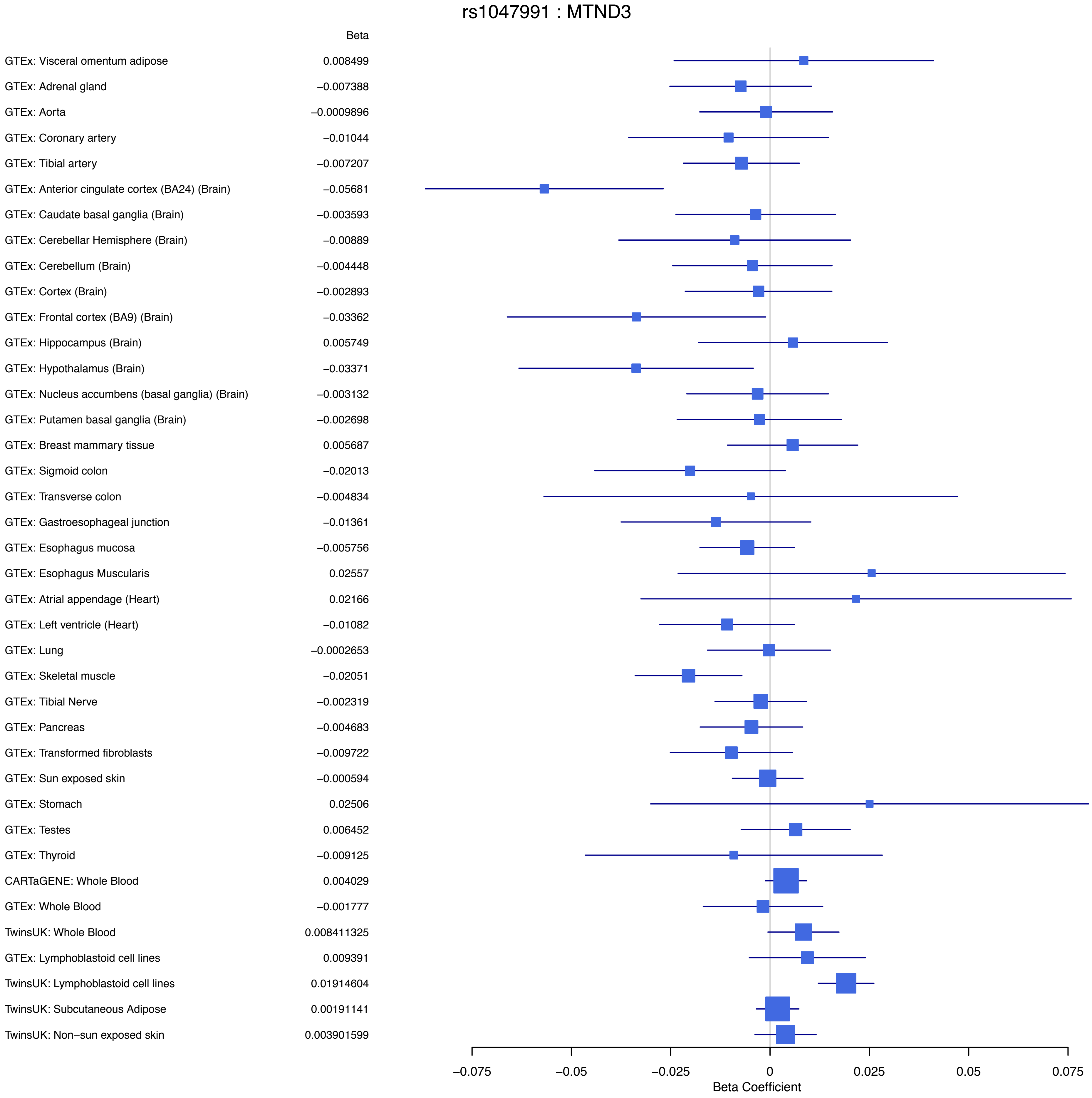
rs1047991 : MTND3
Beta
GTEx: Visceral omentum adipose 0.008499
GTEx: Adrenal gland −0.007388
GTEx: Aorta −0.0009896
GTEx: Coronary artery −0.01044
GTEx: Tibial artery −0.007207
GTEx: Anterior cingulate cortex (BA24) (Brain) −0.05681
GTEx: Caudate basal ganglia (Brain) −0.003593
GTEx: Cerebellar Hemisphere (Brain) −0.00889
GTEx: Cerebellum (Brain) −0.004448
GTEx: Cortex (Brain) −0.002893
GTEx: Frontal cortex (BA9) (Brain) −0.03362
GTEx: Hippocampus (Brain) 0.005749
GTEx: Hypothalamus (Brain) −0.03371
GTEx: Nucleus accumbens (basal ganglia) (Brain) −0.003132
GTEx: Putamen basal ganglia (Brain) −0.002698
GTEx: Breast mammary tissue 0.005687
GTEx: Sigmoid colon −0.02013
GTEx: Transverse colon −0.004834
GTEx: Gastroesophageal junction −0.01361
GTEx: Esophagus mucosa −0.005756
GTEx: Esophagus Muscularis 0.02557
GTEx: Atrial appendage (Heart) 0.02166
GTEx: Left ventricle (Heart) −0.01082
GTEx: Lung −0.0002653
GTEx: Skeletal muscle −0.02051
GTEx: Tibial Nerve −0.002319
GTEx: Pancreas −0.004683
GTEx: Transformed fibroblasts −0.009722
GTEx: Sun exposed skin −0.000594
GTEx: Stomach 0.02506
GTEx: Testes 0.006452
GTEx: Thyroid −0.009125
CARTaGENE: Whole Blood 0.004029
GTEx: Whole Blood −0.001777
TwinsUK: Whole Blood 0.008411325
GTEx: Lymphoblastoid cell lines 0.009391
TwinsUK: Lymphoblastoid cell lines 0.01914604
TwinsUK: Subcutaneous Adipose 0.00191141
TwinsUK: Non−sun exposed skin 0.003901599
−0.075
−0.05
−0.025
0
0.025
0.05
0.075
Beta Coefficient

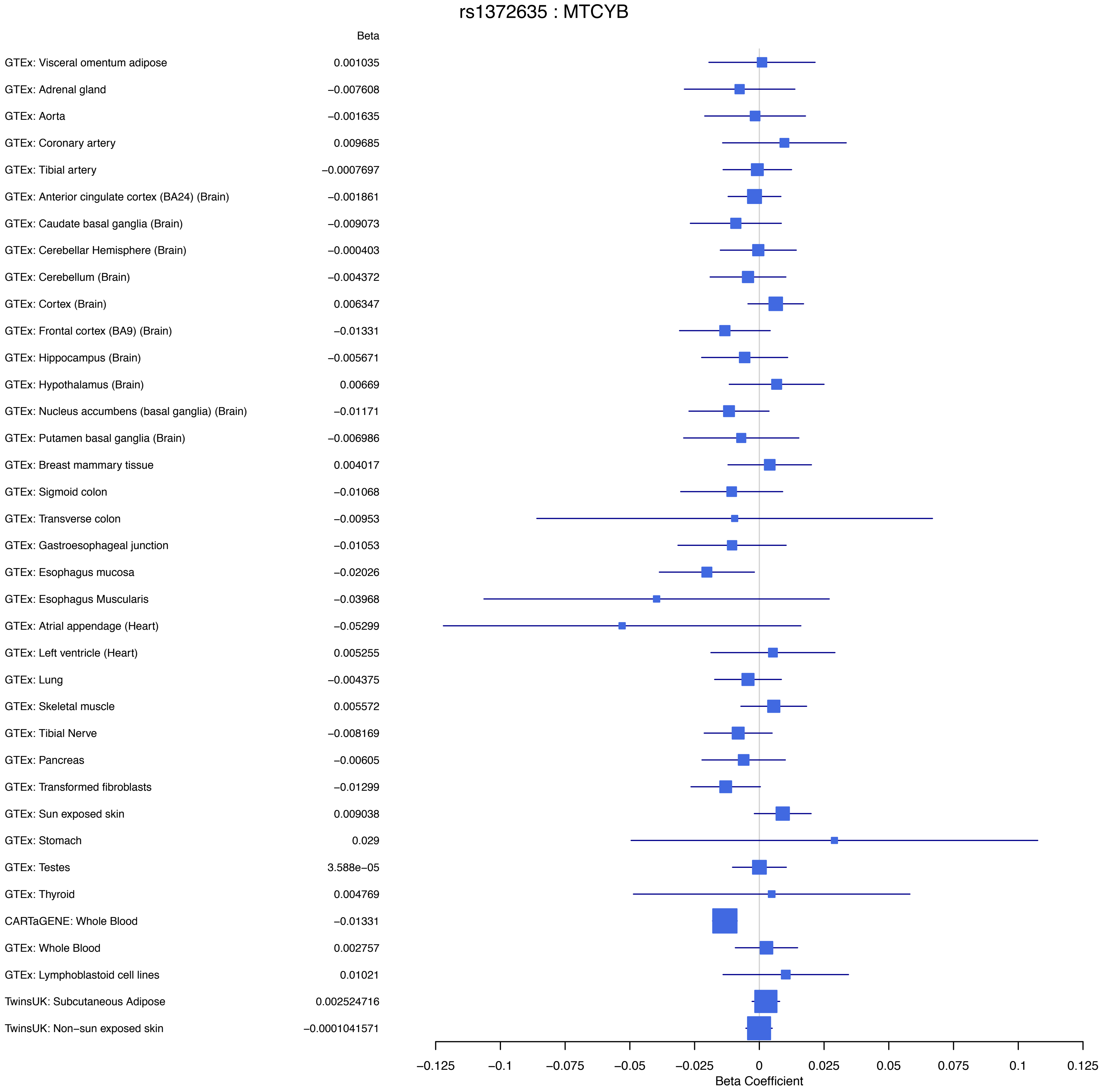
rs1372635 : MTCYB
Beta
GTEx: Visceral omentum adipose 0.001035
GTEx: Adrenal gland −0.007608
GTEx: Aorta −0.001635
GTEx: Coronary artery 0.009685
GTEx: Tibial artery −0.0007697
GTEx: Anterior cingulate cortex (BA24) (Brain) −0.001861
GTEx: Caudate basal ganglia (Brain) −0.009073
GTEx: Cerebellar Hemisphere (Brain) −0.000403
GTEx: Cerebellum (Brain) −0.004372
GTEx: Cortex (Brain) 0.006347
GTEx: Frontal cortex (BA9) (Brain) −0.01331
GTEx: Hippocampus (Brain) −0.005671
GTEx: Hypothalamus (Brain) 0.00669
GTEx: Nucleus accumbens (basal ganglia) (Brain) −0.01171
GTEx: Putamen basal ganglia (Brain) −0.006986
GTEx: Breast mammary tissue 0.004017
GTEx: Sigmoid colon −0.01068
GTEx: Transverse colon −0.00953
GTEx: Gastroesophageal junction −0.01053
GTEx: Esophagus mucosa −0.02026
GTEx: Esophagus Muscularis −0.03968
GTEx: Atrial appendage (Heart) −0.05299
GTEx: Left ventricle (Heart) 0.005255
GTEx: Lung −0.004375
GTEx: Skeletal muscle 0.005572
GTEx: Tibial Nerve −0.008169
GTEx: Pancreas −0.00605
GTEx: Transformed fibroblasts −0.01299
GTEx: Sun exposed skin 0.009038
GTEx: Stomach 0.029
GTEx: Testes 3.588e−05
GTEx: Thyroid 0.004769
CARTaGENE: Whole Blood −0.01331
GTEx: Whole Blood 0.002757
GTEx: Lymphoblastoid cell lines 0.01021
TwinsUK: Subcutaneous Adipose 0.002524716
TwinsUK: Non−sun exposed skin −0.0001041571
−0.125
−0.1
−0.075
−0.05
−0.025
0
0.025
0.05
0.075
0.1
0.125
Beta Coefficient

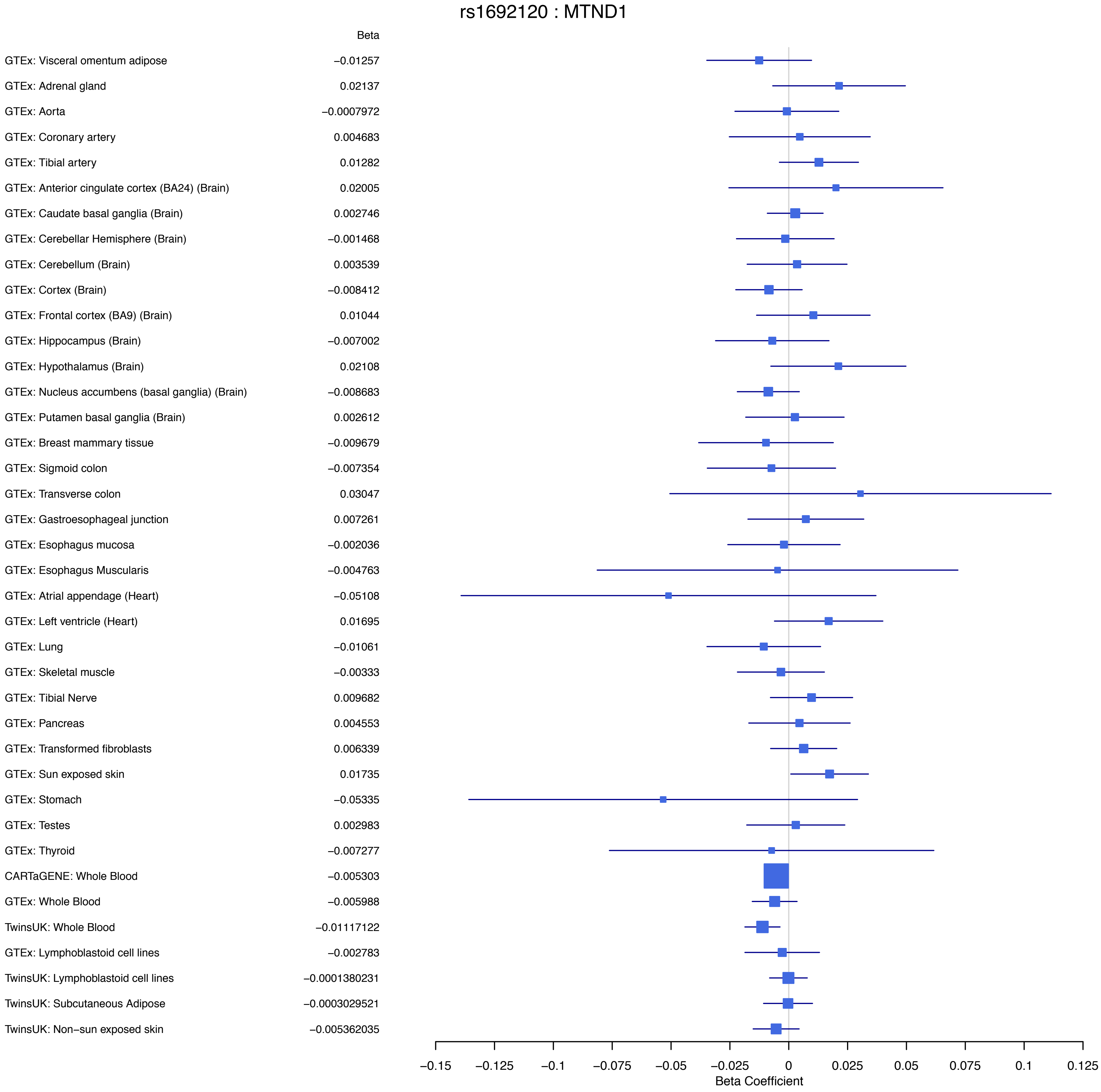
rs1692120 : MTND1
Beta
GTEx: Visceral omentum adipose −0.01257
GTEx: Adrenal gland 0.02137
GTEx: Aorta −0.0007972
GTEx: Coronary artery 0.004683
GTEx: Tibial artery 0.01282
GTEx: Anterior cingulate cortex (BA24) (Brain) 0.02005
GTEx: Caudate basal ganglia (Brain) 0.002746
GTEx: Cerebellar Hemisphere (Brain) −0.001468
GTEx: Cerebellum (Brain) 0.003539
GTEx: Cortex (Brain) −0.008412
GTEx: Frontal cortex (BA9) (Brain) 0.01044
GTEx: Hippocampus (Brain) −0.007002
GTEx: Hypothalamus (Brain) 0.02108
GTEx: Nucleus accumbens (basal ganglia) (Brain) −0.008683
GTEx: Putamen basal ganglia (Brain) 0.002612
GTEx: Breast mammary tissue −0.009679
GTEx: Sigmoid colon −0.007354
GTEx: Transverse colon 0.03047
GTEx: Gastroesophageal junction 0.007261
GTEx: Esophagus mucosa −0.002036
GTEx: Esophagus Muscularis −0.004763
GTEx: Atrial appendage (Heart) −0.05108
GTEx: Left ventricle (Heart) 0.01695
GTEx: Lung −0.01061
GTEx: Skeletal muscle −0.00333
GTEx: Tibial Nerve 0.009682
GTEx: Pancreas 0.004553
GTEx: Transformed fibroblasts 0.006339
GTEx: Sun exposed skin 0.01735
GTEx: Stomach −0.05335
GTEx: Testes 0.002983
GTEx: Thyroid −0.007277
CARTaGENE: Whole Blood −0.005303
GTEx: Whole Blood −0.005988
TwinsUK: Whole Blood −0.01117122
GTEx: Lymphoblastoid cell lines −0.002783
TwinsUK: Lymphoblastoid cell lines −0.0001380231
TwinsUK: Subcutaneous Adipose −0.0003029521
TwinsUK: Non−sun exposed skin −0.005362035
−0.15 −0.125 −0.1 −0.075 −0.05 −0.025 0 0.025 0.05 0.075 0.1 0.125
Beta Coefficient

rs2304693 : MTCO2

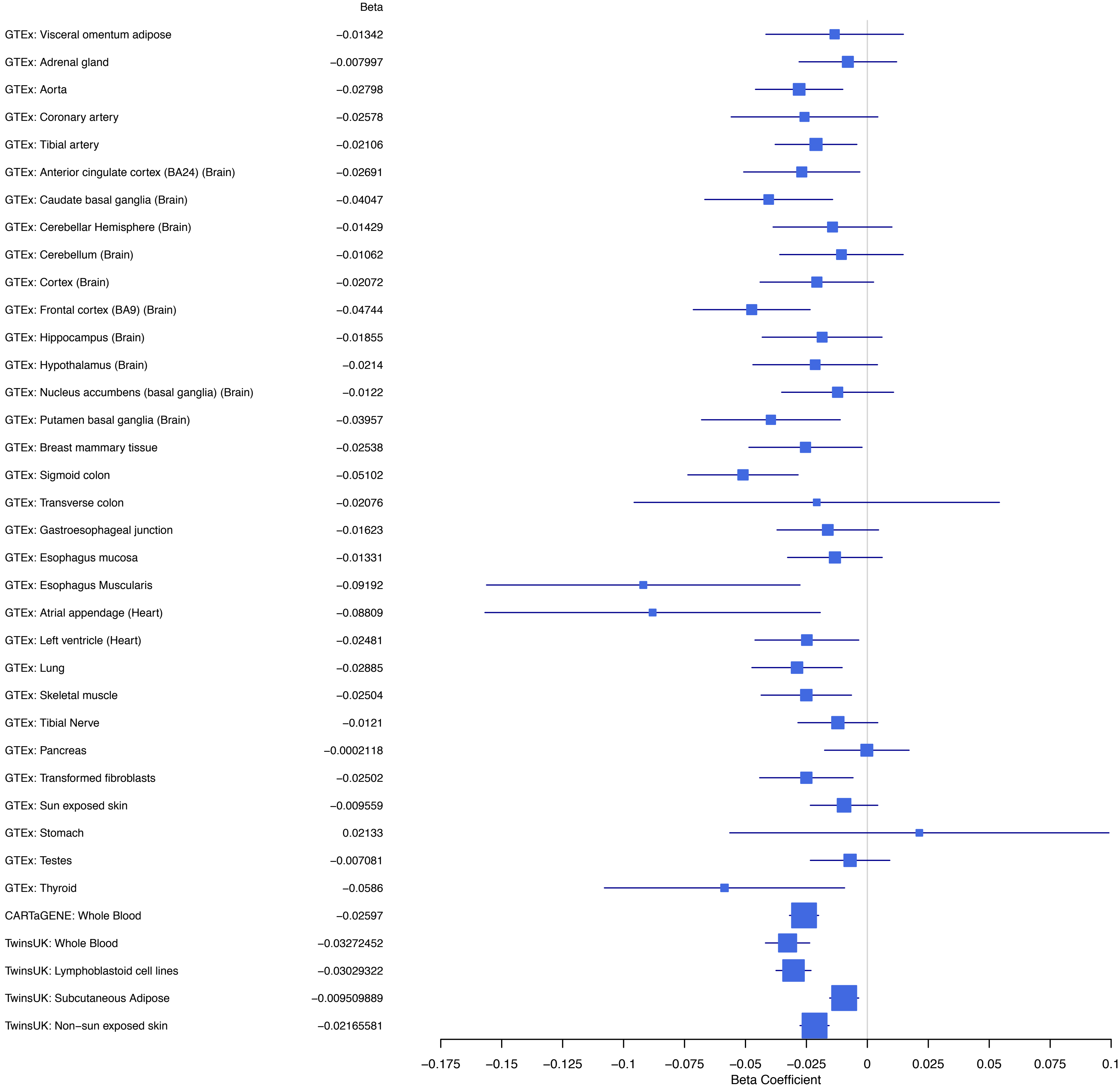

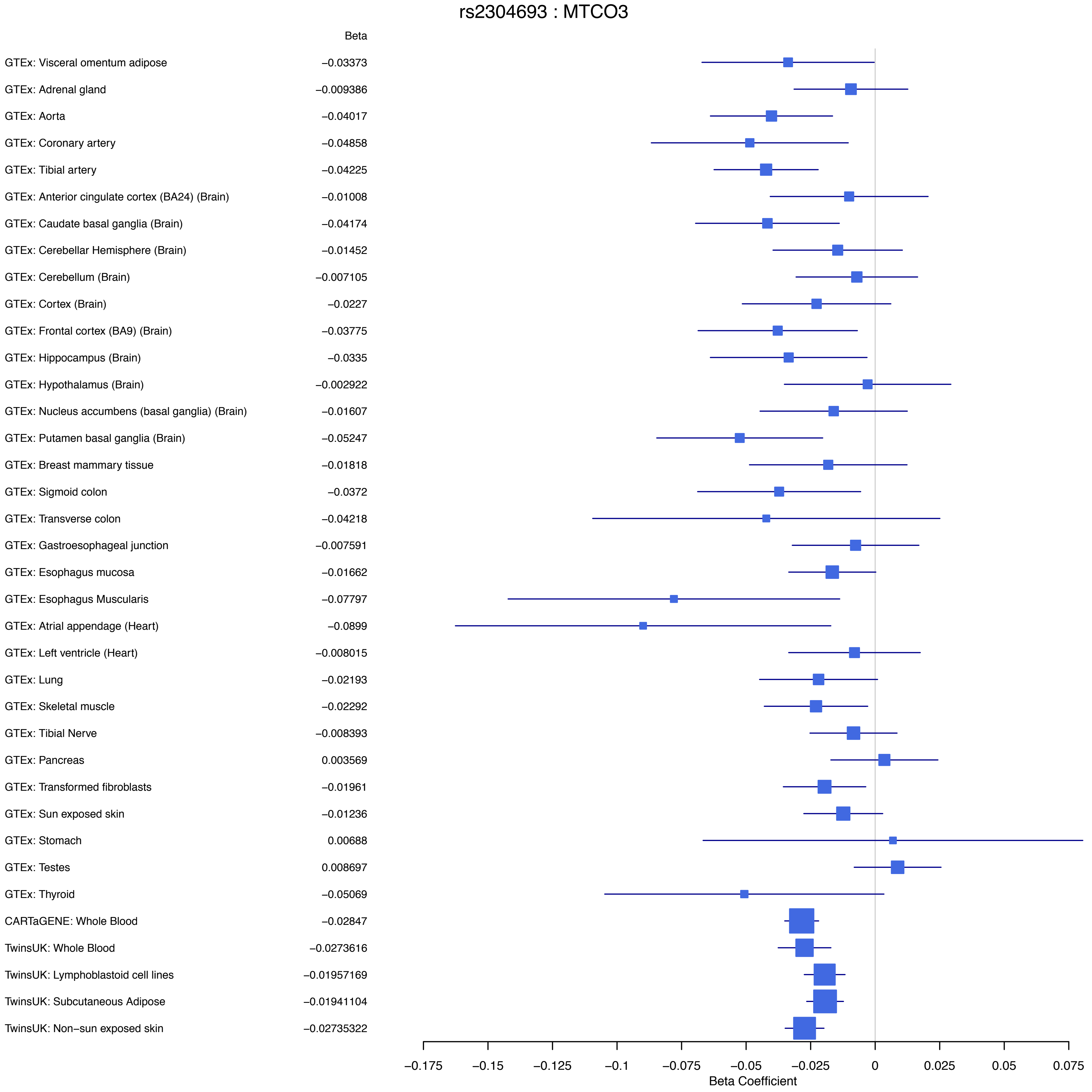
rs2304693 : MTCO3
Beta
GTEx: Visceral omentum adipose −0.03373
GTEx: Adrenal gland −0.009386
GTEx: Aorta −0.04017
GTEx: Coronary artery −0.04858
GTEx: Tibial artery −0.04225
GTEx: Anterior cingulate cortex (BA24) (Brain) −0.01008
GTEx: Caudate basal ganglia (Brain) −0.04174
GTEx: Cerebellar Hemisphere (Brain) −0.01452
GTEx: Cerebellum (Brain) −0.007105
GTEx: Cortex (Brain) −0.0227
GTEx: Frontal cortex (BA9) (Brain) −0.03775
GTEx: Hippocampus (Brain) −0.0335
GTEx: Hypothalamus (Brain) −0.002922
GTEx: Nucleus accumbens (basal ganglia) (Brain) −0.01607
GTEx: Putamen basal ganglia (Brain) −0.05247
GTEx: Breast mammary tissue −0.01818
GTEx: Sigmoid colon −0.0372
GTEx: Transverse colon −0.04218
GTEx: Gastroesophageal junction −0.007591
GTEx: Esophagus mucosa −0.01662
GTEx: Esophagus Muscularis −0.07797
GTEx: Atrial appendage (Heart) −0.0899
GTEx: Left ventricle (Heart) −0.008015
GTEx: Lung −0.02193
GTEx: Skeletal muscle −0.02292
GTEx: Tibial Nerve −0.008393
GTEx: Pancreas 0.003569
GTEx: Transformed fibroblasts −0.01961
GTEx: Sun exposed skin −0.01236
GTEx: Stomach 0.00688
GTEx: Testes 0.008697
GTEx: Thyroid −0.05069
CARTaGENE: Whole Blood −0.02847
TwinsUK: Whole Blood −0.0273616
TwinsUK: Lymphoblastoid cell lines −0.01957169
TwinsUK: Subcutaneous Adipose −0.01941104
TwinsUK: Non−sun exposed skin −0.02735322
−0.175 −0.15 −0.125 −0.1 −0.075 −0.05 −0.025 0 0.025 0.05 0.075
Beta Coefficient

rs2304693 : MTCYB

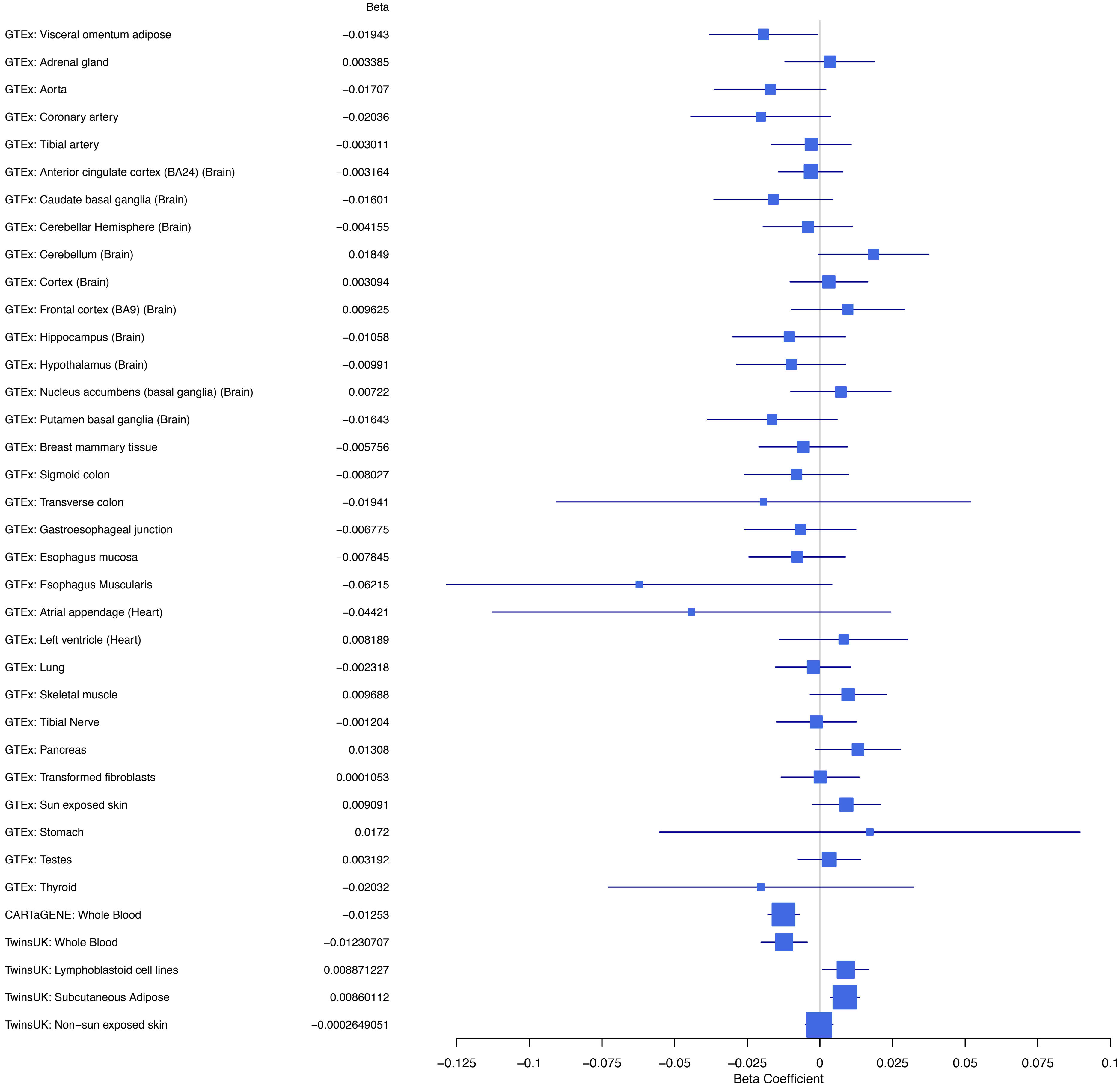

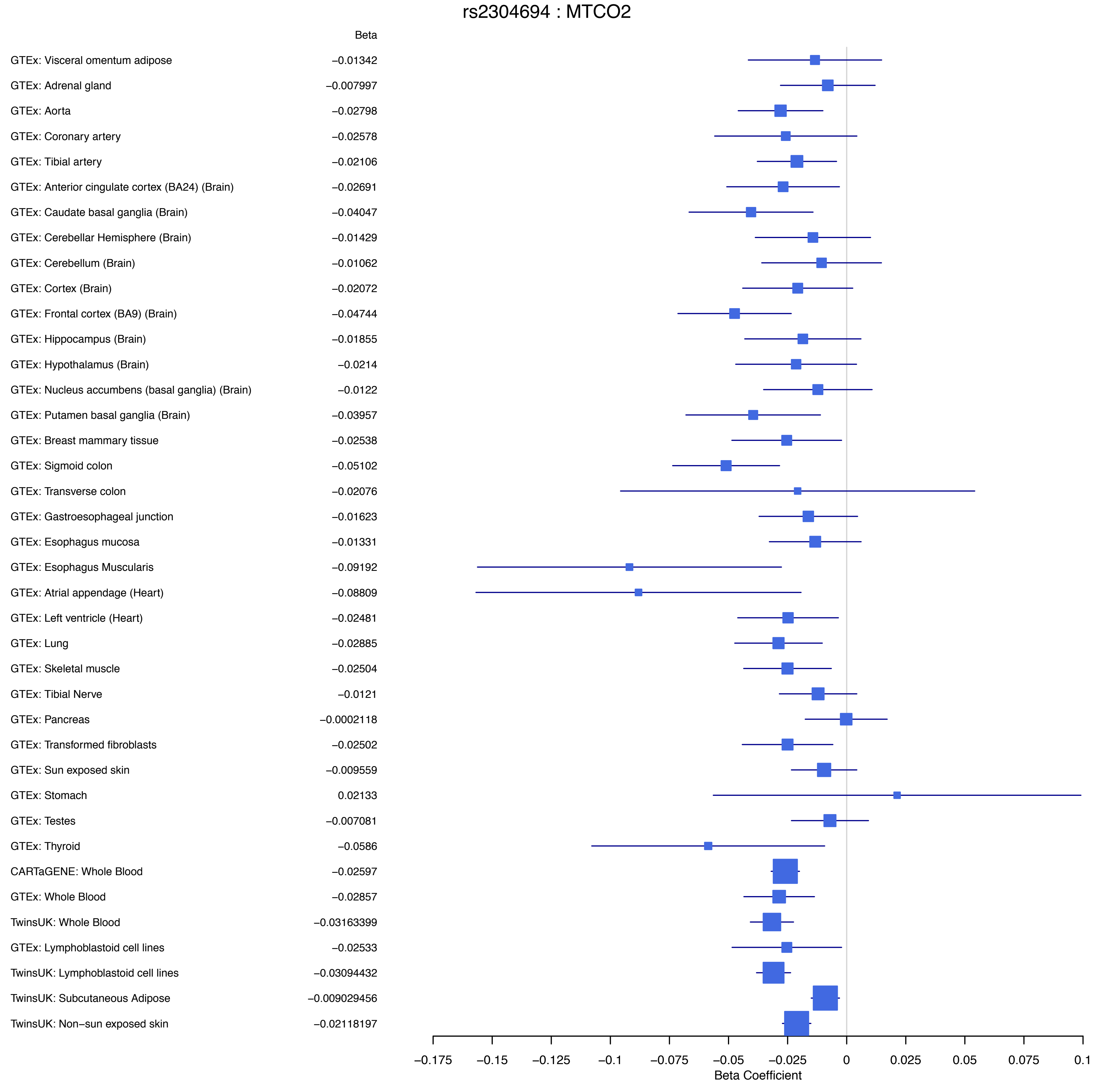
rs2304694 : MTCO2
Beta
GTEx: Visceral omentum adipose −0.01342
GTEx: Adrenal gland −0.007997
GTEx: Aorta −0.02798
GTEx: Coronary artery −0.02578
GTEx: Tibial artery −0.02106
GTEx: Anterior cingulate cortex (BA24) (Brain) −0.02691
GTEx: Caudate basal ganglia (Brain) −0.04047
GTEx: Cerebellar Hemisphere (Brain) −0.01429
GTEx: Cerebellum (Brain) −0.01062
GTEx: Cortex (Brain) −0.02072
GTEx: Frontal cortex (BA9) (Brain) −0.04744
GTEx: Hippocampus (Brain) −0.01855
GTEx: Hypothalamus (Brain) −0.0214
GTEx: Nucleus accumbens (basal ganglia) (Brain) −0.0122
GTEx: Putamen basal ganglia (Brain) −0.03957
GTEx: Breast mammary tissue −0.02538
GTEx: Sigmoid colon −0.05102
GTEx: Transverse colon −0.02076
GTEx: Gastroesophageal junction −0.01623
GTEx: Esophagus mucosa −0.01331
GTEx: Esophagus Muscularis −0.09192
GTEx: Atrial appendage (Heart) −0.08809
GTEx: Left ventricle (Heart) −0.02481
GTEx: Lung −0.02885
GTEx: Skeletal muscle −0.02504
GTEx: Tibial Nerve −0.0121
GTEx: Pancreas −0.0002118
GTEx: Transformed fibroblasts −0.02502
GTEx: Sun exposed skin −0.009559
GTEx: Stomach 0.02133
GTEx: Testes −0.007081
GTEx: Thyroid −0.0586
CARTaGENE: Whole Blood −0.02597
GTEx: Whole Blood −0.02857
TwinsUK: Whole Blood −0.03163399
GTEx: Lymphoblastoid cell lines −0.02533
TwinsUK: Lymphoblastoid cell lines −0.03094432
TwinsUK: Subcutaneous Adipose −0.009029456
TwinsUK: Non−sun exposed skin −0.02118197
−0.175 −0.15 −0.125 −0.1 −0.075 −0.05 −0.025 0 0.025 0.05 0.075 0.1
Beta Coefficient

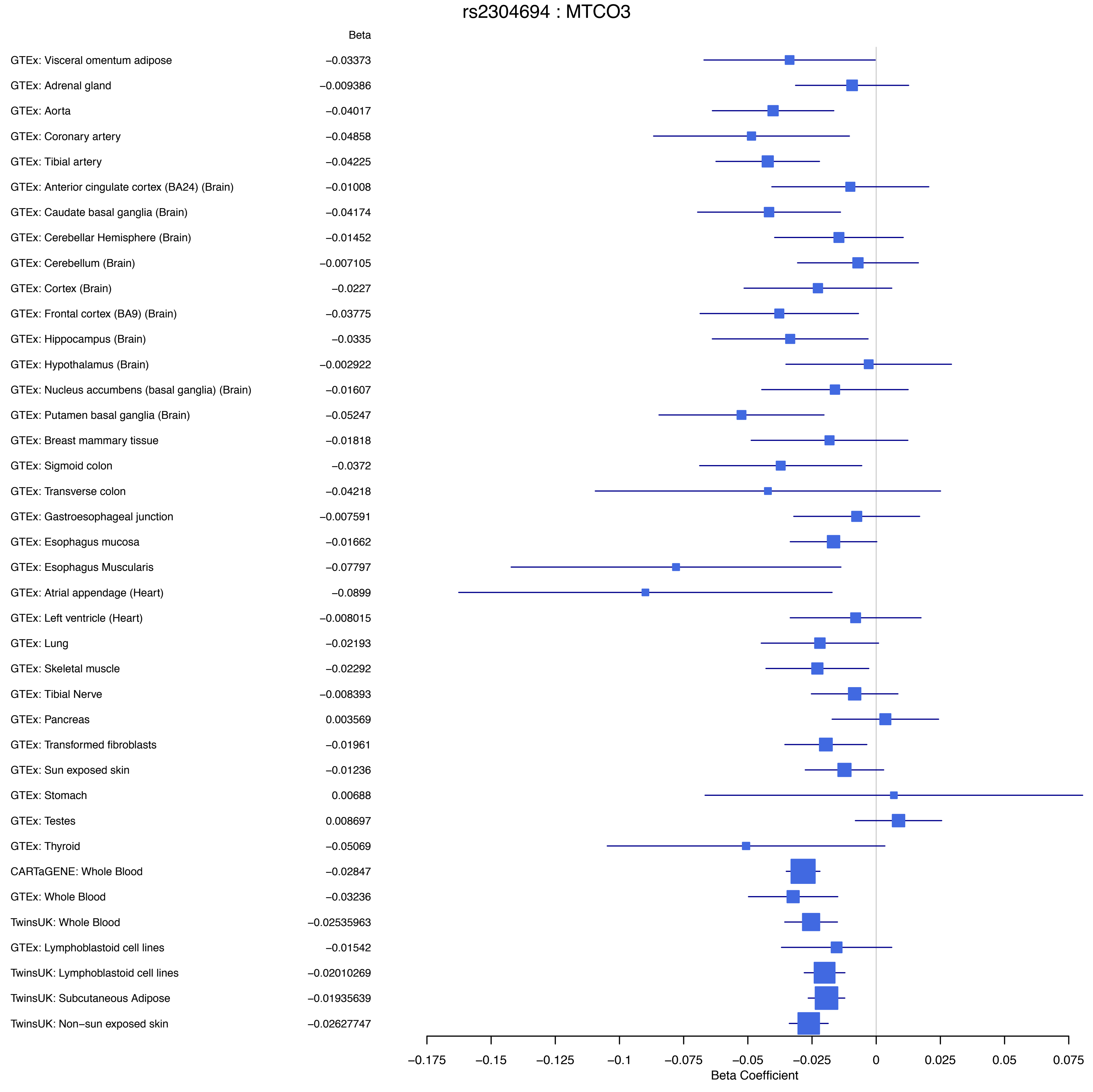
rs2304694 : MTCO3
Beta
GTEx: Visceral omentum adipose
−0.03373
GTEx: Adrenal gland
−0.009386
GTEx: Aorta
−0.04017
GTEx: Coronary artery
−0.04858
GTEx: Tibial artery
−0.04225
GTEx: Anterior cingulate cortex (BA24) (Brain)
−0.01008
GTEx: Caudate basal ganglia (Brain)
−0.04174
GTEx: Cerebellar Hemisphere (Brain)
−0.01452
GTEx: Cerebellum (Brain)
−0.007105
GTEx: Cortex (Brain)
−0.0227
GTEx: Frontal cortex (BA9) (Brain)
−0.03775
GTEx: Hippocampus (Brain)
−0.0335
GTEx: Hypothalamus (Brain)
−0.002922
GTEx: Nucleus accumbens (basal ganglia) (Brain)
−0.01607
GTEx: Putamen basal ganglia (Brain)
−0.05247
GTEx: Breast mammary tissue
−0.01818
GTEx: Sigmoid colon
−0.0372
GTEx: Transverse colon
−0.04218
GTEx: Gastroesophageal junction
−0.007591
GTEx: Esophagus mucosa
−0.01662
GTEx: Esophagus Muscularis
−0.07797
GTEx: Atrial appendage (Heart)
−0.0899
GTEx: Left ventricle (Heart)
−0.008015
GTEx: Lung
−0.02193
GTEx: Skeletal muscle
−0.02292
GTEx: Tibial Nerve
−0.008393
GTEx: Pancreas
0.003569
GTEx: Transformed fibroblasts
−0.01961
GTEx: Sun exposed skin
−0.01236
GTEx: Stomach
0.00688
GTEx: Testes
0.008697
GTEx: Thyroid
−0.05069
CARTaGENE: Whole Blood
−0.02847
GTEx: Whole Blood
−0.03236
TwinsUK: Whole Blood
−0.02535963
GTEx: Lymphoblastoid cell lines
−0.01542
TwinsUK: Lymphoblastoid cell lines
−0.02010269
TwinsUK: Subcutaneous Adipose
−0.01935639
TwinsUK: Non−sun exposed skin
−0.02627747
−0.175
−0.15
−0.125
−0.1
−0.075
−0.05
−0.025
0
0.025
0.05
0.075
Beta Coefficient

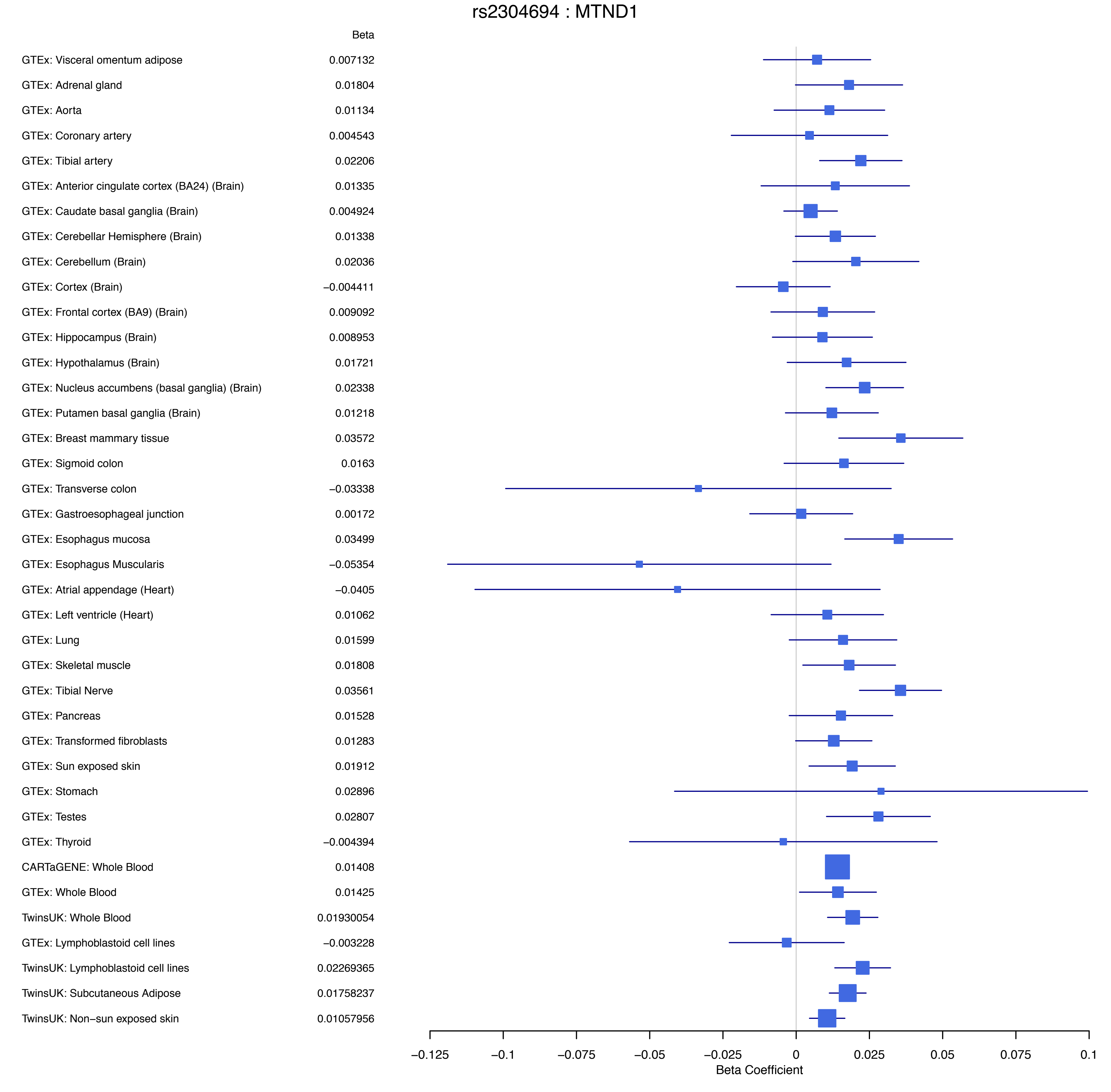
rs2304694 : MTND1
Beta
GTEx: Visceral omentum adipose 0.007132
GTEx: Adrenal gland 0.01804
GTEx: Aorta 0.01134
GTEx: Coronary artery 0.004543
GTEx: Tibial artery 0.02206
GTEx: Anterior cingulate cortex (BA24) (Brain) 0.01335
GTEx: Caudate basal ganglia (Brain) 0.004924
GTEx: Cerebellar Hemisphere (Brain) 0.01338
GTEx: Cerebellum (Brain) 0.02036
GTEx: Cortex (Brain) −0.004411
GTEx: Frontal cortex (BA9) (Brain) 0.009092
GTEx: Hippocampus (Brain) 0.008953
GTEx: Hypothalamus (Brain) 0.01721
GTEx: Nucleus accumbens (basal ganglia) (Brain) 0.02338
GTEx: Putamen basal ganglia (Brain) 0.01218
GTEx: Breast mammary tissue 0.03572
GTEx: Sigmoid colon 0.0163
GTEx: Transverse colon −0.03338
GTEx: Gastroesophageal junction 0.00172
GTEx: Esophagus mucosa 0.03499
GTEx: Esophagus Muscularis −0.05354
GTEx: Atrial appendage (Heart) −0.0405
GTEx: Left ventricle (Heart) 0.01062
GTEx: Lung 0.01599
GTEx: Skeletal muscle 0.01808
GTEx: Tibial Nerve 0.03561
GTEx: Pancreas 0.01528
GTEx: Transformed fibroblasts 0.01283
GTEx: Sun exposed skin 0.01912
GTEx: Stomach 0.02896
GTEx: Testes 0.02807
GTEx: Thyroid −0.004394
CARTaGENE: Whole Blood 0.01408
GTEx: Whole Blood 0.01425
TwinsUK: Whole Blood 0.01930054
GTEx: Lymphoblastoid cell lines −0.003228
TwinsUK: Lymphoblastoid cell lines 0.02269365
TwinsUK: Subcutaneous Adipose 0.01758237
TwinsUK: Non−sun exposed skin 0.01057956
−0.125
−0.1
−0.075
−0.05
−0.025
0
0.025
0.05
0.075
0.1
Beta Coefficient

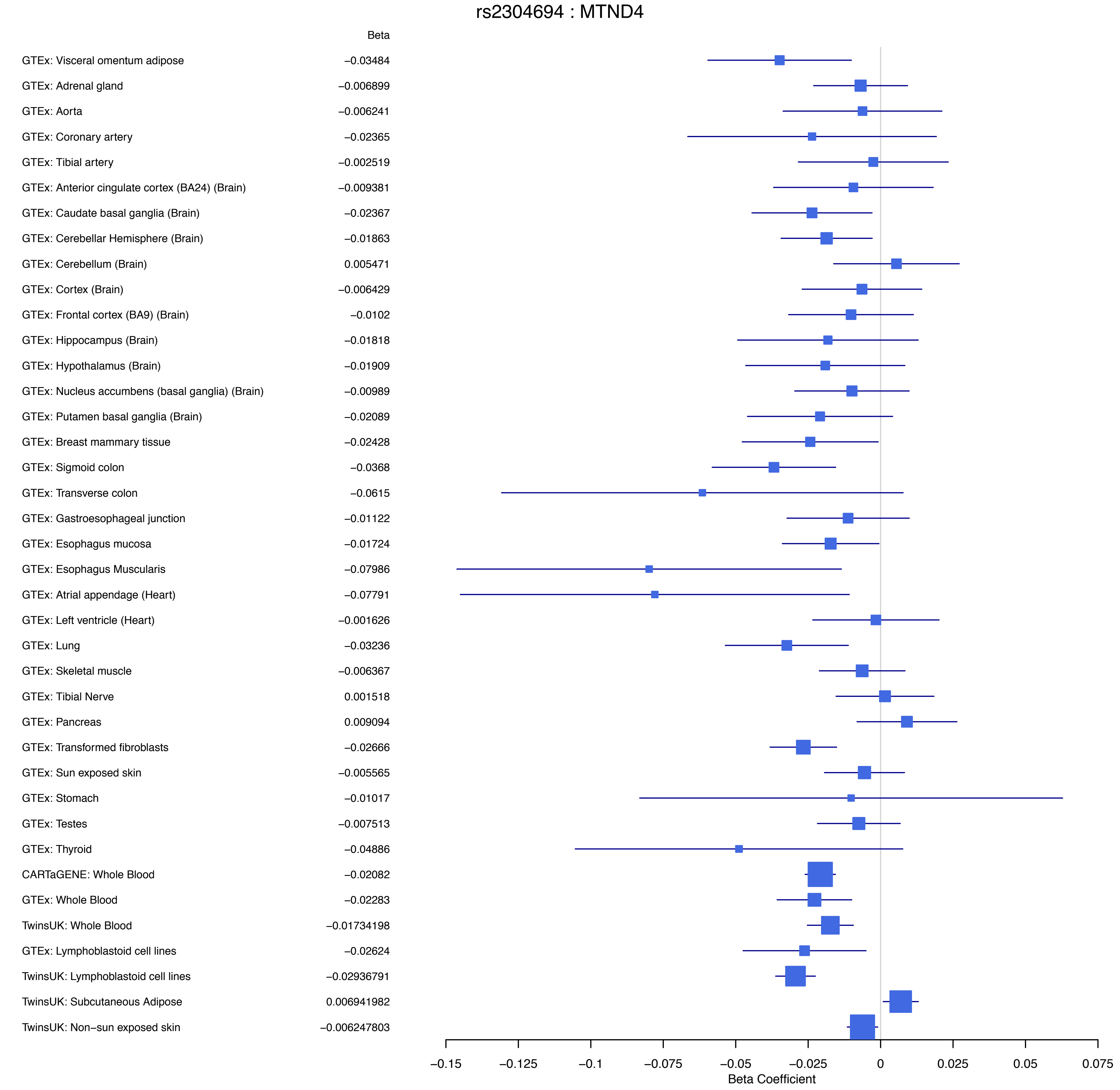
rs2304694 : MTND4
Beta
GTEx: Visceral omentum adipose −0.03484
GTEx: Adrenal gland −0.006899
GTEx: Aorta −0.006241
GTEx: Coronary artery −0.02365
GTEx: Tibial artery −0.002519
GTEx: Anterior cingulate cortex (BA24) (Brain) −0.009381
GTEx: Caudate basal ganglia (Brain) −0.02367
GTEx: Cerebellar Hemisphere (Brain) −0.01863
GTEx: Cerebellum (Brain) 0.005471
GTEx: Cortex (Brain) −0.006429
GTEx: Frontal cortex (BA9) (Brain) −0.0102
GTEx: Hippocampus (Brain) −0.01818
GTEx: Hypothalamus (Brain) −0.01909
GTEx: Nucleus accumbens (basal ganglia) (Brain) −0.00989
GTEx: Putamen basal ganglia (Brain) −0.02089
GTEx: Breast mammary tissue −0.02428
GTEx: Sigmoid colon −0.0368
GTEx: Transverse colon −0.0615
GTEx: Gastroesophageal junction −0.01122
GTEx: Esophagus mucosa −0.01724
GTEx: Esophagus Muscularis −0.07986
GTEx: Atrial appendage (Heart) −0.07791
GTEx: Left ventricle (Heart) −0.001626
GTEx: Lung −0.03236
GTEx: Skeletal muscle −0.006367
GTEx: Tibial Nerve 0.001518
GTEx: Pancreas 0.009094
GTEx: Transformed fibroblasts −0.02666
GTEx: Sun exposed skin −0.005565
GTEx: Stomach −0.01017
GTEx: Testes −0.007513
GTEx: Thyroid −0.04886
CARTaGENE: Whole Blood −0.02082
GTEx: Whole Blood −0.02283
TwinsUK: Whole Blood −0.01734198
GTEx: Lymphoblastoid cell lines −0.02624
TwinsUK: Lymphoblastoid cell lines −0.02936791
TwinsUK: Subcutaneous Adipose 0.006941982
TwinsUK: Non−sun exposed skin −0.006247803
−0.15 −0.125 −0.1 −0.075 −0.05 −0.025 0 0.025 0.05 0.075
Beta Coefficient

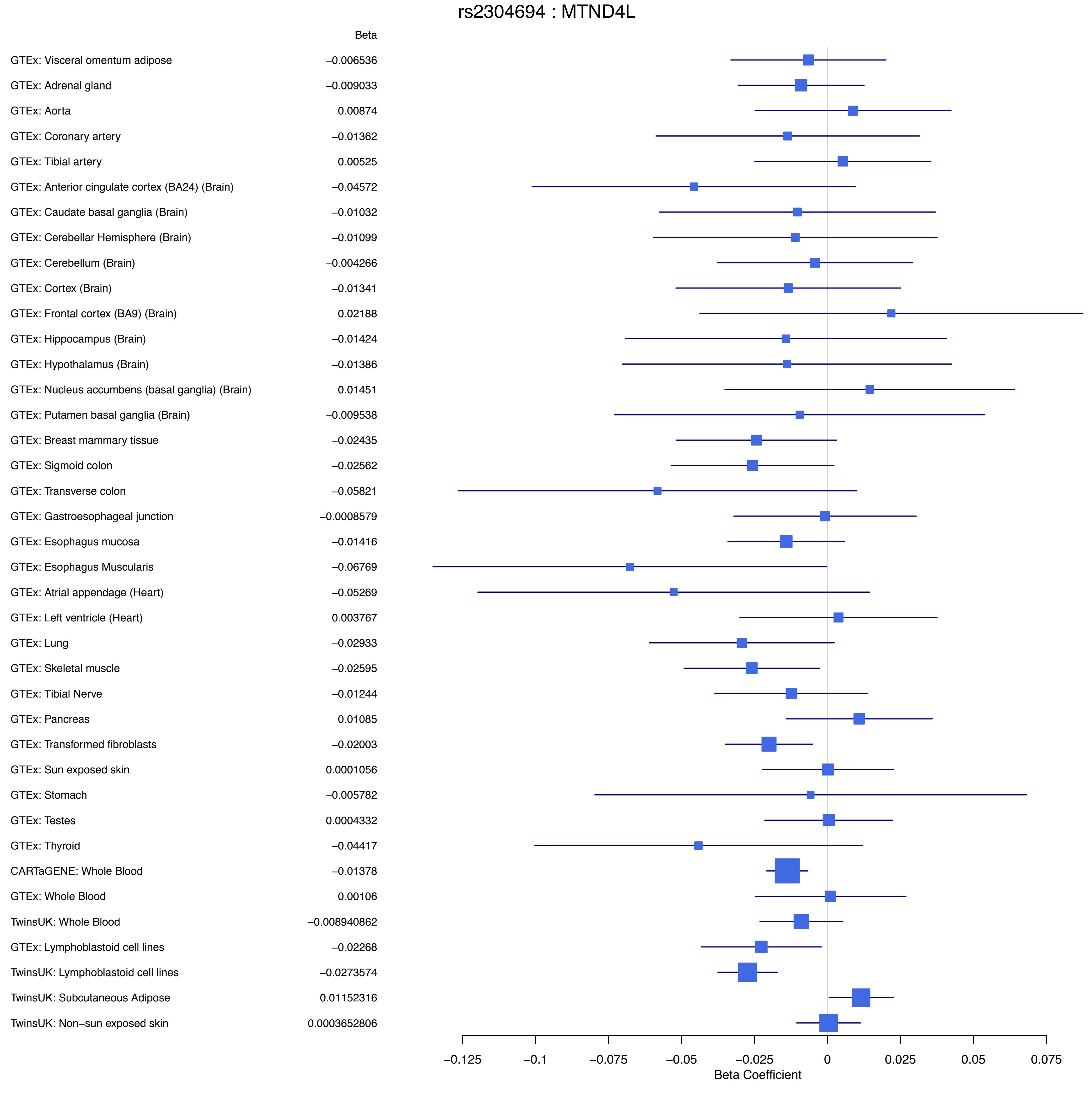
rs2304694 : MTND4L
Beta
GTEx: Visceral omentum adipose −0.006536
GTEx: Adrenal gland −0.009033
GTEx: Aorta 0.00874
GTEx: Coronary artery −0.01362
GTEx: Tibial artery 0.00525
GTEx: Anterior cingulate cortex (BA24) (Brain) −0.04572
GTEx: Caudate basal ganglia (Brain) −0.01032
GTEx: Cerebellar Hemisphere (Brain) −0.01099
GTEx: Cerebellum (Brain) −0.004266
GTEx: Cortex (Brain) −0.01341
GTEx: Frontal cortex (BA9) (Brain) 0.02188
GTEx: Hippocampus (Brain) −0.01424
GTEx: Hypothalamus (Brain) −0.01386
GTEx: Nucleus accumbens (basal ganglia) (Brain) 0.01451
GTEx: Putamen basal ganglia (Brain) −0.009538
GTEx: Breast mammary tissue −0.02435
GTEx: Sigmoid colon −0.02562
GTEx: Transverse colon −0.05821
GTEx: Gastroesophageal junction −0.0008579
GTEx: Esophagus mucosa −0.01416
GTEx: Esophagus Muscularis −0.06769
GTEx: Atrial appendage (Heart) −0.05269
GTEx: Left ventricle (Heart) 0.003767
GTEx: Lung −0.02933
GTEx: Skeletal muscle −0.02595
GTEx: Tibial Nerve −0.01244
GTEx: Pancreas 0.01085
GTEx: Transformed fibroblasts −0.02003
GTEx: Sun exposed skin 0.0001056
GTEx: Stomach −0.005782
GTEx: Testes 0.0004332
GTEx: Thyroid −0.04417
CARTaGENE: Whole Blood −0.01378
GTEx: Whole Blood 0.00106
TwinsUK: Whole Blood −0.008940862
GTEx: Lymphoblastoid cell lines −0.02268
TwinsUK: Lymphoblastoid cell lines −0.0273574
TwinsUK: Subcutaneous Adipose 0.01152316
TwinsUK: Non−sun exposed skin 0.0003652806
−0.125
−0.1
−0.075
−0.05
−0.025
0
0.025
0.05
0.075
Beta Coefficient

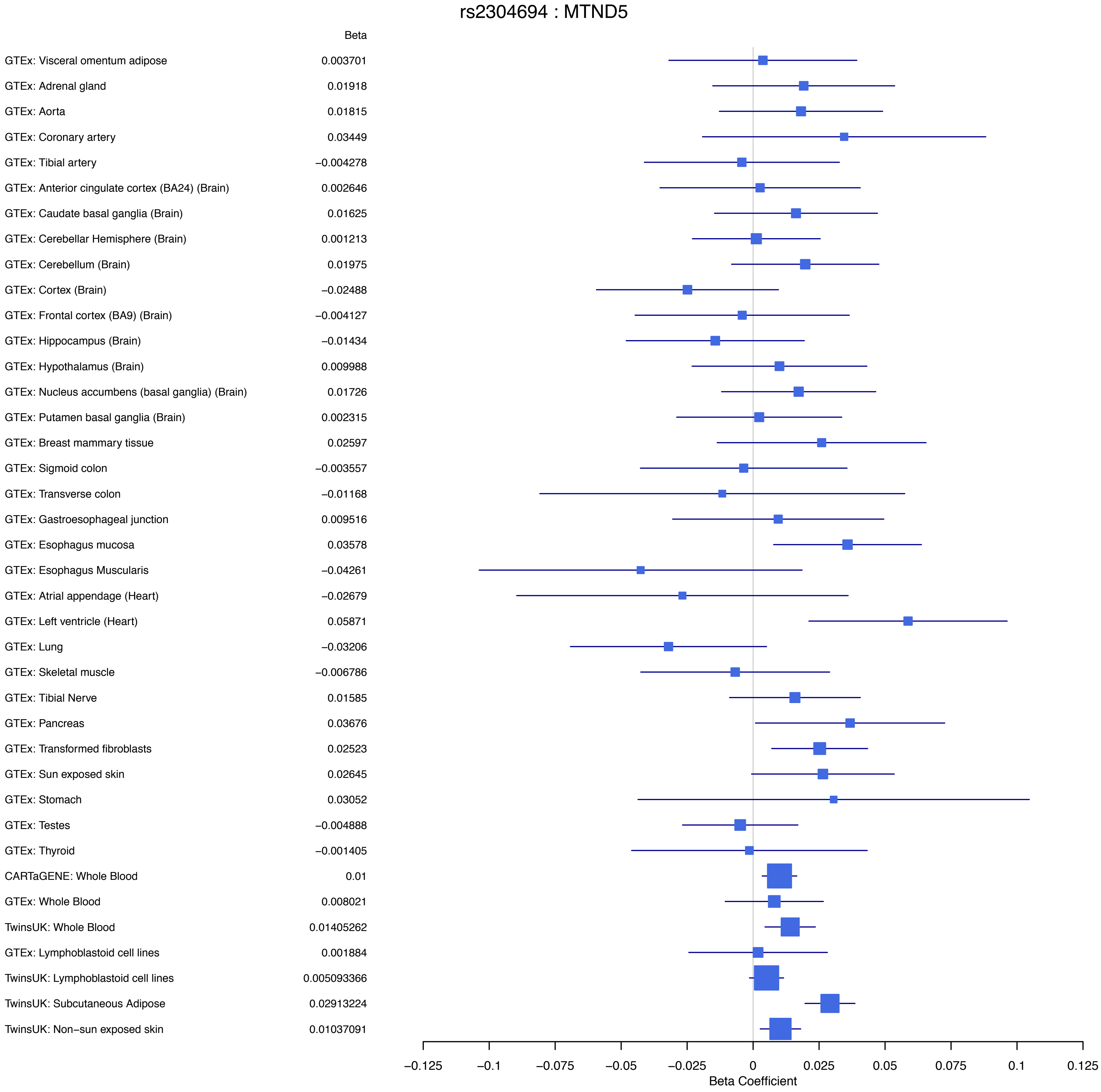
rs2304694 : MTND5
Beta
GTEx: Visceral omentum adipose 0.003701
GTEx: Adrenal gland 0.01918
GTEx: Aorta 0.01815
GTEx: Coronary artery 0.03449
GTEx: Tibial artery −0.004278
GTEx: Anterior cingulate cortex (BA24) (Brain) 0.002646
GTEx: Caudate basal ganglia (Brain) 0.01625
GTEx: Cerebellar Hemisphere (Brain) 0.001213
GTEx: Cerebellum (Brain) 0.01975
GTEx: Cortex (Brain) −0.02488
GTEx: Frontal cortex (BA9) (Brain) −0.004127
GTEx: Hippocampus (Brain) −0.01434
GTEx: Hypothalamus (Brain) 0.009988
GTEx: Nucleus accumbens (basal ganglia) (Brain) 0.01726
GTEx: Putamen basal ganglia (Brain) 0.002315
GTEx: Breast mammary tissue 0.02597
GTEx: Sigmoid colon −0.003557
GTEx: Transverse colon −0.01168
GTEx: Gastroesophageal junction 0.009516
GTEx: Esophagus mucosa 0.03578
GTEx: Esophagus Muscularis −0.04261
GTEx: Atrial appendage (Heart) −0.02679
GTEx: Left ventricle (Heart) 0.05871
GTEx: Lung −0.03206
GTEx: Skeletal muscle −0.006786
GTEx: Tibial Nerve 0.01585
GTEx: Pancreas 0.03676
GTEx: Transformed fibroblasts 0.02523
GTEx: Sun exposed skin 0.02645
GTEx: Stomach 0.03052
GTEx: Testes −0.004888
GTEx: Thyroid −0.001405
CARTaGENE: Whole Blood 0.01
GTEx: Whole Blood 0.008021
TwinsUK: Whole Blood 0.01405262
GTEx: Lymphoblastoid cell lines 0.001884
TwinsUK: Lymphoblastoid cell lines 0.005093366
TwinsUK: Subcutaneous Adipose 0.02913224
TwinsUK: Non−sun exposed skin 0.01037091
−0.125
−0.1
−0.075
−0.05
−0.025
0
0.025
0.05
0.075
0.1
0.125
Beta Coefficient

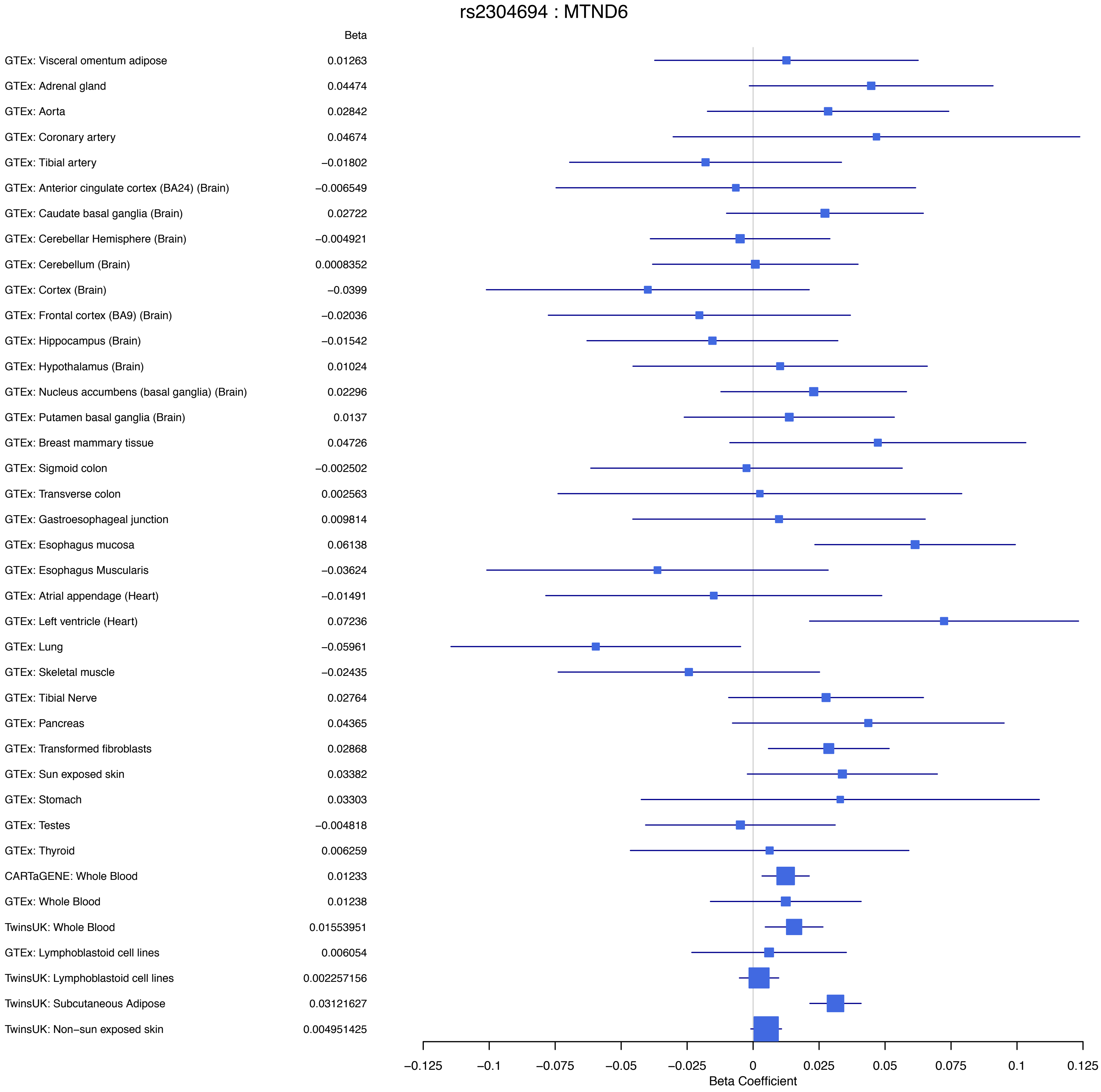
rs2304694 : MTND6
Beta
GTEx: Visceral omentum adipose 0.01263
GTEx: Adrenal gland 0.04474
GTEx: Aorta 0.02842
GTEx: Coronary artery 0.04674
GTEx: Tibial artery −0.01802
GTEx: Anterior cingulate cortex (BA24) (Brain) −0.006549
GTEx: Caudate basal ganglia (Brain) 0.02722
GTEx: Cerebellar Hemisphere (Brain) −0.004921
GTEx: Cerebellum (Brain) 0.0008352
GTEx: Cortex (Brain) −0.0399
GTEx: Frontal cortex (BA9) (Brain) −0.02036
GTEx: Hippocampus (Brain) −0.01542
GTEx: Hypothalamus (Brain) 0.01024
GTEx: Nucleus accumbens (basal ganglia) (Brain) 0.02296
GTEx: Putamen basal ganglia (Brain) 0.0137
GTEx: Breast mammary tissue 0.04726
GTEx: Sigmoid colon −0.002502
GTEx: Transverse colon 0.002563
GTEx: Gastroesophageal junction 0.009814
GTEx: Esophagus mucosa 0.06138
GTEx: Esophagus Muscularis −0.03624
GTEx: Atrial appendage (Heart) −0.01491
GTEx: Left ventricle (Heart) 0.07236
GTEx: Lung −0.05961
GTEx: Skeletal muscle −0.02435
GTEx: Tibial Nerve 0.02764
GTEx: Pancreas 0.04365
GTEx: Transformed fibroblasts 0.02868
GTEx: Sun exposed skin 0.03382
GTEx: Stomach 0.03303
GTEx: Testes −0.004818
GTEx: Thyroid 0.006259
CARTaGENE: Whole Blood 0.01233
GTEx: Whole Blood 0.01238
TwinsUK: Whole Blood 0.01553951
GTEx: Lymphoblastoid cell lines 0.006054
TwinsUK: Lymphoblastoid cell lines 0.002257156
TwinsUK: Subcutaneous Adipose 0.03121627
TwinsUK: Non−sun exposed skin 0.004951425
−0.125
−0.1
−0.075
−0.05
−0.025
0
0.025
0.05
0.075
0.1
0.125
Beta Coefficient

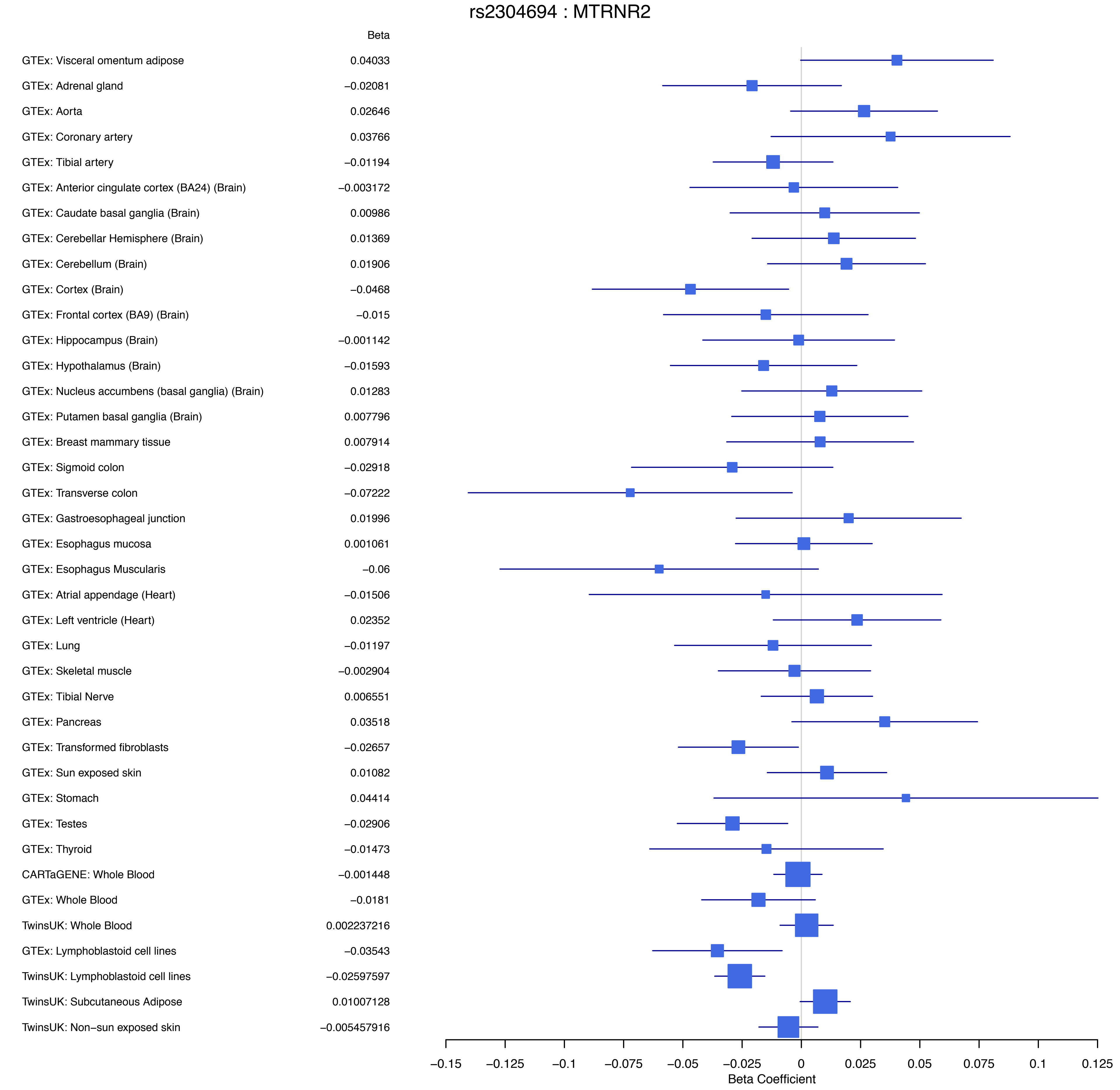
rs2304694 : MTRNR2
Beta
GTEx: Visceral omentum adipose 0.04033
GTEx: Adrenal gland −0.02081
GTEx: Aorta 0.02646
GTEx: Coronary artery 0.03766
GTEx: Tibial artery −0.01194
GTEx: Anterior cingulate cortex (BA24) (Brain) −0.003172
GTEx: Caudate basal ganglia (Brain) 0.00986
GTEx: Cerebellar Hemisphere (Brain) 0.01369
GTEx: Cerebellum (Brain) 0.01906
GTEx: Cortex (Brain) −0.0468
GTEx: Frontal cortex (BA9) (Brain) −0.015
GTEx: Hippocampus (Brain) −0.001142
GTEx: Hypothalamus (Brain) −0.01593
GTEx: Nucleus accumbens (basal ganglia) (Brain) 0.01283
GTEx: Putamen basal ganglia (Brain) 0.007796
GTEx: Breast mammary tissue 0.007914
GTEx: Sigmoid colon −0.02918
GTEx: Transverse colon −0.07222
GTEx: Gastroesophageal junction 0.01996
GTEx: Esophagus mucosa 0.001061
GTEx: Esophagus Muscularis −0.06
GTEx: Atrial appendage (Heart) −0.01506
GTEx: Left ventricle (Heart) 0.02352
GTEx: Lung −0.01197
GTEx: Skeletal muscle −0.002904
GTEx: Tibial Nerve 0.006551
GTEx: Pancreas 0.03518
GTEx: Transformed fibroblasts −0.02657
GTEx: Sun exposed skin 0.01082
GTEx: Stomach 0.04414
GTEx: Testes −0.02906
GTEx: Thyroid −0.01473
CARTaGENE: Whole Blood −0.001448
GTEx: Whole Blood −0.0181
TwinsUK: Whole Blood 0.002237216
GTEx: Lymphoblastoid cell lines −0.03543
TwinsUK: Lymphoblastoid cell lines −0.02597597
TwinsUK: Subcutaneous Adipose 0.01007128
TwinsUK: Non−sun exposed skin −0.005457916
−0.15 −0.125 −0.1 −0.075 −0.05 −0.025 0 0.025 0.05 0.075 0.1 0.125
Beta Coefficient

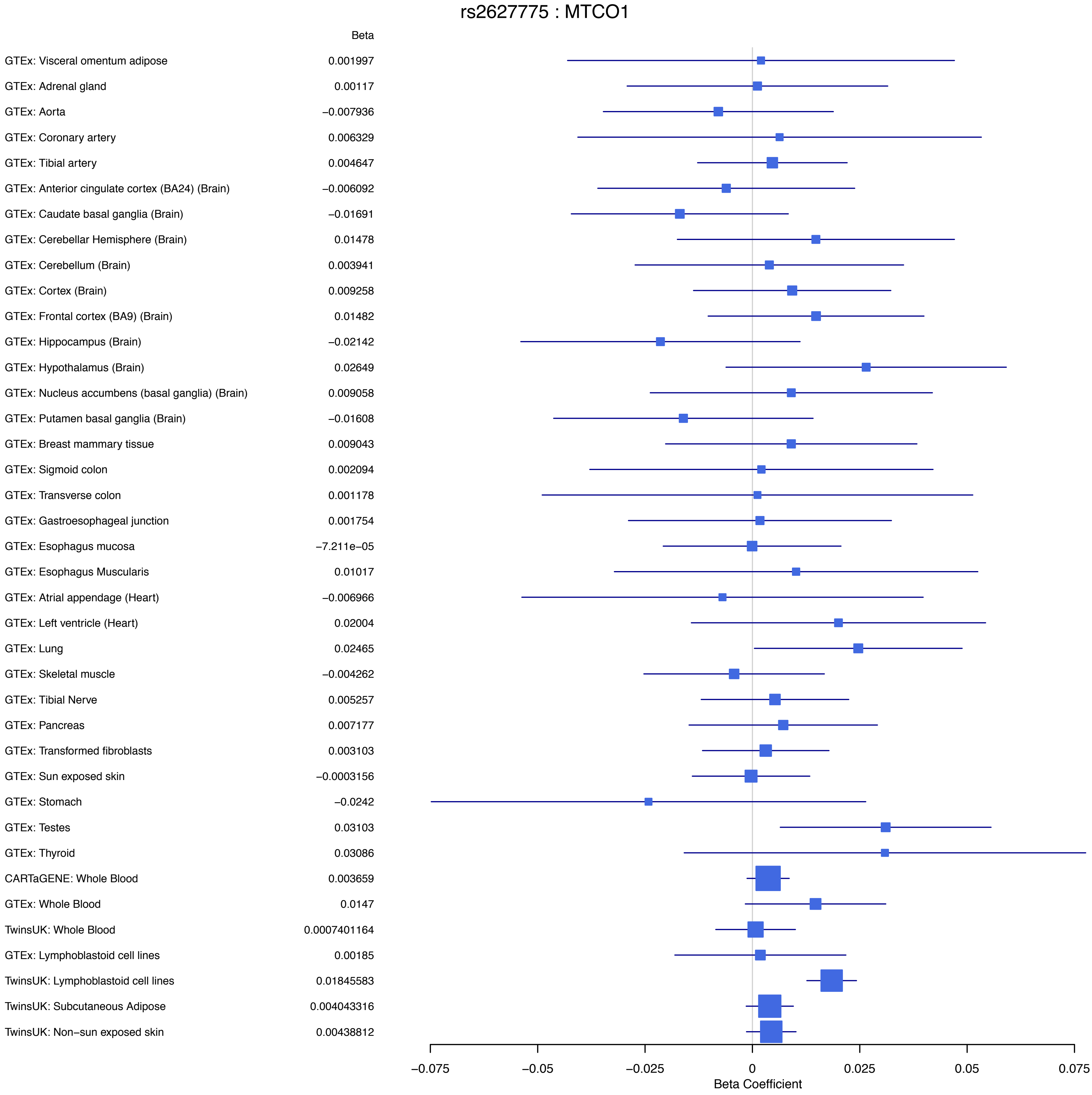
rs2627775 : MTCO1
Beta
GTEx: Visceral omentum adipose 0.001997
GTEx: Adrenal gland 0.00117
GTEx: Aorta −0.007936
GTEx: Coronary artery 0.006329
GTEx: Tibial artery 0.004647
GTEx: Anterior cingulate cortex (BA24) (Brain) −0.006092
GTEx: Caudate basal ganglia (Brain) −0.01691
GTEx: Cerebellar Hemisphere (Brain) 0.01478
GTEx: Cerebellum (Brain) 0.003941
GTEx: Cortex (Brain) 0.009258
GTEx: Frontal cortex (BA9) (Brain) 0.01482
GTEx: Hippocampus (Brain) −0.02142
GTEx: Hypothalamus (Brain) 0.02649
GTEx: Nucleus accumbens (basal ganglia) (Brain) 0.009058
GTEx: Putamen basal ganglia (Brain) −0.01608
GTEx: Breast mammary tissue 0.009043
GTEx: Sigmoid colon 0.002094
GTEx: Transverse colon 0.001178
GTEx: Gastroesophageal junction 0.001754
GTEx: Esophagus mucosa −7.211e−05
GTEx: Esophagus Muscularis 0.01017
GTEx: Atrial appendage (Heart) −0.006966
GTEx: Left ventricle (Heart) 0.02004
GTEx: Lung 0.02465
GTEx: Skeletal muscle −0.004262
GTEx: Tibial Nerve 0.005257
GTEx: Pancreas 0.007177
GTEx: Transformed fibroblasts 0.003103
GTEx: Sun exposed skin −0.0003156
GTEx: Stomach −0.0242
GTEx: Testes 0.03103
GTEx: Thyroid 0.03086
CARTaGENE: Whole Blood 0.003659
GTEx: Whole Blood 0.0147
TwinsUK: Whole Blood 0.0007401164
GTEx: Lymphoblastoid cell lines 0.00185
TwinsUK: Lymphoblastoid cell lines 0.01845583
TwinsUK: Subcutaneous Adipose 0.004043316
TwinsUK: Non−sun exposed skin 0.00438812
−0.075
−0.05
−0.025
0
0.025
0.05
0.075
Beta Coefficient

rs2712498 : MTND3

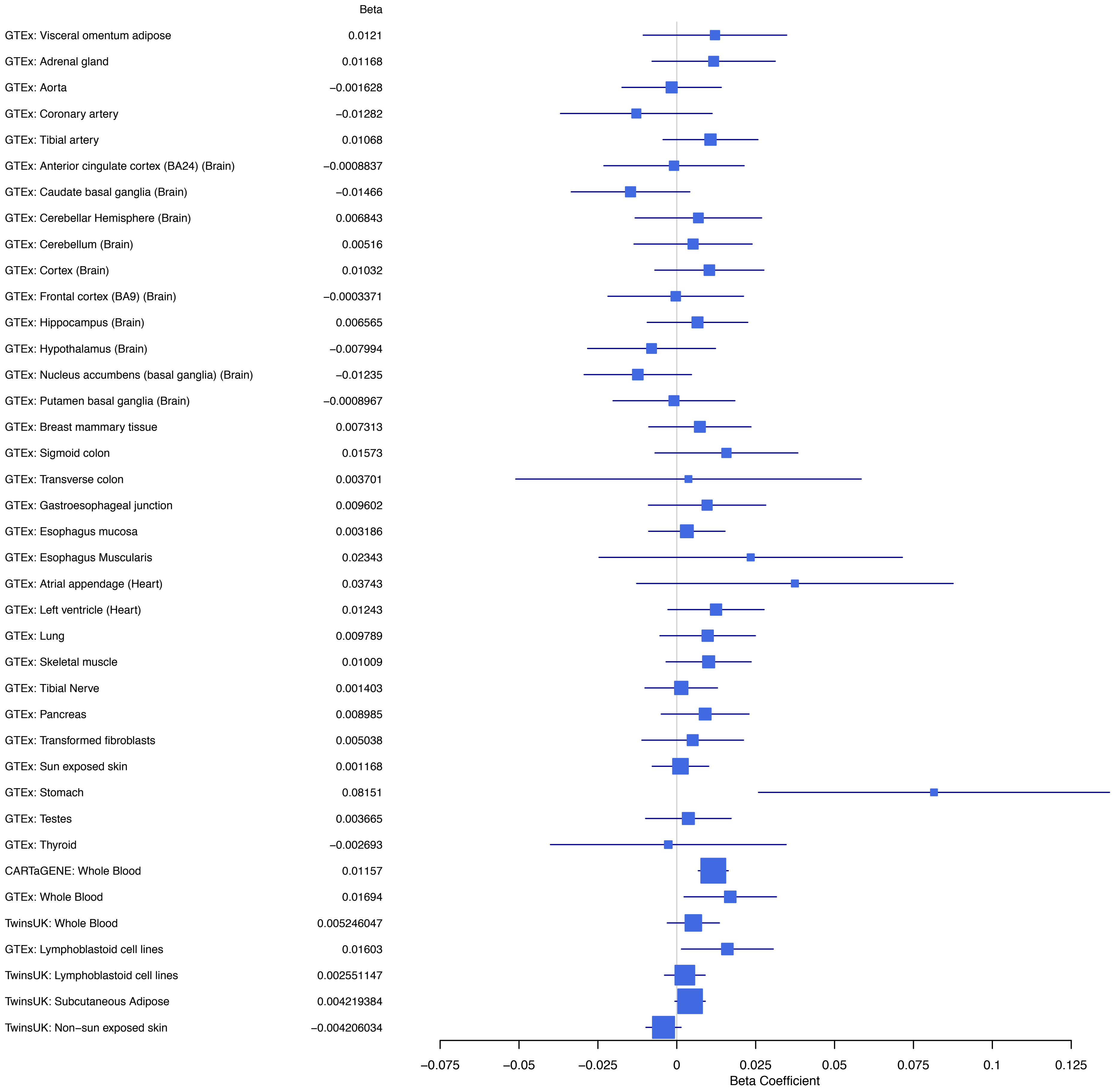

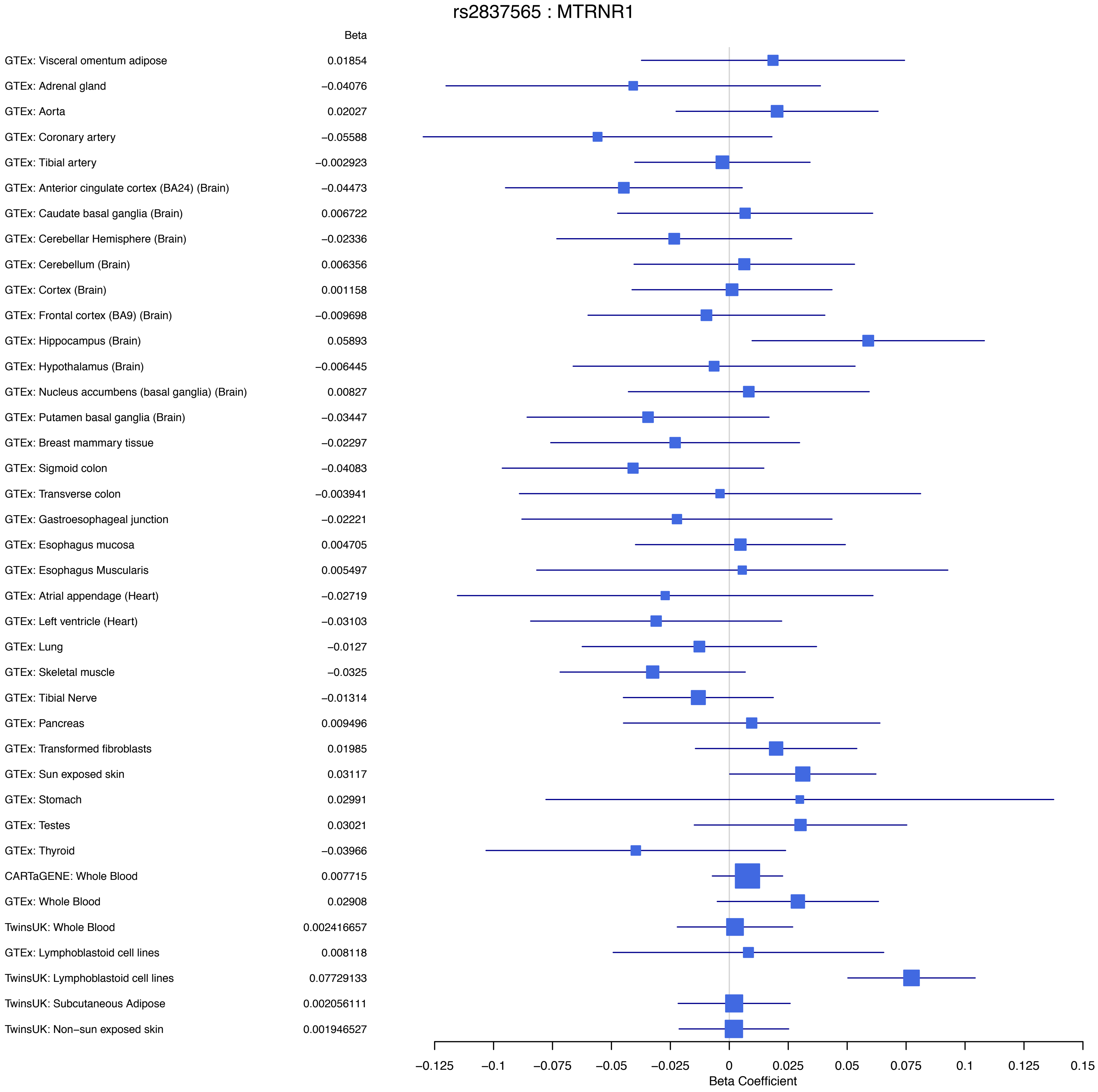
rs2837565 : MTRNR1
Beta
GTEx: Visceral omentum adipose 0.01854
GTEx: Adrenal gland −0.04076
GTEx: Aorta 0.02027
GTEx: Coronary artery −0.05588
GTEx: Tibial artery −0.002923
GTEx: Anterior cingulate cortex (BA24) (Brain) −0.04473
GTEx: Caudate basal ganglia (Brain) 0.006722
GTEx: Cerebellar Hemisphere (Brain) −0.02336
GTEx: Cerebellum (Brain) 0.006356
GTEx: Cortex (Brain) 0.001158
GTEx: Frontal cortex (BA9) (Brain) −0.009698
GTEx: Hippocampus (Brain) 0.05893
GTEx: Hypothalamus (Brain) −0.006445
GTEx: Nucleus accumbens (basal ganglia) (Brain) 0.00827
GTEx: Putamen basal ganglia (Brain) −0.03447
GTEx: Breast mammary tissue −0.02297
GTEx: Sigmoid colon −0.04083
GTEx: Transverse colon −0.003941
GTEx: Gastroesophageal junction −0.02221
GTEx: Esophagus mucosa 0.004705
GTEx: Esophagus Muscularis 0.005497
GTEx: Atrial appendage (Heart) −0.02719
GTEx: Left ventricle (Heart) −0.03103
GTEx: Lung −0.0127
GTEx: Skeletal muscle −0.0325
GTEx: Tibial Nerve −0.01314
GTEx: Pancreas 0.009496
GTEx: Transformed fibroblasts 0.01985
GTEx: Sun exposed skin 0.03117
GTEx: Stomach 0.02991
GTEx: Testes 0.03021
GTEx: Thyroid −0.03966
CARTaGENE: Whole Blood 0.007715
GTEx: Whole Blood 0.02908
TwinsUK: Whole Blood 0.002416657
GTEx: Lymphoblastoid cell lines 0.008118
TwinsUK: Lymphoblastoid cell lines 0.07729133
TwinsUK: Subcutaneous Adipose 0.002056111
TwinsUK: Non−sun exposed skin 0.001946527
−0.125 −0.1 −0.075 −0.05 −0.025 0 0.025 0.05 0.075 0.1 0.125 0.15
Beta Coefficient

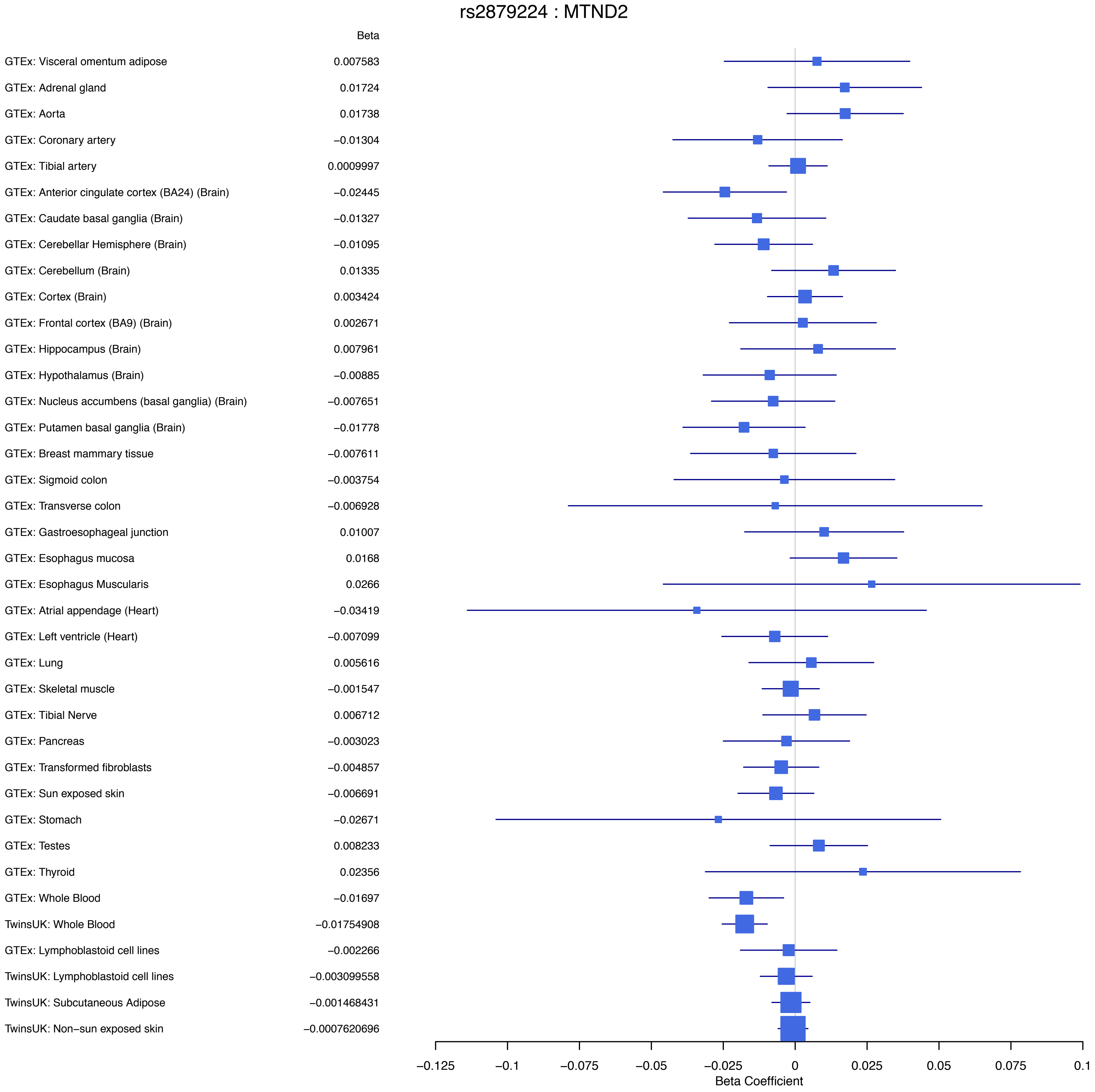
rs2879224 : MTND2
Beta
GTEx: Visceral omentum adipose 0.007583
GTEx: Adrenal gland 0.01724
GTEx: Aorta 0.01738
GTEx: Coronary artery −0.01304
GTEx: Tibial artery 0.0009997
GTEx: Anterior cingulate cortex (BA24) (Brain) −0.02445
GTEx: Caudate basal ganglia (Brain) −0.01327
GTEx: Cerebellar Hemisphere (Brain) −0.01095
GTEx: Cerebellum (Brain) 0.01335
GTEx: Cortex (Brain) 0.003424
GTEx: Frontal cortex (BA9) (Brain) 0.002671
GTEx: Hippocampus (Brain) 0.007961
GTEx: Hypothalamus (Brain) −0.00885
GTEx: Nucleus accumbens (basal ganglia) (Brain) −0.007651
GTEx: Putamen basal ganglia (Brain) −0.01778
GTEx: Breast mammary tissue −0.007611
GTEx: Sigmoid colon −0.003754
GTEx: Transverse colon −0.006928
GTEx: Gastroesophageal junction 0.01007
GTEx: Esophagus mucosa 0.0168
GTEx: Esophagus Muscularis 0.0266
GTEx: Atrial appendage (Heart) −0.03419
GTEx: Left ventricle (Heart) −0.007099
GTEx: Lung 0.005616
GTEx: Skeletal muscle −0.001547
GTEx: Tibial Nerve 0.006712
GTEx: Pancreas −0.003023
GTEx: Transformed fibroblasts −0.004857
GTEx: Sun exposed skin −0.006691
GTEx: Stomach −0.02671
GTEx: Testes 0.008233
GTEx: Thyroid 0.02356
GTEx: Whole Blood −0.01697
TwinsUK: Whole Blood −0.01754908
GTEx: Lymphoblastoid cell lines −0.002266
TwinsUK: Lymphoblastoid cell lines −0.003099558
TwinsUK: Subcutaneous Adipose −0.001468431
TwinsUK: Non−sun exposed skin −0.0007620696
−0.125
−0.1
−0.075
−0.05
−0.025
0
0.025
0.05
0.075
0.1
Beta Coefficient

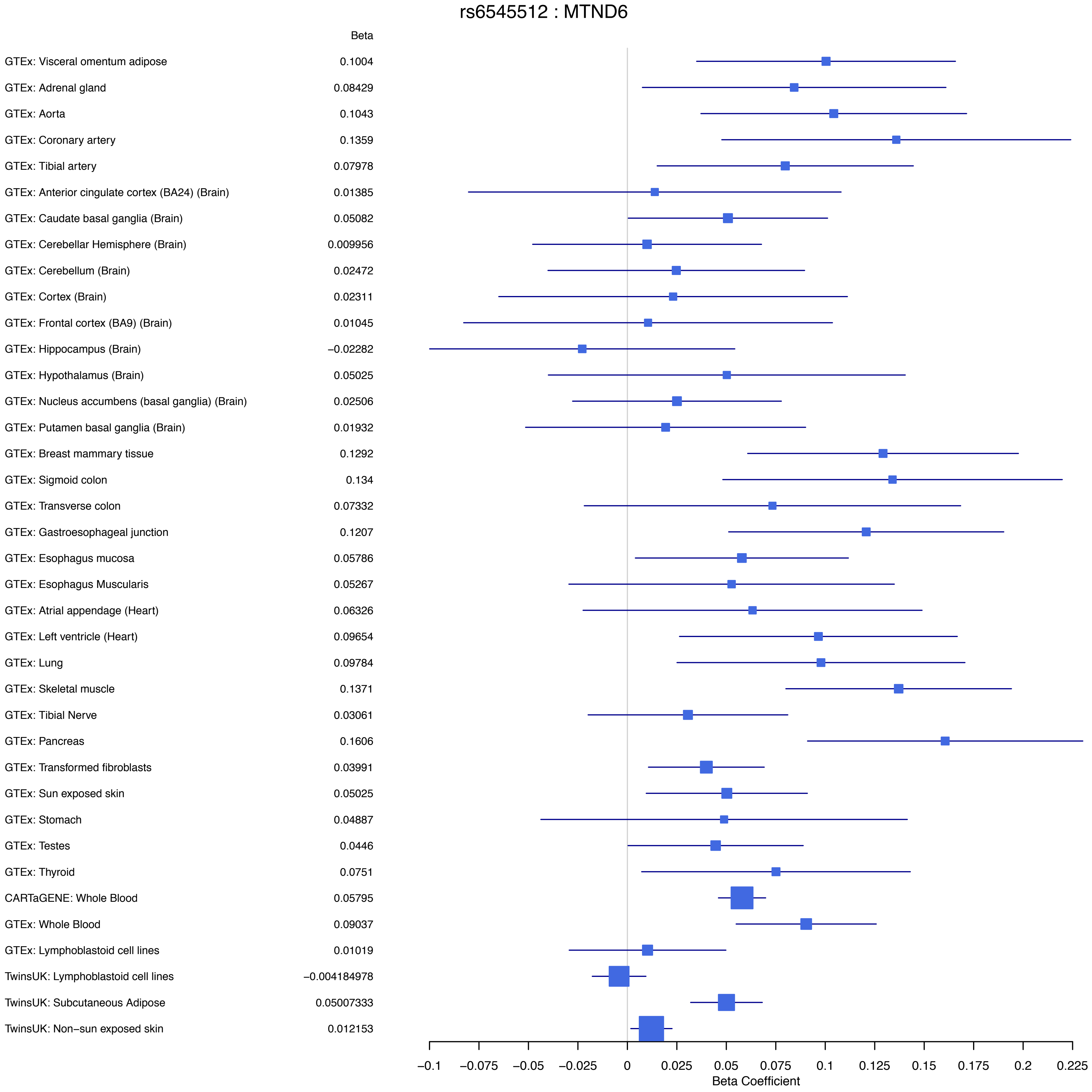
rs6545512 : MTND6
Beta
GTEx: Visceral omentum adipose 0.1004
GTEx: Adrenal gland 0.08429
GTEx: Aorta 0.1043
GTEx: Coronary artery 0.1359
GTEx: Tibial artery 0.07978
GTEx: Anterior cingulate cortex (BA24) (Brain) 0.01385
GTEx: Caudate basal ganglia (Brain) 0.05082
GTEx: Cerebellar Hemisphere (Brain) 0.009956
GTEx: Cerebellum (Brain) 0.02472
GTEx: Cortex (Brain) 0.02311
GTEx: Frontal cortex (BA9) (Brain) 0.01045
GTEx: Hippocampus (Brain) −0.02282
GTEx: Hypothalamus (Brain) 0.05025
GTEx: Nucleus accumbens (basal ganglia) (Brain) 0.02506
GTEx: Putamen basal ganglia (Brain) 0.01932
GTEx: Breast mammary tissue 0.1292
GTEx: Sigmoid colon 0.134
GTEx: Transverse colon 0.07332
GTEx: Gastroesophageal junction 0.1207
GTEx: Esophagus mucosa 0.05786
GTEx: Esophagus Muscularis 0.05267
GTEx: Atrial appendage (Heart) 0.06326
GTEx: Left ventricle (Heart) 0.09654
GTEx: Lung 0.09784
GTEx: Skeletal muscle 0.1371
GTEx: Tibial Nerve 0.03061
GTEx: Pancreas 0.1606
GTEx: Transformed fibroblasts 0.03991
GTEx: Sun exposed skin 0.05025
GTEx: Stomach 0.04887
GTEx: Testes 0.0446
GTEx: Thyroid 0.0751
CARTaGENE: Whole Blood 0.05795
GTEx: Whole Blood 0.09037
GTEx: Lymphoblastoid cell lines 0.01019
TwinsUK: Lymphoblastoid cell lines −0.004184978
TwinsUK: Subcutaneous Adipose 0.05007333
TwinsUK: Non−sun exposed skin 0.012153
−0.1
−0.075
−0.05
−0.025
0
0.025
0.05
0.075
0.1
0.125
0.15
0.175
0.2
0.225
Beta Coefficient

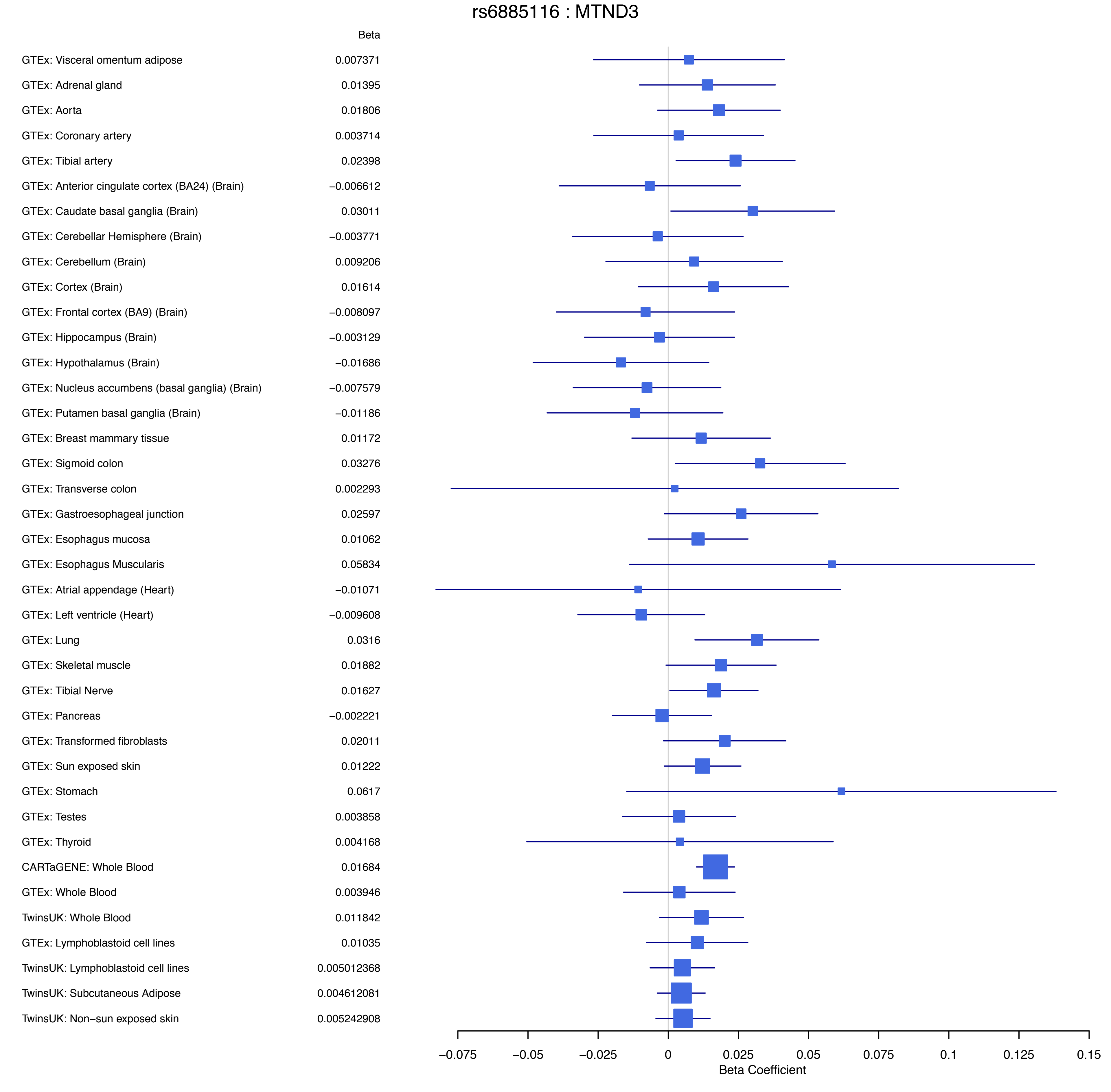
rs6885116 : MTND3
Beta
GTEx: Visceral omentum adipose 0.007371
GTEx: Adrenal gland 0.01395
GTEx: Aorta 0.01806
GTEx: Coronary artery 0.003714
GTEx: Tibial artery 0.02398
GTEx: Anterior cingulate cortex (BA24) (Brain) −0.006612
GTEx: Caudate basal ganglia (Brain) 0.03011
GTEx: Cerebellar Hemisphere (Brain) −0.003771
GTEx: Cerebellum (Brain) 0.009206
GTEx: Cortex (Brain) 0.01614
GTEx: Frontal cortex (BA9) (Brain) −0.008097
GTEx: Hippocampus (Brain) −0.003129
GTEx: Hypothalamus (Brain) −0.01686
GTEx: Nucleus accumbens (basal ganglia) (Brain) −0.007579
GTEx: Putamen basal ganglia (Brain) −0.01186
GTEx: Breast mammary tissue 0.01172
GTEx: Sigmoid colon 0.03276
GTEx: Transverse colon 0.002293
GTEx: Gastroesophageal junction 0.02597
GTEx: Esophagus mucosa 0.01062
GTEx: Esophagus Muscularis 0.05834
GTEx: Atrial appendage (Heart) −0.01071
GTEx: Left ventricle (Heart) −0.009608
GTEx: Lung 0.0316
GTEx: Skeletal muscle 0.01882
GTEx: Tibial Nerve 0.01627
GTEx: Pancreas −0.002221
GTEx: Transformed fibroblasts 0.02011
GTEx: Sun exposed skin 0.01222
GTEx: Stomach 0.0617
GTEx: Testes 0.003858
GTEx: Thyroid 0.004168
CARTaGENE: Whole Blood 0.01684
GTEx: Whole Blood 0.003946
TwinsUK: Whole Blood 0.011842
GTEx: Lymphoblastoid cell lines 0.01035
TwinsUK: Lymphoblastoid cell lines 0.005012368
TwinsUK: Subcutaneous Adipose 0.004612081
TwinsUK: Non−sun exposed skin 0.005242908
−0.075
−0.05
−0.025
0
0.025
0.05
0.075
0.1
0.125
0.15
Beta Coefficient

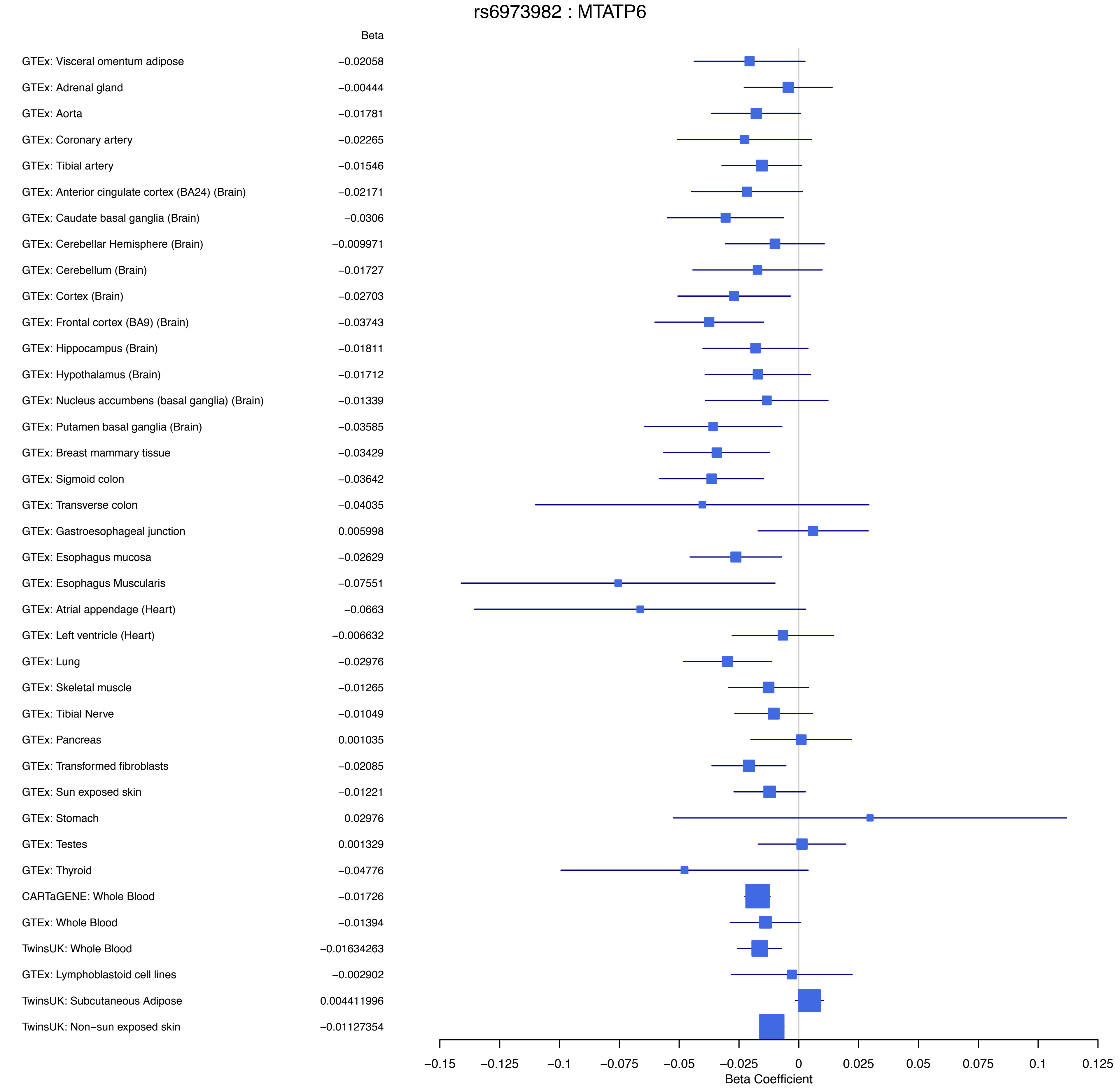
rs6973982 : MTATP6
Beta
GTEx: Visceral omentum adipose −0.02058
GTEx: Adrenal gland −0.00444
GTEx: Aorta −0.01781
GTEx: Coronary artery −0.02265
GTEx: Tibial artery −0.01546
GTEx: Anterior cingulate cortex (BA24) (Brain) −0.02171
GTEx: Caudate basal ganglia (Brain) −0.0306
GTEx: Cerebellar Hemisphere (Brain) −0.009971
GTEx: Cerebellum (Brain) −0.01727
GTEx: Cortex (Brain) −0.02703
GTEx: Frontal cortex (BA9) (Brain) −0.03743
GTEx: Hippocampus (Brain) −0.01811
GTEx: Hypothalamus (Brain) −0.01712
GTEx: Nucleus accumbens (basal ganglia) (Brain) −0.01339
GTEx: Putamen basal ganglia (Brain) −0.03585
GTEx: Breast mammary tissue −0.03429
GTEx: Sigmoid colon −0.03642
GTEx: Transverse colon −0.04035
GTEx: Gastroesophageal junction 0.005998
GTEx: Esophagus mucosa −0.02629
GTEx: Esophagus Muscularis −0.07551
GTEx: Atrial appendage (Heart) −0.0663
GTEx: Left ventricle (Heart) −0.006632
GTEx: Lung −0.02976
GTEx: Skeletal muscle −0.01265
GTEx: Tibial Nerve −0.01049
GTEx: Pancreas 0.001035
GTEx: Transformed fibroblasts −0.02085
GTEx: Sun exposed skin −0.01221
GTEx: Stomach 0.02976
GTEx: Testes 0.001329
GTEx: Thyroid −0.04776
CARTaGENE: Whole Blood −0.01726
GTEx: Whole Blood −0.01394
TwinsUK: Whole Blood −0.01634263
GTEx: Lymphoblastoid cell lines −0.002902
TwinsUK: Subcutaneous Adipose 0.004411996
TwinsUK: Non−sun exposed skin −0.01127354
−0.15 −0.125 −0.1 −0.075 −0.05 −0.025 0 0.025 0.05 0.075 0.1 0.125
Beta Coefficient

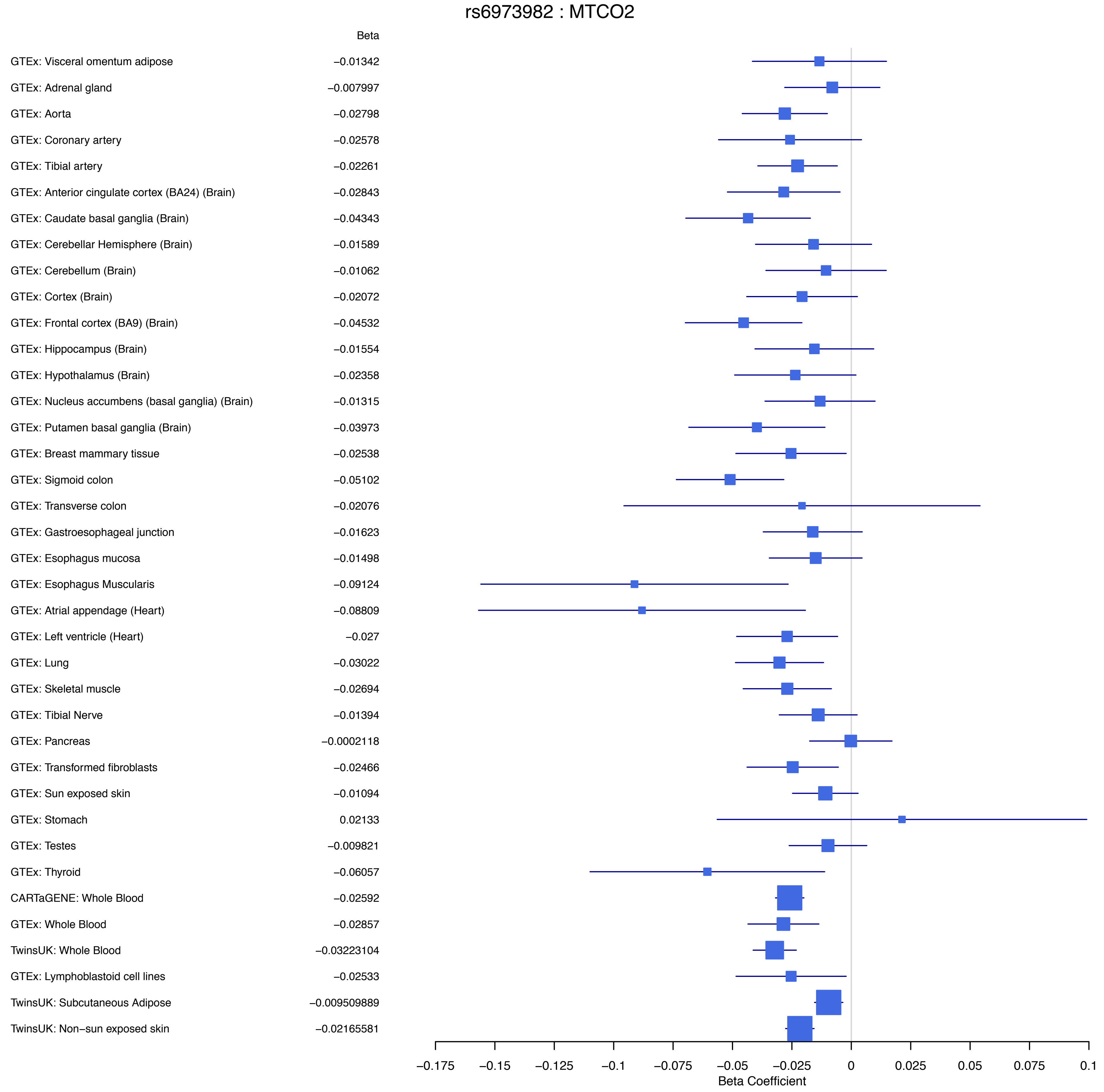
rs6973982 : MTCO2
Beta
GTEx: Visceral omentum adipose
−0.01342
GTEx: Adrenal gland
−0.007997
GTEx: Aorta
−0.02798
GTEx: Coronary artery
−0.02578
GTEx: Tibial artery
−0.02261
GTEx: Anterior cingulate cortex (BA24) (Brain)
−0.02843
GTEx: Caudate basal ganglia (Brain)
−0.04343
GTEx: Cerebellar Hemisphere (Brain)
−0.01589
GTEx: Cerebellum (Brain)
−0.01062
GTEx: Cortex (Brain)
−0.02072
GTEx: Frontal cortex (BA9) (Brain)
−0.04532
GTEx: Hippocampus (Brain)
−0.01554
GTEx: Hypothalamus (Brain)
−0.02358
GTEx: Nucleus accumbens (basal ganglia) (Brain)
−0.01315
GTEx: Putamen basal ganglia (Brain)
−0.03973
GTEx: Breast mammary tissue
−0.02538
GTEx: Sigmoid colon
−0.05102
GTEx: Transverse colon
−0.02076
GTEx: Gastroesophageal junction
−0.01623
GTEx: Esophagus mucosa
−0.01498
GTEx: Esophagus Muscularis
−0.09124
GTEx: Atrial appendage (Heart)
−0.08809
GTEx: Left ventricle (Heart)
−0.027
GTEx: Lung
−0.03022
GTEx: Skeletal muscle
−0.02694
GTEx: Tibial Nerve
−0.01394
GTEx: Pancreas
−0.0002118
GTEx: Transformed fibroblasts
−0.02466
GTEx: Sun exposed skin
−0.01094
GTEx: Stomach
0.02133
GTEx: Testes
−0.009821
GTEx: Thyroid
−0.06057
CARTaGENE: Whole Blood
−0.02592
GTEx: Whole Blood
−0.02857
TwinsUK: Whole Blood
−0.03223104
GTEx: Lymphoblastoid cell lines
−0.02533
TwinsUK: Subcutaneous Adipose
−0.009509889
TwinsUK: Non−sun exposed skin
−0.02165581
−0.175
−0.15
−0.125
−0.1
−0.075
−0.05
−0.025
0
0.025
0.05
0.075
0.1
Beta Coefficient

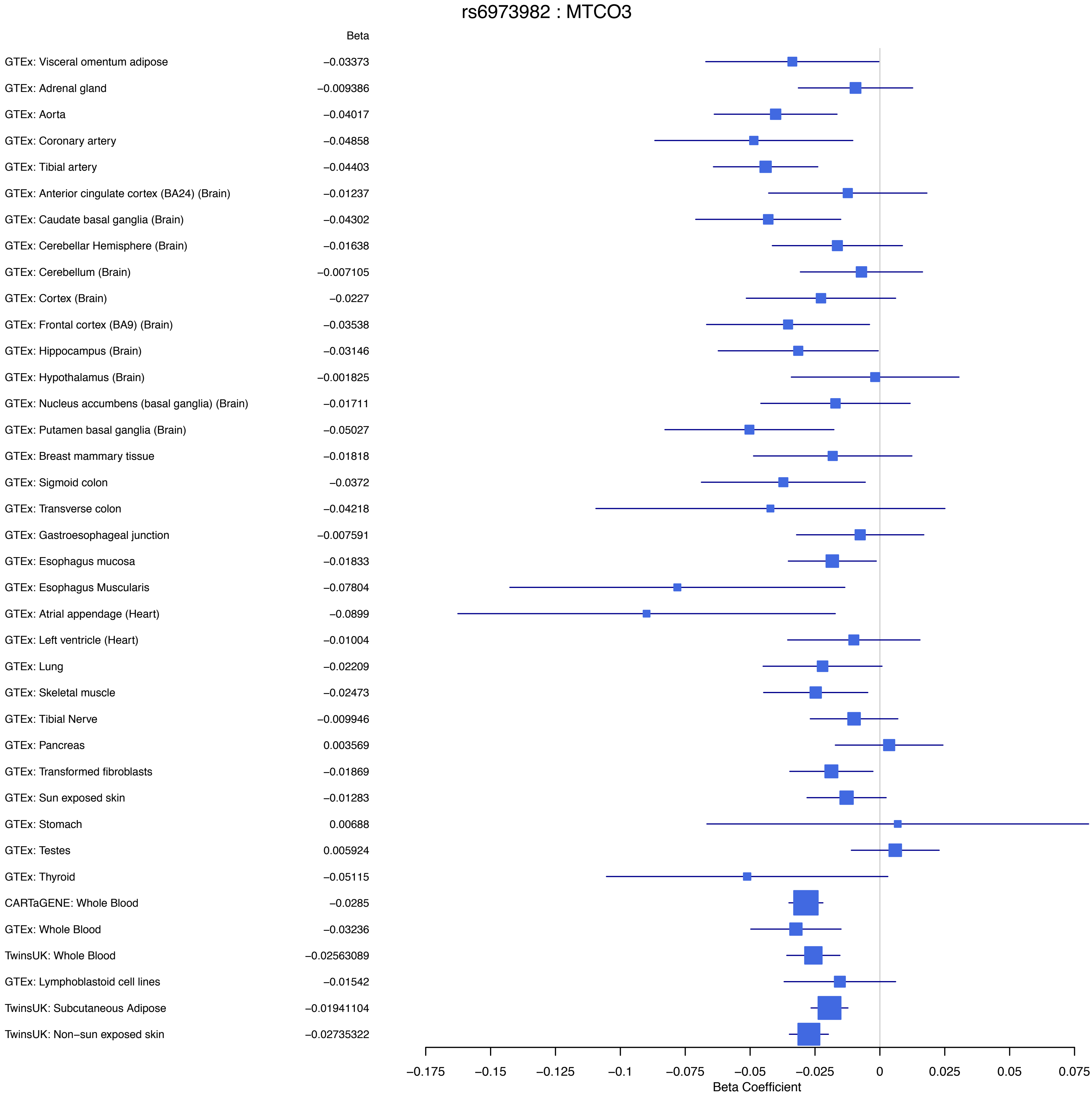
rs6973982 : MTCO3
Beta
GTEx: Visceral omentum adipose –0.03373
GTEx: Adrenal gland –0.009386
GTEx: Aorta –0.04017
GTEx: Coronary artery –0.04858
GTEx: Tibial artery –0.04403
GTEx: Anterior cingulate cortex (BA24) (Brain) –0.01237
GTEx: Caudate basal ganglia (Brain) –0.04302
GTEx: Cerebellar Hemisphere (Brain) –0.01638
GTEx: Cerebellum (Brain) –0.007105
GTEx: Cortex (Brain) –0.0227
GTEx: Frontal cortex (BA9) (Brain) –0.03538
GTEx: Hippocampus (Brain) –0.03146
GTEx: Hypothalamus (Brain) –0.001825
GTEx: Nucleus accumbens (basal ganglia) (Brain) –0.01711
GTEx: Putamen basal ganglia (Brain) –0.05027
GTEx: Breast mammary tissue –0.01818
GTEx: Sigmoid colon –0.0372
GTEx: Transverse colon –0.04218
GTEx: Gastroesophageal junction –0.007591
GTEx: Esophagus mucosa –0.01833
GTEx: Esophagus Muscularis –0.07804
GTEx: Atrial appendage (Heart) –0.0899
GTEx: Left ventricle (Heart) –0.01004
GTEx: Lung –0.02209
GTEx: Skeletal muscle –0.02473
GTEx: Tibial Nerve –0.009946
GTEx: Pancreas 0.003569
GTEx: Transformed fibroblasts –0.01869
GTEx: Sun exposed skin –0.01283
GTEx: Stomach 0.00688
GTEx: Testes 0.005924
GTEx: Thyroid –0.05115
CARTaGENE: Whole Blood –0.0285
GTEx: Whole Blood –0.03236
TwinsUK: Whole Blood –0.02563089
GTEx: Lymphoblastoid cell lines –0.01542
TwinsUK: Subcutaneous Adipose –0.01941104
TwinsUK: Non–sun exposed skin –0.02735322
–0.175 –0.15 –0.125 –0.1 –0.075 –0.05 –0.025 0 0.025 0.05 0.075
Beta Coefficient

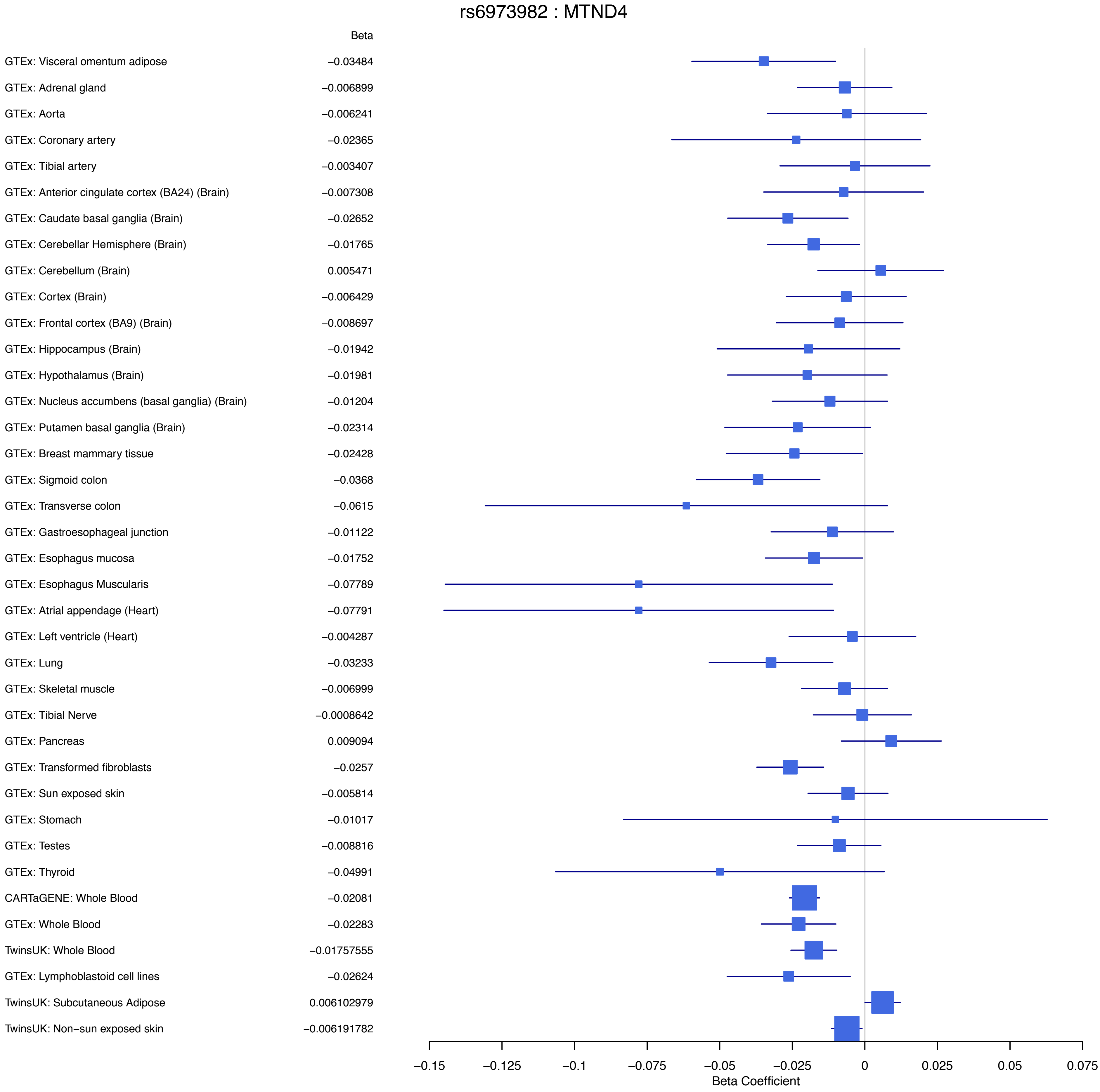

rs6973982 : MTND4
Beta
GTEx: Visceral omentum adipose −0.03484
GTEx: Adrenal gland −0.006899
GTEx: Aorta −0.006241
GTEx: Coronary artery −0.02365
GTEx: Tibial artery −0.003407
GTEx: Anterior cingulate cortex (BA24) (Brain) −0.007308
GTEx: Caudate basal ganglia (Brain) −0.02652
GTEx: Cerebellar Hemisphere (Brain) −0.01765
GTEx: Cerebellum (Brain) 0.005471
GTEx: Cortex (Brain) −0.006429
GTEx: Frontal cortex (BA9) (Brain) −0.008697
GTEx: Hippocampus (Brain) −0.01942
GTEx: Hypothalamus (Brain) −0.01981
GTEx: Nucleus accumbens (basal ganglia) (Brain) −0.01204
GTEx: Putamen basal ganglia (Brain) −0.02314
GTEx: Breast mammary tissue −0.02428
GTEx: Sigmoid colon −0.0368
GTEx: Transverse colon −0.0615
GTEx: Gastroesophageal junction −0.01122
GTEx: Esophagus mucosa −0.01752
GTEx: Esophagus Muscularis −0.07789
GTEx: Atrial appendage (Heart) −0.07791
GTEx: Left ventricle (Heart) −0.004287
GTEx: Lung −0.03233
GTEx: Skeletal muscle −0.006999
GTEx: Tibial Nerve −0.0008642
GTEx: Pancreas 0.009094
GTEx: Transformed fibroblasts −0.0257
GTEx: Sun exposed skin −0.005814
GTEx: Stomach −0.01017
GTEx: Testes −0.008816
GTEx: Thyroid −0.04991
CARTaGENE: Whole Blood −0.02081
GTEx: Whole Blood −0.02283
TwinsUK: Whole Blood −0.01757555
GTEx: Lymphoblastoid cell lines −0.02624
TwinsUK: Subcutaneous Adipose 0.006102979
TwinsUK: Non−sun exposed skin −0.006191782
−0.15 −0.125 −0.1 −0.075 −0.05 −0.025 0 0.025 0.05 0.075
Beta Coefficient

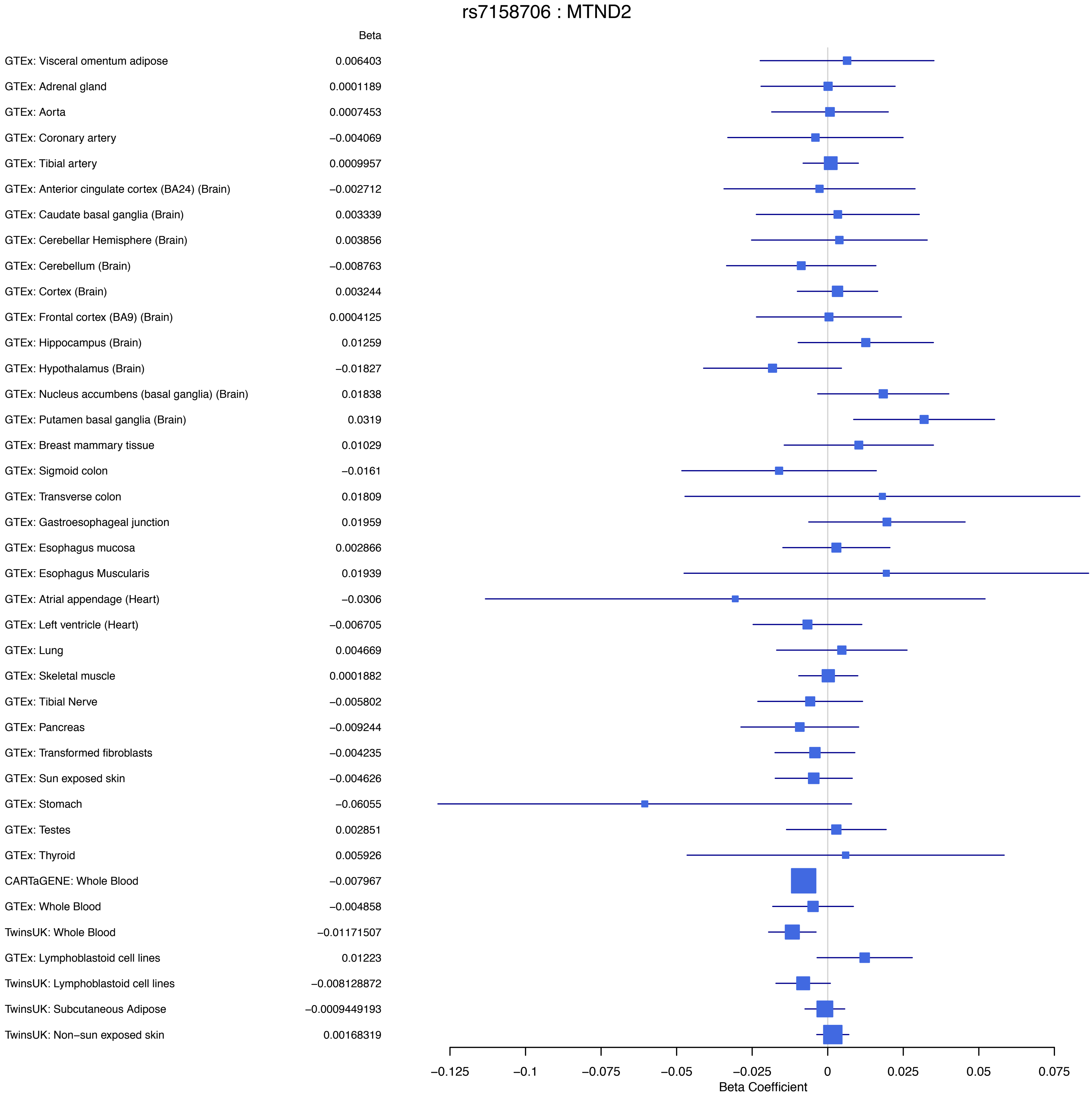
rs7158706 : MTND2
Beta
GTEx: Visceral omentum adipose 0.006403
GTEx: Adrenal gland 0.0001189
GTEx: Aorta 0.0007453
GTEx: Coronary artery −0.004069
GTEx: Tibial artery 0.0009957
GTEx: Anterior cingulate cortex (BA24) (Brain) −0.002712
GTEx: Caudate basal ganglia (Brain) 0.003339
GTEx: Cerebellar Hemisphere (Brain) 0.003856
GTEx: Cerebellum (Brain) −0.008763
GTEx: Cortex (Brain) 0.003244
GTEx: Frontal cortex (BA9) (Brain) 0.0004125
GTEx: Hippocampus (Brain) 0.01259
GTEx: Hypothalamus (Brain) −0.01827
GTEx: Nucleus accumbens (basal ganglia) (Brain) 0.01838
GTEx: Putamen basal ganglia (Brain) 0.0319
GTEx: Breast mammary tissue 0.01029
GTEx: Sigmoid colon −0.0161
GTEx: Transverse colon 0.01809
GTEx: Gastroesophageal junction 0.01959
GTEx: Esophagus mucosa 0.002866
GTEx: Esophagus Muscularis 0.01939
GTEx: Atrial appendage (Heart) −0.0306
GTEx: Left ventricle (Heart) −0.006705
GTEx: Lung 0.004669
GTEx: Skeletal muscle 0.0001882
GTEx: Tibial Nerve −0.005802
GTEx: Pancreas −0.009244
GTEx: Transformed fibroblasts −0.004235
GTEx: Sun exposed skin −0.004626
GTEx: Stomach −0.06055
GTEx: Testes 0.002851
GTEx: Thyroid 0.005926
CARTaGENE: Whole Blood −0.007967
GTEx: Whole Blood −0.004858
TwinsUK: Whole Blood −0.01171507
GTEx: Lymphoblastoid cell lines 0.01223
TwinsUK: Lymphoblastoid cell lines −0.008128872
TwinsUK: Subcutaneous Adipose −0.0009449193
TwinsUK: Non−sun exposed skin 0.00168319
−0.125
−0.1
−0.075
−0.05
−0.025
0
0.025
0.05
0.075
Beta Coefficient

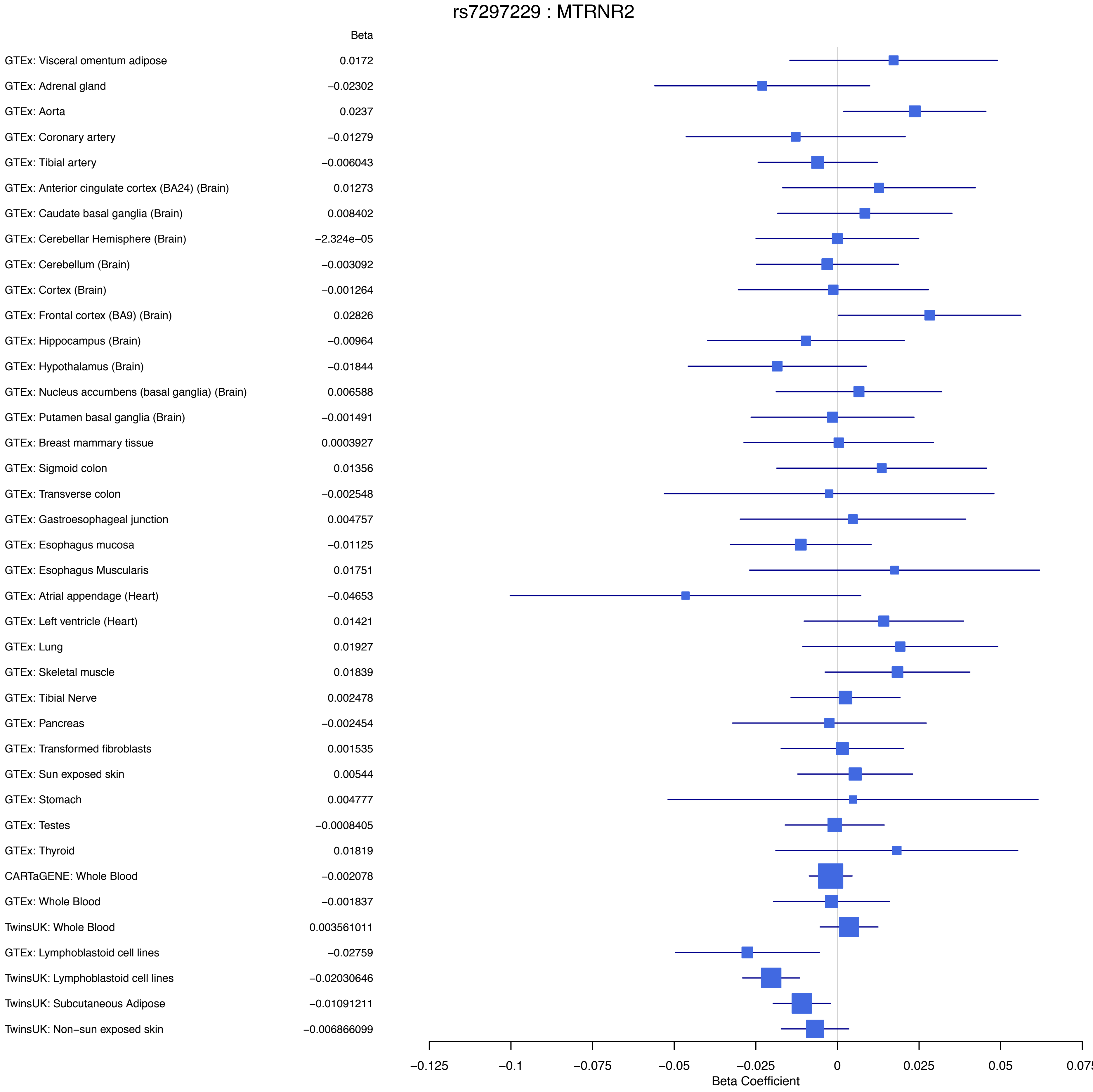
rs7297229 : MTRNR2
Beta
GTEx: Visceral omentum adipose 0.0172
GTEx: Adrenal gland −0.02302
GTEx: Aorta 0.0237
GTEx: Coronary artery −0.01279
GTEx: Tibial artery −0.006043
GTEx: Anterior cingulate cortex (BA24) (Brain) 0.01273
GTEx: Caudate basal ganglia (Brain) 0.008402
GTEx: Cerebellar Hemisphere (Brain) −2.324e−05
GTEx: Cerebellum (Brain) −0.003092
GTEx: Cortex (Brain) −0.001264
GTEx: Frontal cortex (BA9) (Brain) 0.02826
GTEx: Hippocampus (Brain) −0.00964
GTEx: Hypothalamus (Brain) −0.01844
GTEx: Nucleus accumbens (basal ganglia) (Brain) 0.006588
GTEx: Putamen basal ganglia (Brain) −0.001491
GTEx: Breast mammary tissue 0.0003927
GTEx: Sigmoid colon 0.01356
GTEx: Transverse colon −0.002548
GTEx: Gastroesophageal junction 0.004757
GTEx: Esophagus mucosa −0.01125
GTEx: Esophagus Muscularis 0.01751
GTEx: Atrial appendage (Heart) −0.04653
GTEx: Left ventricle (Heart) 0.01421
GTEx: Lung 0.01927
GTEx: Skeletal muscle 0.01839
GTEx: Tibial Nerve 0.002478
GTEx: Pancreas −0.002454
GTEx: Transformed fibroblasts 0.001535
GTEx: Sun exposed skin 0.00544
GTEx: Stomach 0.004777
GTEx: Testes −0.0008405
GTEx: Thyroid 0.01819
CARTaGENE: Whole Blood −0.002078
GTEx: Whole Blood −0.001837
TwinsUK: Whole Blood 0.003561011
GTEx: Lymphoblastoid cell lines −0.02759
TwinsUK: Lymphoblastoid cell lines −0.02030646
TwinsUK: Subcutaneous Adipose −0.01091211
TwinsUK: Non−sun exposed skin −0.006866099
−0.125 −0.1 −0.075 −0.05 −0.025 0 0.025 0.05 0.075
Beta Coefficient

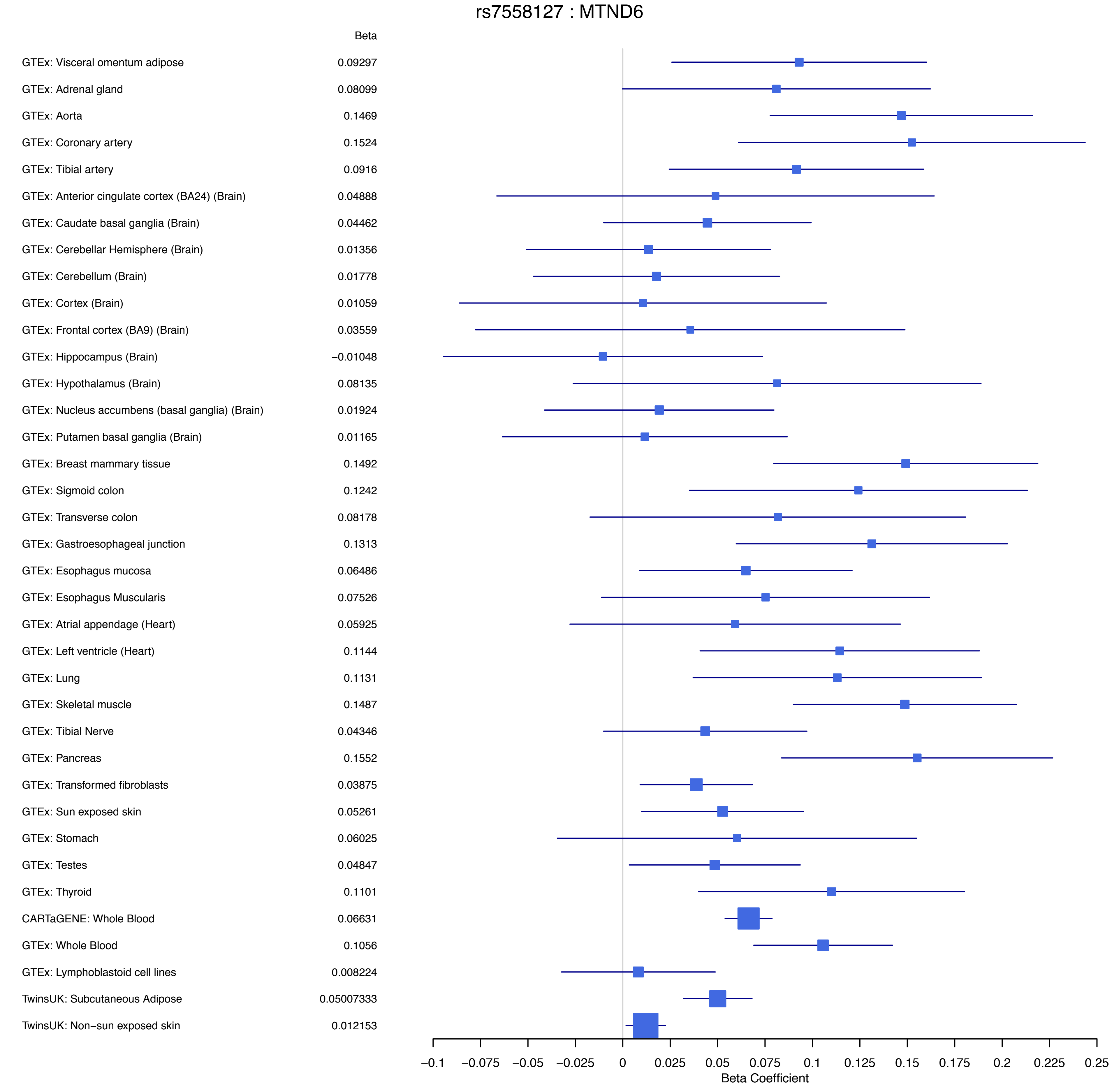
rs7558127 : MTND6
Beta
GTEx: Visceral omentum adipose 0.09297
GTEx: Adrenal gland 0.08099
GTEx: Aorta 0.1469
GTEx: Coronary artery 0.1524
GTEx: Tibial artery 0.0916
GTEx: Anterior cingulate cortex (BA24) (Brain) 0.04888
GTEx: Caudate basal ganglia (Brain) 0.04462
GTEx: Cerebellar Hemisphere (Brain) 0.01356
GTEx: Cerebellum (Brain) 0.01778
GTEx: Cortex (Brain) 0.01059
GTEx: Frontal cortex (BA9) (Brain) 0.03559
GTEx: Hippocampus (Brain) −0.01048
GTEx: Hypothalamus (Brain) 0.08135
GTEx: Nucleus accumbens (basal ganglia) (Brain) 0.01924
GTEx: Putamen basal ganglia (Brain) 0.01165
GTEx: Breast mammary tissue 0.1492
GTEx: Sigmoid colon 0.1242
GTEx: Transverse colon 0.08178
GTEx: Gastroesophageal junction 0.1313
GTEx: Esophagus mucosa 0.06486
GTEx: Esophagus Muscularis 0.07526
GTEx: Atrial appendage (Heart) 0.05925
GTEx: Left ventricle (Heart) 0.1144
GTEx: Lung 0.1131
GTEx: Skeletal muscle 0.1487
GTEx: Tibial Nerve 0.04346
GTEx: Pancreas 0.1552
GTEx: Transformed fibroblasts 0.03875
GTEx: Sun exposed skin 0.05261
GTEx: Stomach 0.06025
GTEx: Testes 0.04847
GTEx: Thyroid 0.1101
CARTaGENE: Whole Blood 0.06631
GTEx: Whole Blood 0.1056
GTEx: Lymphoblastoid cell lines 0.008224
TwinsUK: Subcutaneous Adipose 0.05007333
TwinsUK: Non−sun exposed skin 0.012153
−0.1
−0.075
−0.05
−0.025
0
0.025
0.05
0.075
0.1
0.125
0.15
0.175
0.2
0.225
0.25
Beta Coefficient

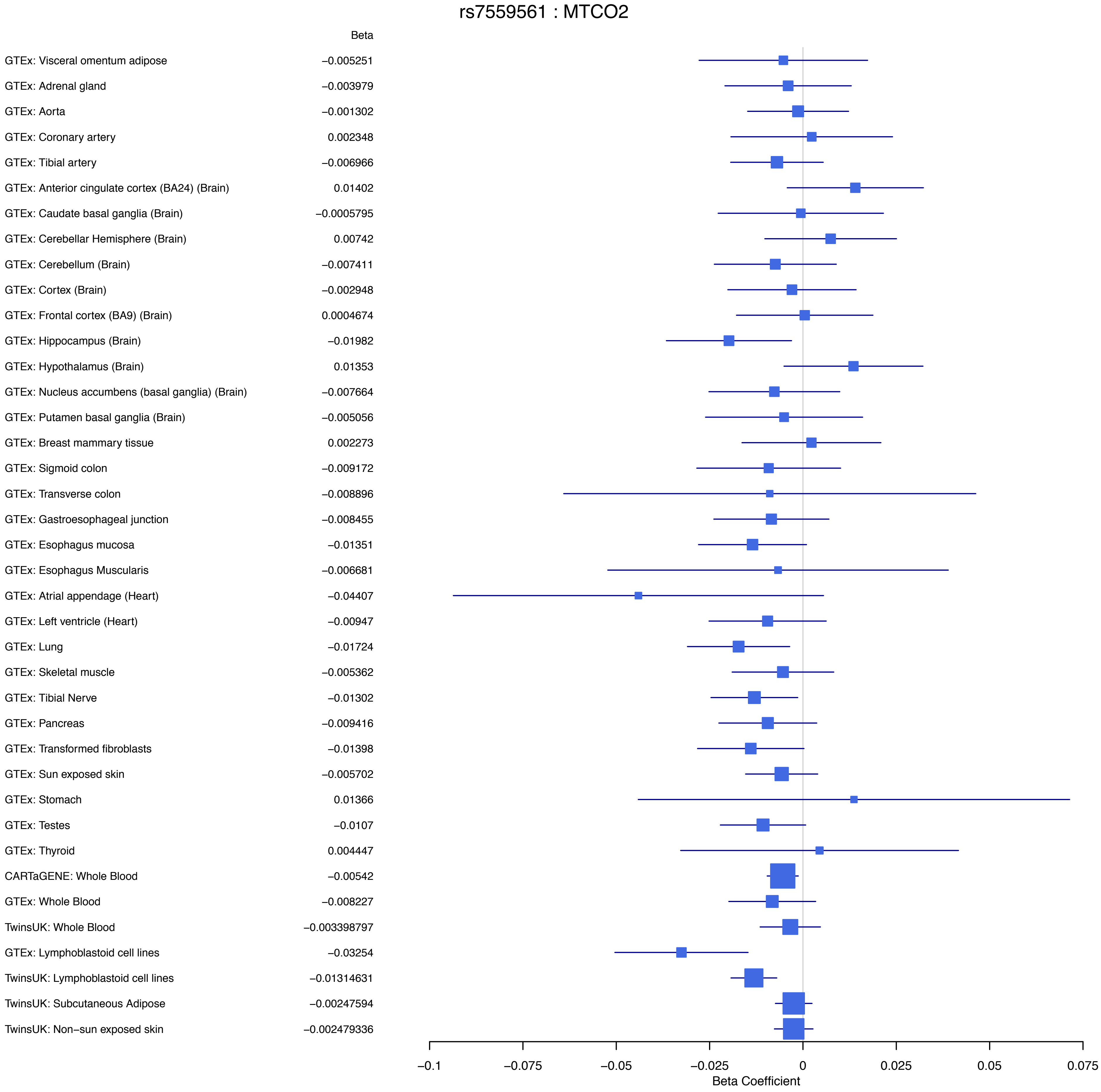
rs7559561 : MTCO2
Beta
GTEx: Visceral omentum adipose −0.005251
GTEx: Adrenal gland −0.003979
GTEx: Aorta −0.001302
GTEx: Coronary artery 0.002348
GTEx: Tibial artery −0.006966
GTEx: Anterior cingulate cortex (BA24) (Brain) 0.01402
GTEx: Caudate basal ganglia (Brain) −0.0005795
GTEx: Cerebellar Hemisphere (Brain) 0.00742
GTEx: Cerebellum (Brain) −0.007411
GTEx: Cortex (Brain) −0.002948
GTEx: Frontal cortex (BA9) (Brain) 0.0004674
GTEx: Hippocampus (Brain) −0.01982
GTEx: Hypothalamus (Brain) 0.01353
GTEx: Nucleus accumbens (basal ganglia) (Brain) −0.007664
GTEx: Putamen basal ganglia (Brain) −0.005056
GTEx: Breast mammary tissue 0.002273
GTEx: Sigmoid colon −0.009172
GTEx: Transverse colon −0.008896
GTEx: Gastroesophageal junction −0.008455
GTEx: Esophagus mucosa −0.01351
GTEx: Esophagus Muscularis −0.006681
GTEx: Atrial appendage (Heart) −0.04407
GTEx: Left ventricle (Heart) −0.00947
GTEx: Lung −0.01724
GTEx: Skeletal muscle −0.005362
GTEx: Tibial Nerve −0.01302
GTEx: Pancreas −0.009416
GTEx: Transformed fibroblasts −0.01398
GTEx: Sun exposed skin −0.005702
GTEx: Stomach 0.01366
GTEx: Testes −0.0107
GTEx: Thyroid 0.004447
CARTaGENE: Whole Blood −0.00542
GTEx: Whole Blood −0.008227
TwinsUK: Whole Blood −0.003398797
GTEx: Lymphoblastoid cell lines −0.03254
TwinsUK: Lymphoblastoid cell lines −0.01314631
TwinsUK: Subcutaneous Adipose −0.00247594
TwinsUK: Non−sun exposed skin −0.002479336
−0.1
−0.075
−0.05
−0.025
0
0.025
0.05
0.075
Beta Coefficient

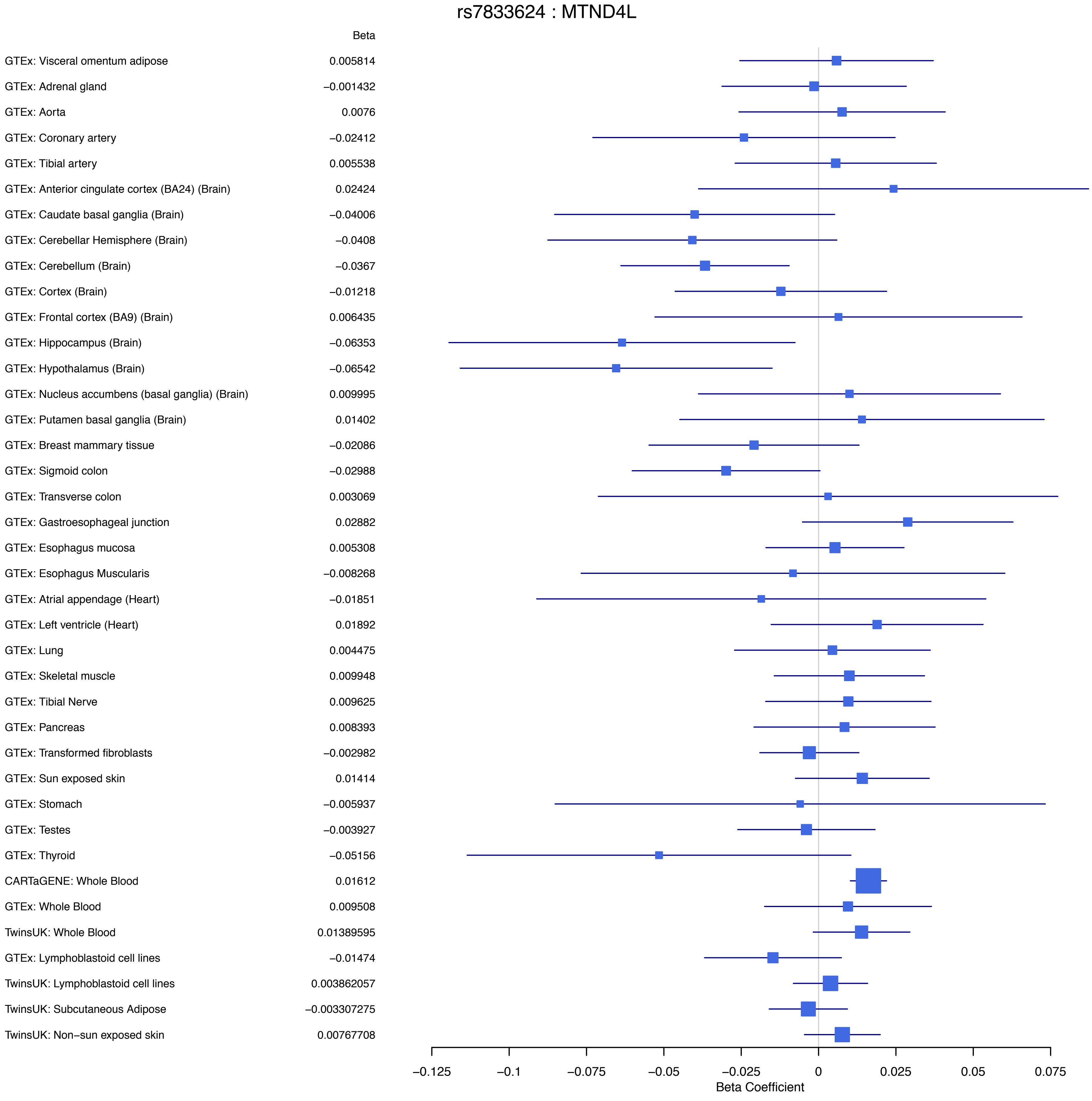
rs7833624 : MTND4L
Beta
GTEx: Visceral omentum adipose 0.005814
GTEx: Adrenal gland −0.001432
GTEx: Aorta 0.0076
GTEx: Coronary artery −0.02412
GTEx: Tibial artery 0.005538
GTEx: Anterior cingulate cortex (BA24) (Brain) 0.02424
GTEx: Caudate basal ganglia (Brain) −0.04006
GTEx: Cerebellar Hemisphere (Brain) −0.0408
GTEx: Cerebellum (Brain) −0.0367
GTEx: Cortex (Brain) −0.01218
GTEx: Frontal cortex (BA9) (Brain) 0.006435
GTEx: Hippocampus (Brain) −0.06353
GTEx: Hypothalamus (Brain) −0.06542
GTEx: Nucleus accumbens (basal ganglia) (Brain) 0.009995
GTEx: Putamen basal ganglia (Brain) 0.01402
GTEx: Breast mammary tissue −0.02086
GTEx: Sigmoid colon −0.02988
GTEx: Transverse colon 0.003069
GTEx: Gastroesophageal junction 0.02882
GTEx: Esophagus mucosa 0.005308
GTEx: Esophagus Muscularis −0.008268
GTEx: Atrial appendage (Heart) −0.01851
GTEx: Left ventricle (Heart) 0.01892
GTEx: Lung 0.004475
GTEx: Skeletal muscle 0.009948
GTEx: Tibial Nerve 0.009625
GTEx: Pancreas 0.008393
GTEx: Transformed fibroblasts −0.002982
GTEx: Sun exposed skin 0.01414
GTEx: Stomach −0.005937
GTEx: Testes −0.003927
GTEx: Thyroid −0.05156
CARTaGENE: Whole Blood 0.01612
GTEx: Whole Blood 0.009508
TwinsUK: Whole Blood 0.01389595
GTEx: Lymphoblastoid cell lines −0.01474
TwinsUK: Lymphoblastoid cell lines 0.003862057
TwinsUK: Subcutaneous Adipose −0.003307275
TwinsUK: Non−sun exposed skin 0.00767708
−0.125
−0.1
−0.075
−0.05
−0.025
0
0.025
0.05
0.075
Beta Coefficient

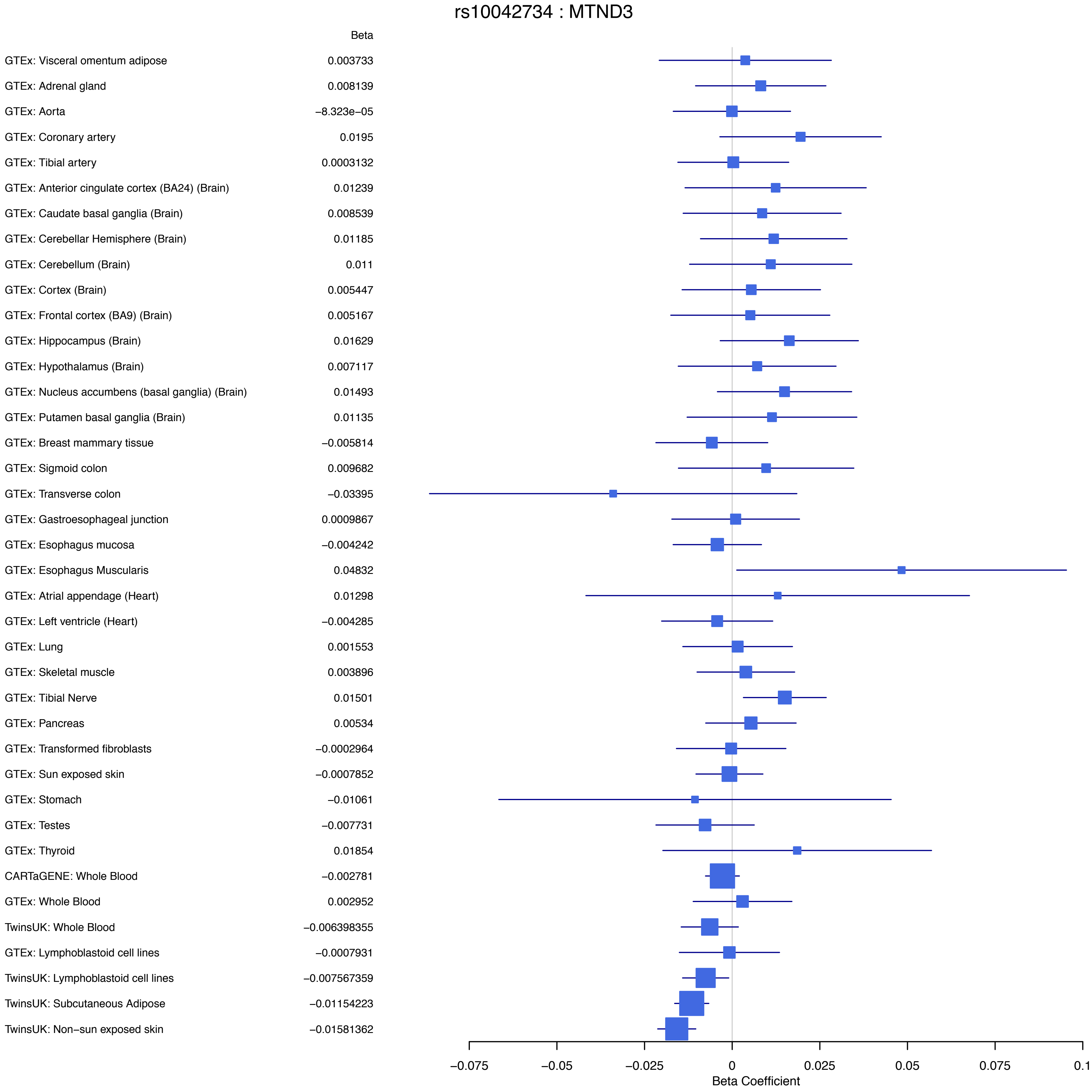
rs10042734 : MTND3
Beta
GTEx: Visceral omentum adipose 0.003733
GTEx: Adrenal gland 0.008139
GTEx: Aorta −8.323e−05
GTEx: Coronary artery 0.0195
GTEx: Tibial artery 0.0003132
GTEx: Anterior cingulate cortex (BA24) (Brain) 0.01239
GTEx: Caudate basal ganglia (Brain) 0.008539
GTEx: Cerebellar Hemisphere (Brain) 0.01185
GTEx: Cerebellum (Brain) 0.011
GTEx: Cortex (Brain) 0.005447
GTEx: Frontal cortex (BA9) (Brain) 0.005167
GTEx: Hippocampus (Brain) 0.01629
GTEx: Hypothalamus (Brain) 0.007117
GTEx: Nucleus accumbens (basal ganglia) (Brain) 0.01493
GTEx: Putamen basal ganglia (Brain) 0.01135
GTEx: Breast mammary tissue −0.005814
GTEx: Sigmoid colon 0.009682
GTEx: Transverse colon −0.03395
GTEx: Gastroesophageal junction 0.0009867
GTEx: Esophagus mucosa −0.004242
GTEx: Esophagus Muscularis 0.04832
GTEx: Atrial appendage (Heart) 0.01298
GTEx: Left ventricle (Heart) −0.004285
GTEx: Lung 0.001553
GTEx: Skeletal muscle 0.003896
GTEx: Tibial Nerve 0.01501
GTEx: Pancreas 0.00534
GTEx: Transformed fibroblasts −0.0002964
GTEx: Sun exposed skin −0.0007852
GTEx: Stomach −0.01061
GTEx: Testes −0.007731
GTEx: Thyroid 0.01854
CARTaGENE: Whole Blood −0.002781
GTEx: Whole Blood 0.002952
TwinsUK: Whole Blood −0.006398355
GTEx: Lymphoblastoid cell lines −0.0007931
TwinsUK: Lymphoblastoid cell lines −0.007567359
TwinsUK: Subcutaneous Adipose −0.01154223
TwinsUK: Non−sun exposed skin −0.01581362
−0.075
−0.05
−0.025
0
0.025
0.05
0.075
0.1
Beta Coefficient

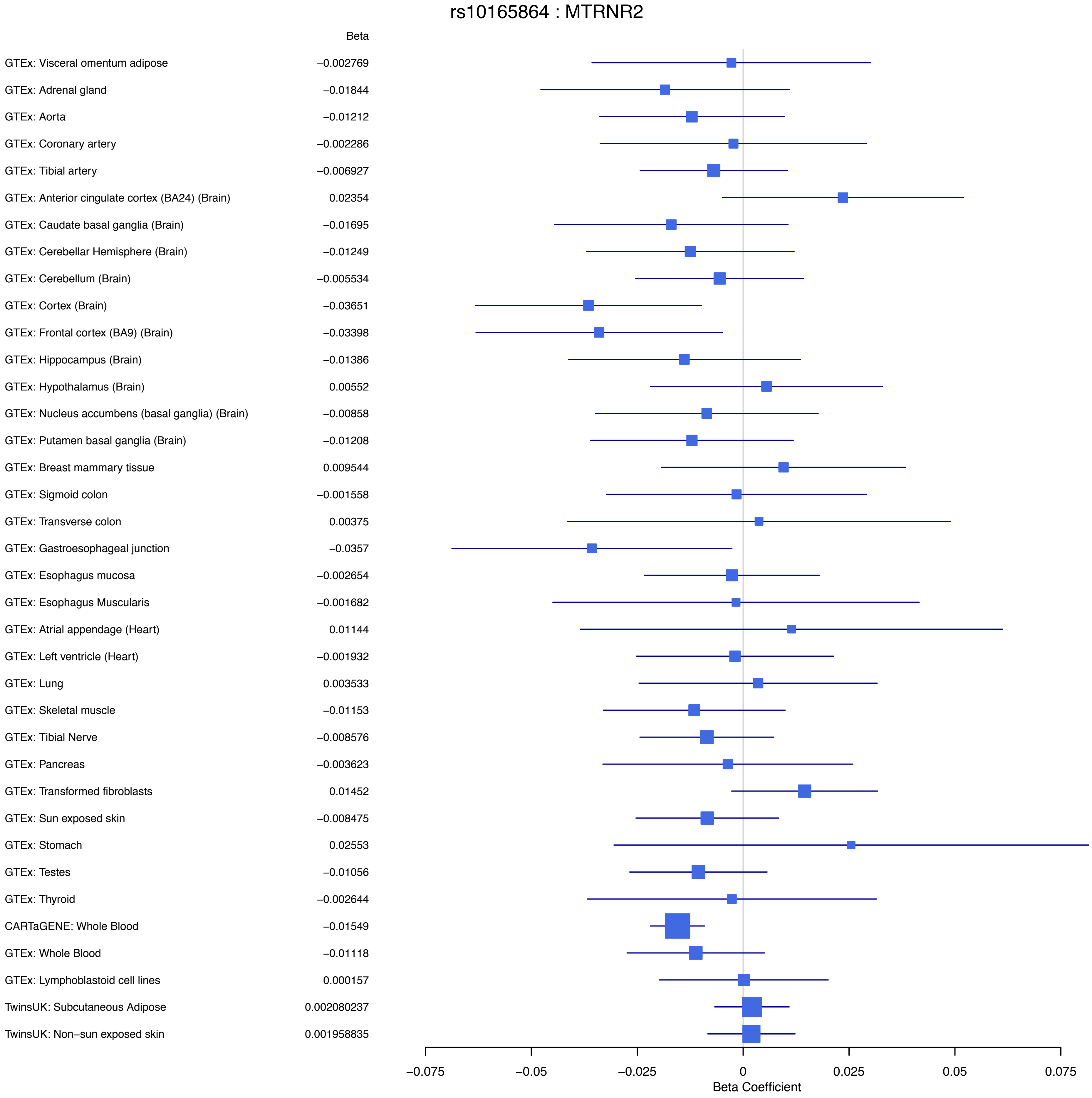

rs10165864 : MTRNR2
Beta
GTEx: Visceral omentum adipose
−0.002769
GTEx: Adrenal gland
−0.01844
GTEx: Aorta
−0.01212
GTEx: Coronary artery
−0.002286
GTEx: Tibial artery
−0.006927
GTEx: Anterior cingulate cortex (BA24) (Brain)
0.02354
GTEx: Caudate basal ganglia (Brain)
−0.01695
GTEx: Cerebellar Hemisphere (Brain)
−0.01249
GTEx: Cerebellum (Brain)
−0.005534
GTEx: Cortex (Brain)
−0.03651
GTEx: Frontal cortex (BA9) (Brain)
−0.03398
GTEx: Hippocampus (Brain)
−0.01386
GTEx: Hypothalamus (Brain)
0.00552
GTEx: Nucleus accumbens (basal ganglia) (Brain)
−0.00858
GTEx: Putamen basal ganglia (Brain)
−0.01208
GTEx: Breast mammary tissue
0.009544
GTEx: Sigmoid colon
−0.001558
GTEx: Transverse colon
0.00375
GTEx: Gastroesophageal junction
−0.0357
GTEx: Esophagus mucosa
−0.002654
GTEx: Esophagus Muscularis
−0.001682
GTEx: Atrial appendage (Heart)
0.01144
GTEx: Left ventricle (Heart)
−0.001932
GTEx: Lung
0.003533
GTEx: Skeletal muscle
−0.01153
GTEx: Tibial Nerve
−0.008576
GTEx: Pancreas
−0.003623
GTEx: Transformed fibroblasts
0.01452
GTEx: Sun exposed skin
−0.008475
GTEx: Stomach
0.02553
GTEx: Testes
−0.01056
GTEx: Thyroid
−0.002644
CARTaGENE: Whole Blood
−0.01549
GTEx: Whole Blood
−0.01118
GTEx: Lymphoblastoid cell lines
0.000157
TwinsUK: Subcutaneous Adipose
0.002080237
TwinsUK: Non−sun exposed skin
0.001958835
−0.075
−0.05
−0.025
0
0.025
0.05
0.075
Beta Coefficient

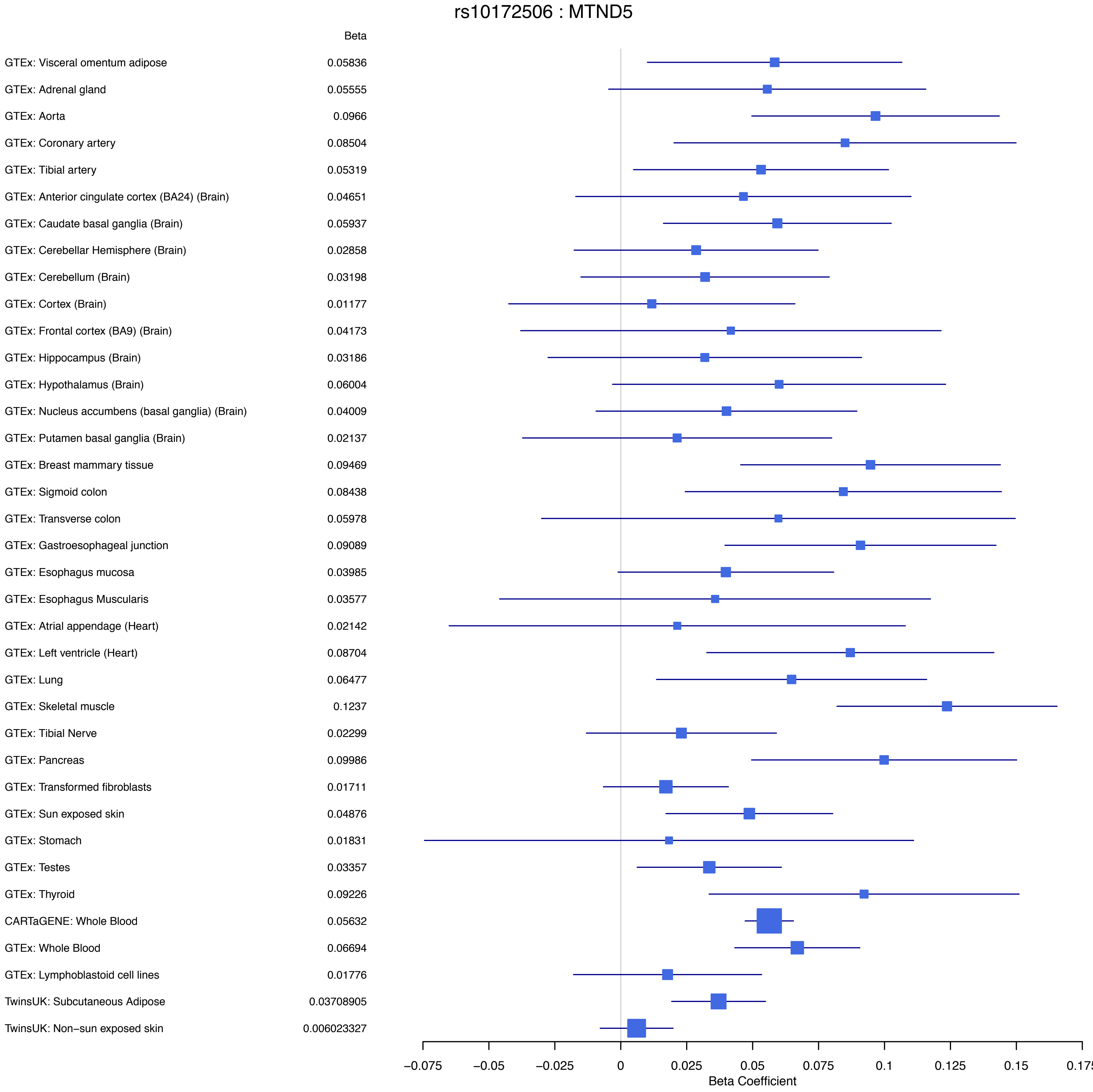
rs10172506 : MTND5
Beta
GTEx: Visceral omentum adipose 0.05836
GTEx: Adrenal gland 0.05555
GTEx: Aorta 0.0966
GTEx: Coronary artery 0.08504
GTEx: Tibial artery 0.05319
GTEx: Anterior cingulate cortex (BA24) (Brain) 0.04651
GTEx: Caudate basal ganglia (Brain) 0.05937
GTEx: Cerebellar Hemisphere (Brain) 0.02858
GTEx: Cerebellum (Brain) 0.03198
GTEx: Cortex (Brain) 0.01177
GTEx: Frontal cortex (BA9) (Brain) 0.04173
GTEx: Hippocampus (Brain) 0.03186
GTEx: Hypothalamus (Brain) 0.06004
GTEx: Nucleus accumbens (basal ganglia) (Brain) 0.04009
GTEx: Putamen basal ganglia (Brain) 0.02137
GTEx: Breast mammary tissue 0.09469
GTEx: Sigmoid colon 0.08438
GTEx: Transverse colon 0.05978
GTEx: Gastroesophageal junction 0.09089
GTEx: Esophagus mucosa 0.03985
GTEx: Esophagus Muscularis 0.03577
GTEx: Atrial appendage (Heart) 0.02142
GTEx: Left ventricle (Heart) 0.08704
GTEx: Lung 0.06477
GTEx: Skeletal muscle 0.1237
GTEx: Tibial Nerve 0.02299
GTEx: Pancreas 0.09986
GTEx: Transformed fibroblasts 0.01711
GTEx: Sun exposed skin 0.04876
GTEx: Stomach 0.01831
GTEx: Testes 0.03357
GTEx: Thyroid 0.09226
CARTaGENE: Whole Blood 0.05632
GTEx: Whole Blood 0.06694
GTEx: Lymphoblastoid cell lines 0.01776
TwinsUK: Subcutaneous Adipose 0.03708905
TwinsUK: Non−sun exposed skin 0.006023327
−0.075 −0.05 −0.025 0 0.025 0.05 0.075 0.1 0.125 0.15 0.175
Beta Coefficient

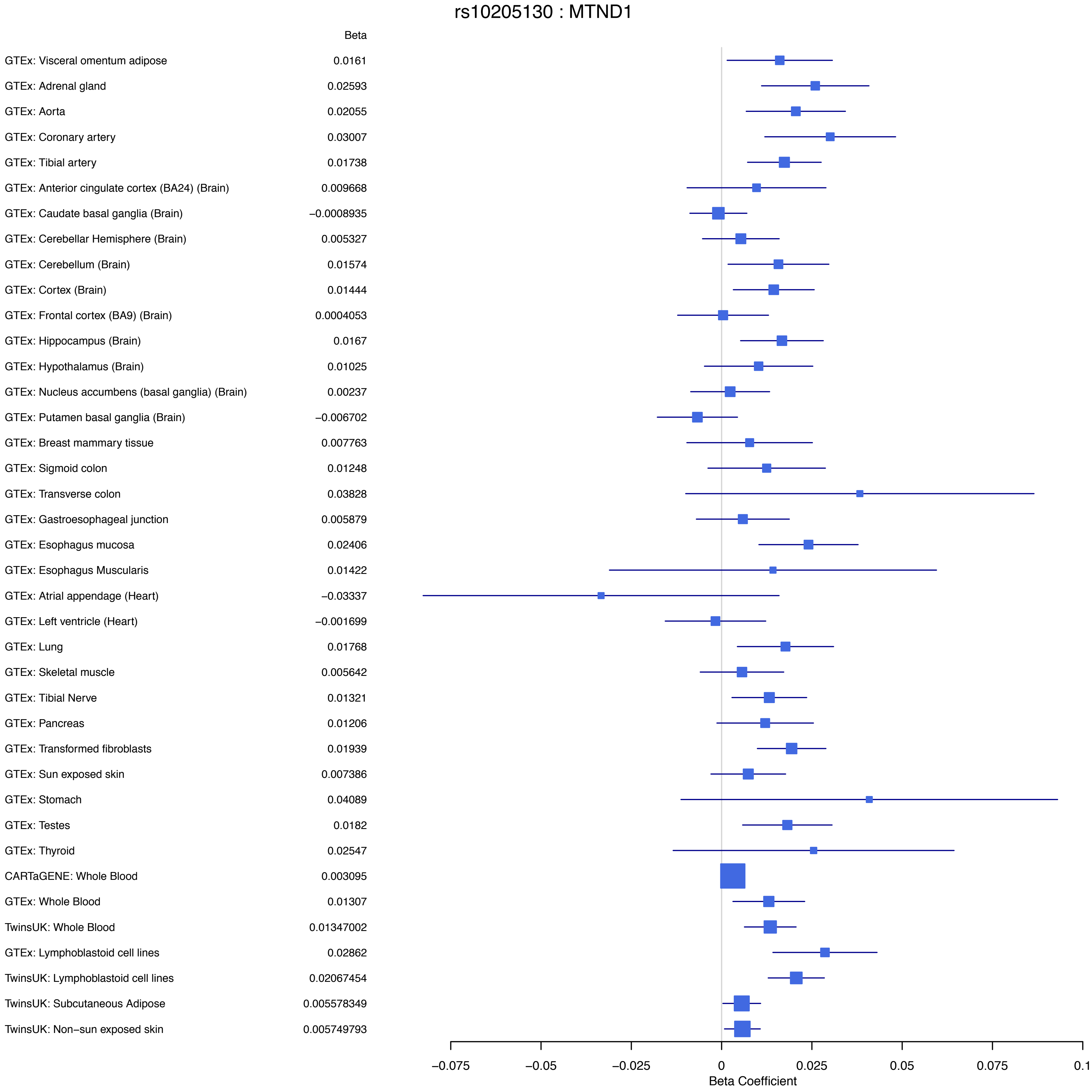
rs10205130 : MTND1
Beta
GTEx: Visceral omentum adipose 0.0161
GTEx: Adrenal gland 0.02593
GTEx: Aorta 0.02055
GTEx: Coronary artery 0.03007
GTEx: Tibial artery 0.01738
GTEx: Anterior cingulate cortex (BA24) (Brain) 0.009668
GTEx: Caudate basal ganglia (Brain) −0.0008935
GTEx: Cerebellar Hemisphere (Brain) 0.005327
GTEx: Cerebellum (Brain) 0.01574
GTEx: Cortex (Brain) 0.01444
GTEx: Frontal cortex (BA9) (Brain) 0.0004053
GTEx: Hippocampus (Brain) 0.0167
GTEx: Hypothalamus (Brain) 0.01025
GTEx: Nucleus accumbens (basal ganglia) (Brain) 0.00237
GTEx: Putamen basal ganglia (Brain) −0.006702
GTEx: Breast mammary tissue 0.007763
GTEx: Sigmoid colon 0.01248
GTEx: Transverse colon 0.03828
GTEx: Gastroesophageal junction 0.005879
GTEx: Esophagus mucosa 0.02406
GTEx: Esophagus Muscularis 0.01422
GTEx: Atrial appendage (Heart) −0.03337
GTEx: Left ventricle (Heart) −0.001699
GTEx: Lung 0.01768
GTEx: Skeletal muscle 0.005642
GTEx: Tibial Nerve 0.01321
GTEx: Pancreas 0.01206
GTEx: Transformed fibroblasts 0.01939
GTEx: Sun exposed skin 0.007386
GTEx: Stomach 0.04089
GTEx: Testes 0.0182
GTEx: Thyroid 0.02547
CARTaGENE: Whole Blood 0.003095
GTEx: Whole Blood 0.01307
TwinsUK: Whole Blood 0.01347002
GTEx: Lymphoblastoid cell lines 0.02862
TwinsUK: Lymphoblastoid cell lines 0.02067454
TwinsUK: Subcutaneous Adipose 0.005578349
TwinsUK: Non−sun exposed skin 0.005749793
−0.075
−0.05
−0.025
0
0.025
0.05
0.075
0.1
Beta Coefficient

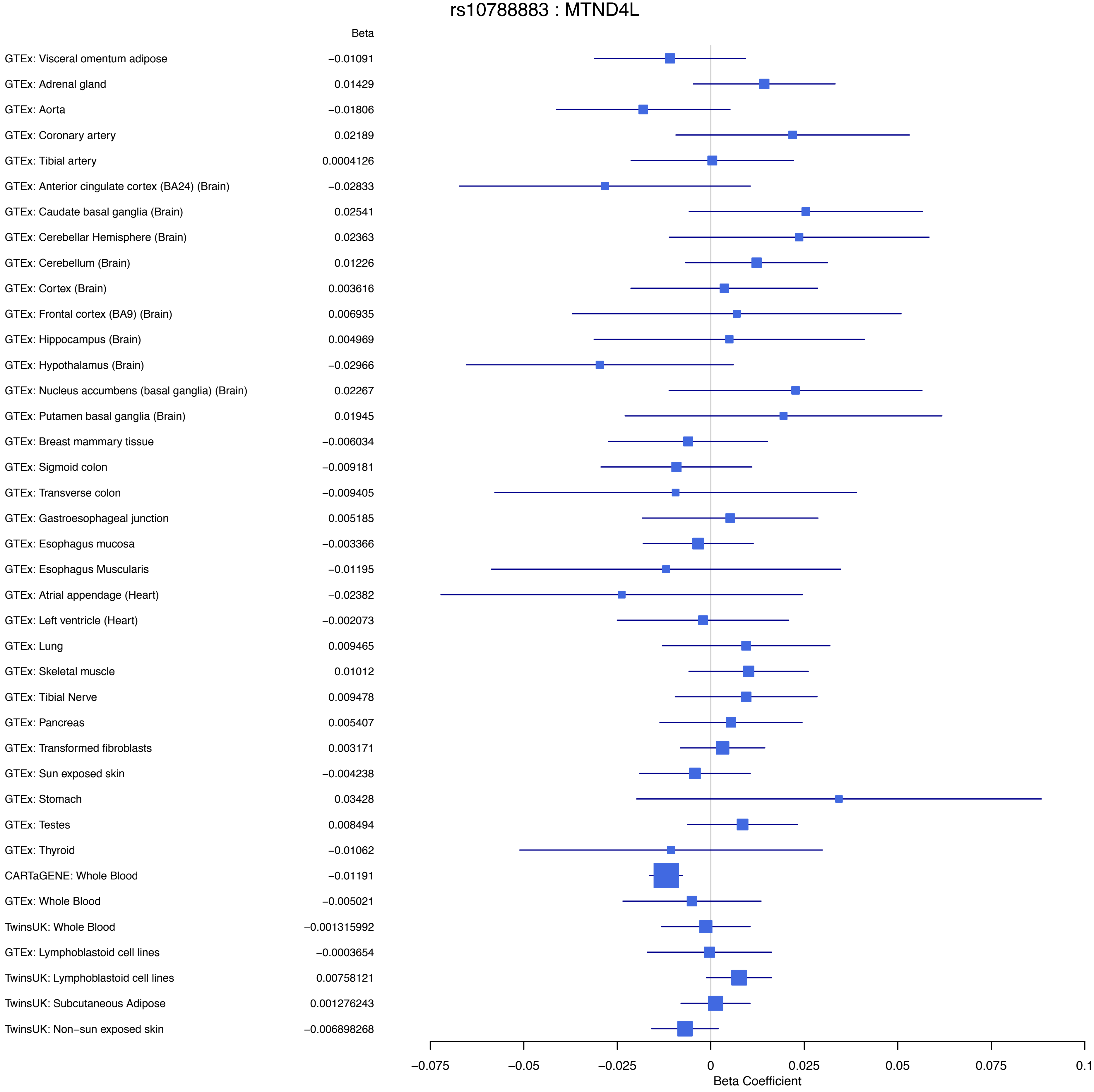
rs10788883 : MTND4L
Beta
GTEx: Visceral omentum adipose -0.01091
GTEx: Adrenal gland 0.01429
GTEx: Aorta -0.01806
GTEx: Coronary artery 0.02189
GTEx: Tibial artery 0.0004126
GTEx: Anterior cingulate cortex (BA24) (Brain) -0.02833
GTEx: Caudate basal ganglia (Brain) 0.02541
GTEx: Cerebellar Hemisphere (Brain) 0.02363
GTEx: Cerebellum (Brain) 0.01226
GTEx: Cortex (Brain) 0.003616
GTEx: Frontal cortex (BA9) (Brain) 0.006935
GTEx: Hippocampus (Brain) 0.004969
GTEx: Hypothalamus (Brain) -0.02966
GTEx: Nucleus accumbens (basal ganglia) (Brain) 0.02267
GTEx: Putamen basal ganglia (Brain) 0.01945
GTEx: Breast mammary tissue -0.006034
GTEx: Sigmoid colon -0.009181
GTEx: Transverse colon -0.009405
GTEx: Gastroesophageal junction 0.005185
GTEx: Esophagus mucosa -0.003366
GTEx: Esophagus Muscularis -0.01195
GTEx: Atrial appendage (Heart) -0.02382
GTEx: Left ventricle (Heart) -0.002073
GTEx: Lung 0.009465
GTEx: Skeletal muscle 0.01012
GTEx: Tibial Nerve 0.009478
GTEx: Pancreas 0.005407
GTEx: Transformed fibroblasts 0.003171
GTEx: Sun exposed skin -0.004238
GTEx: Stomach 0.03428
GTEx: Testes 0.008494
GTEx: Thyroid -0.01062
CARTaGENE: Whole Blood -0.01191
GTEx: Whole Blood -0.005021
TwinsUK: Whole Blood -0.001315992
GTEx: Lymphoblastoid cell lines -0.0003654
TwinsUK: Lymphoblastoid cell lines 0.00758121
TwinsUK: Subcutaneous Adipose 0.001276243
TwinsUK: Non-sun exposed skin -0.006898268
-0.075
-0.05
-0.025
0
0.025
0.05
0.075
0.1
Beta Coefficient

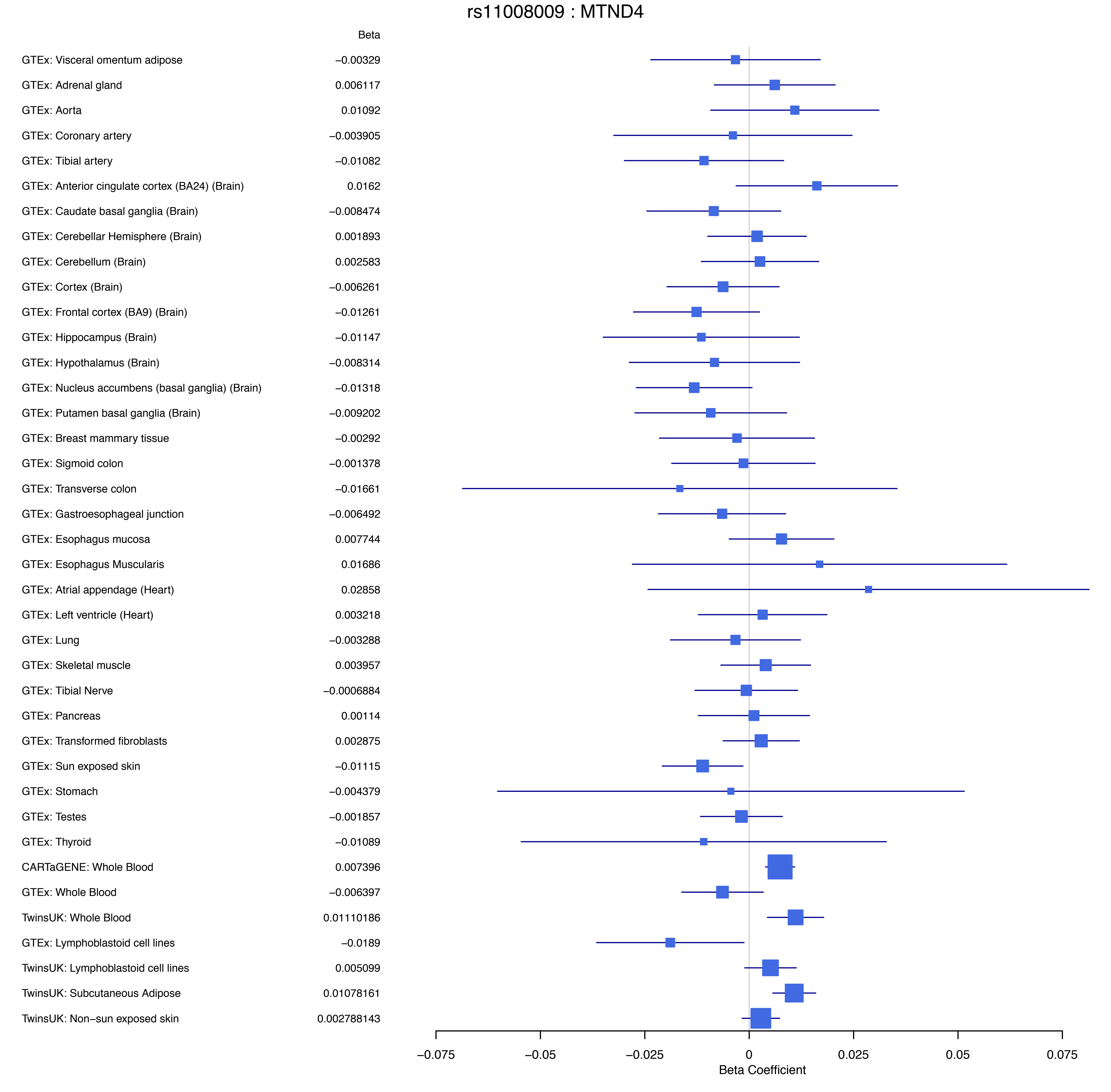
rs11008009 : MTND4
Beta
GTEx: Visceral omentum adipose −0.00329
GTEx: Adrenal gland 0.006117
GTEx: Aorta 0.01092
GTEx: Coronary artery −0.003905
GTEx: Tibial artery −0.01082
GTEx: Anterior cingulate cortex (BA24) (Brain) 0.0162
GTEx: Caudate basal ganglia (Brain) −0.008474
GTEx: Cerebellar Hemisphere (Brain) 0.001893
GTEx: Cerebellum (Brain) 0.002583
GTEx: Cortex (Brain) −0.006261
GTEx: Frontal cortex (BA9) (Brain) −0.01261
GTEx: Hippocampus (Brain) −0.01147
GTEx: Hypothalamus (Brain) −0.008314
GTEx: Nucleus accumbens (basal ganglia) (Brain) −0.01318
GTEx: Putamen basal ganglia (Brain) −0.009202
GTEx: Breast mammary tissue −0.00292
GTEx: Sigmoid colon −0.001378
GTEx: Transverse colon −0.01661
GTEx: Gastroesophageal junction −0.006492
GTEx: Esophagus mucosa 0.007744
GTEx: Esophagus Muscularis 0.01686
GTEx: Atrial appendage (Heart) 0.02858
GTEx: Left ventricle (Heart) 0.003218
GTEx: Lung −0.003288
GTEx: Skeletal muscle 0.003957
GTEx: Tibial Nerve −0.0006884
GTEx: Pancreas 0.00114
GTEx: Transformed fibroblasts 0.002875
GTEx: Sun exposed skin −0.01115
GTEx: Stomach −0.004379
GTEx: Testes −0.001857
GTEx: Thyroid −0.01089
CARTaGENE: Whole Blood 0.007396
GTEx: Whole Blood −0.006397
TwinsUK: Whole Blood 0.01110186
GTEx: Lymphoblastoid cell lines −0.0189
TwinsUK: Lymphoblastoid cell lines 0.005099
TwinsUK: Subcutaneous Adipose 0.01078161
TwinsUK: Non−sun exposed skin 0.002788143
−0.075
−0.05
−0.025
0
0.025
0.05
0.075
Beta Coefficient

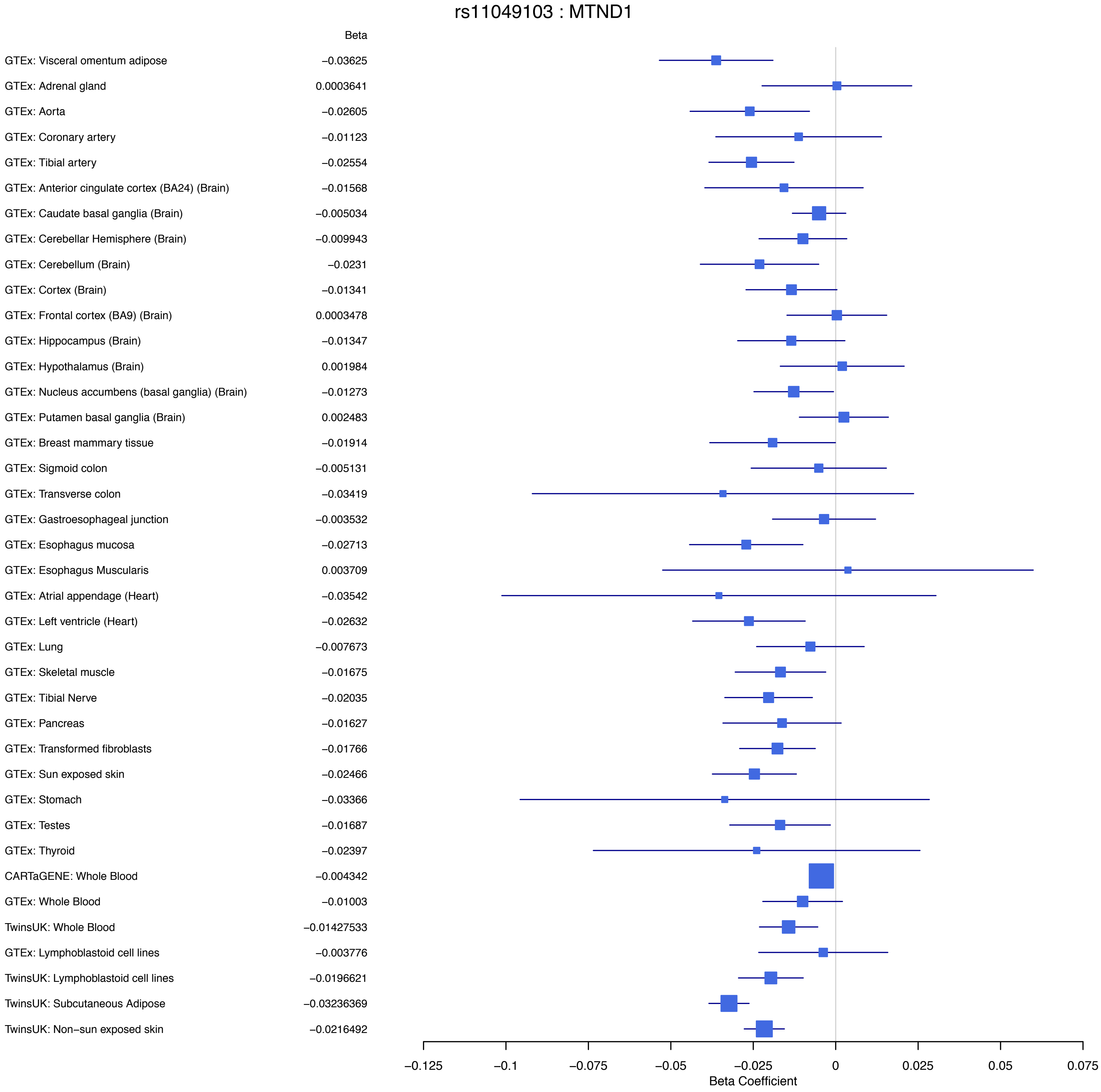
rs11049103 : MTND1
Beta
GTEx: Visceral omentum adipose −0.03625
GTEx: Adrenal gland 0.0003641
GTEx: Aorta −0.02605
GTEx: Coronary artery −0.01123
GTEx: Tibial artery −0.02554
GTEx: Anterior cingulate cortex (BA24) (Brain) −0.01568
GTEx: Caudate basal ganglia (Brain) −0.005034
GTEx: Cerebellar Hemisphere (Brain) −0.009943
GTEx: Cerebellum (Brain) −0.0231
GTEx: Cortex (Brain) −0.01341
GTEx: Frontal cortex (BA9) (Brain) 0.0003478
GTEx: Hippocampus (Brain) −0.01347
GTEx: Hypothalamus (Brain) 0.001984
GTEx: Nucleus accumbens (basal ganglia) (Brain) −0.01273
GTEx: Putamen basal ganglia (Brain) 0.002483
GTEx: Breast mammary tissue −0.01914
GTEx: Sigmoid colon −0.005131
GTEx: Transverse colon −0.03419
GTEx: Gastroesophageal junction −0.003532
GTEx: Esophagus mucosa −0.02713
GTEx: Esophagus Muscularis 0.003709
GTEx: Atrial appendage (Heart) −0.03542
GTEx: Left ventricle (Heart) −0.02632
GTEx: Lung −0.007673
GTEx: Skeletal muscle −0.01675
GTEx: Tibial Nerve −0.02035
GTEx: Pancreas −0.01627
GTEx: Transformed fibroblasts −0.01766
GTEx: Sun exposed skin −0.02466
GTEx: Stomach −0.03366
GTEx: Testes −0.01687
GTEx: Thyroid −0.02397
CARTaGENE: Whole Blood −0.004342
GTEx: Whole Blood −0.01003
TwinsUK: Whole Blood −0.01427533
GTEx: Lymphoblastoid cell lines −0.003776
TwinsUK: Lymphoblastoid cell lines −0.0196621
TwinsUK: Subcutaneous Adipose −0.03236369
TwinsUK: Non−sun exposed skin −0.0216492
−0.125
−0.1
−0.075
−0.05
−0.025
0
0.025
0.05
0.075
Beta Coefficient

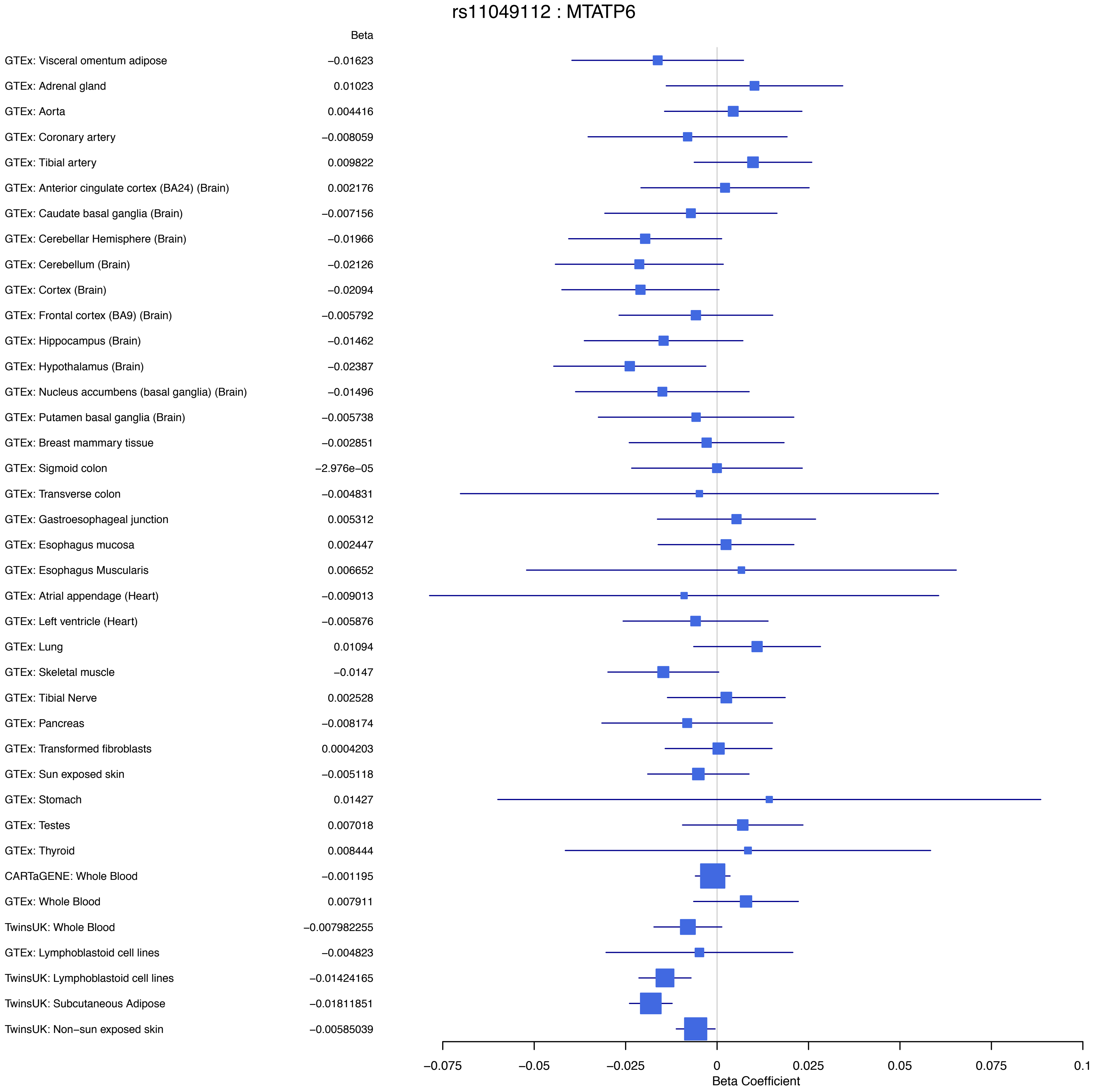
rs11049112 : MTATP6
Beta
GTEx: Visceral omentum adipose –0.01623
GTEx: Adrenal gland 0.01023
GTEx: Aorta 0.004416
GTEx: Coronary artery –0.008059
GTEx: Tibial artery 0.009822
GTEx: Anterior cingulate cortex (BA24) (Brain) 0.002176
GTEx: Caudate basal ganglia (Brain) –0.007156
GTEx: Cerebellar Hemisphere (Brain) –0.01966
GTEx: Cerebellum (Brain) –0.02126
GTEx: Cortex (Brain) –0.02094
GTEx: Frontal cortex (BA9) (Brain) –0.005792
GTEx: Hippocampus (Brain) –0.01462
GTEx: Hypothalamus (Brain) –0.02387
GTEx: Nucleus accumbens (basal ganglia) (Brain) –0.01496
GTEx: Putamen basal ganglia (Brain) –0.005738
GTEx: Breast mammary tissue –0.002851
GTEx: Sigmoid colon –2.976e–05
GTEx: Transverse colon –0.004831
GTEx: Gastroesophageal junction 0.005312
GTEx: Esophagus mucosa 0.002447
GTEx: Esophagus Muscularis 0.006652
GTEx: Atrial appendage (Heart) –0.009013
GTEx: Left ventricle (Heart) –0.005876
GTEx: Lung 0.01094
GTEx: Skeletal muscle –0.0147
GTEx: Tibial Nerve 0.002528
GTEx: Pancreas –0.008174
GTEx: Transformed fibroblasts 0.0004203
GTEx: Sun exposed skin –0.005118
GTEx: Stomach 0.01427
GTEx: Testes 0.007018
GTEx: Thyroid 0.008444
CARTaGENE: Whole Blood –0.001195
GTEx: Whole Blood 0.007911
TwinsUK: Whole Blood –0.007982255
GTEx: Lymphoblastoid cell lines –0.004823
TwinsUK: Lymphoblastoid cell lines –0.01424165
TwinsUK: Subcutaneous Adipose –0.01811851
TwinsUK: Non–sun exposed skin –0.00585039
–0.075
–0.05
–0.025
0
0.025
0.05
0.075
0.1
Beta Coefficient

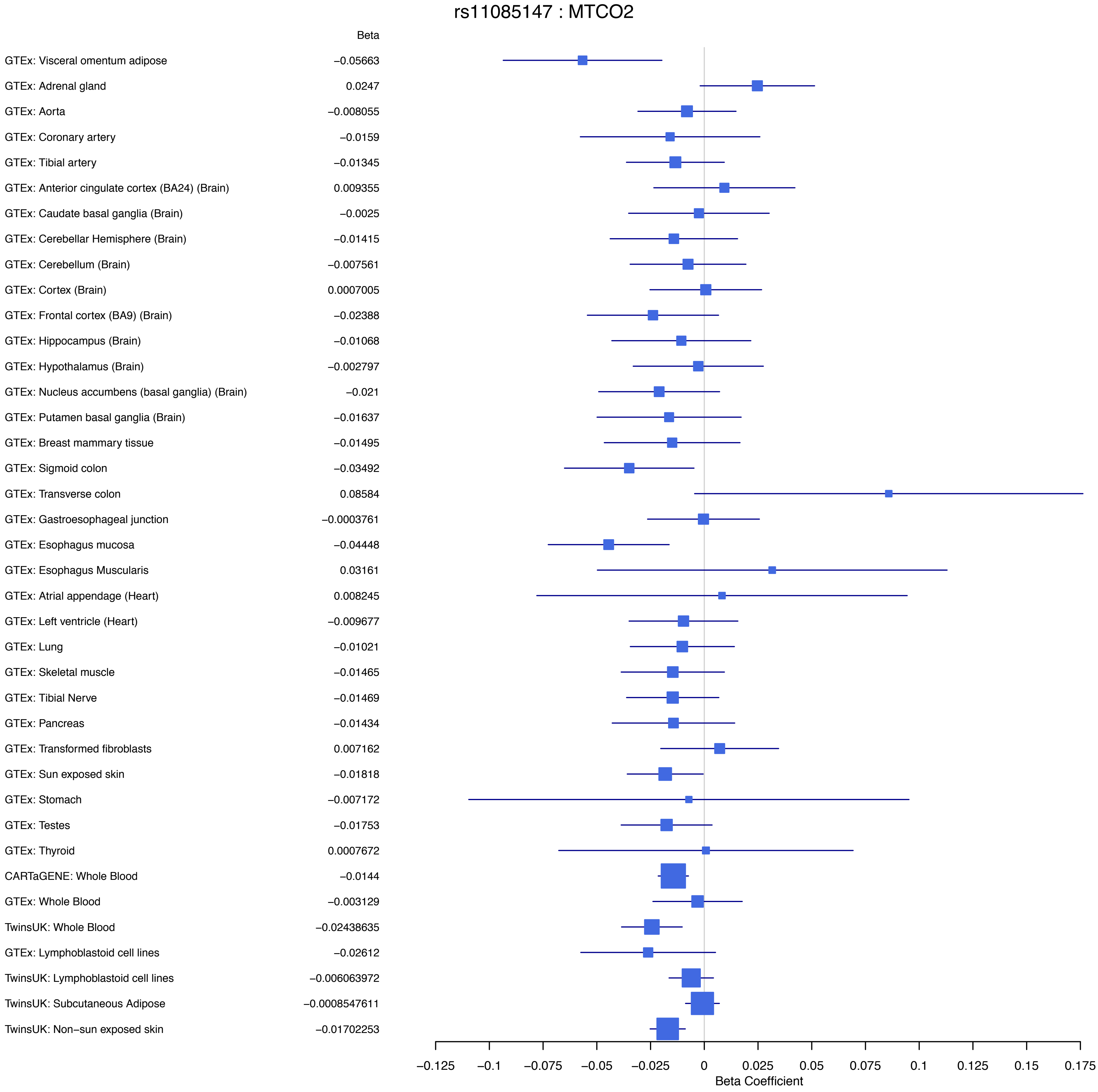
rs11085147 : MTCO2
Beta
GTEx: Visceral omentum adipose −0.05663
GTEx: Adrenal gland 0.0247
GTEx: Aorta −0.008055
GTEx: Coronary artery −0.0159
GTEx: Tibial artery −0.01345
GTEx: Anterior cingulate cortex (BA24) (Brain) 0.009355
GTEx: Caudate basal ganglia (Brain) −0.0025
GTEx: Cerebellar Hemisphere (Brain) −0.01415
GTEx: Cerebellum (Brain) −0.007561
GTEx: Cortex (Brain) 0.0007005
GTEx: Frontal cortex (BA9) (Brain) −0.02388
GTEx: Hippocampus (Brain) −0.01068
GTEx: Hypothalamus (Brain) −0.002797
GTEx: Nucleus accumbens (basal ganglia) (Brain) −0.021
GTEx: Putamen basal ganglia (Brain) −0.01637
GTEx: Breast mammary tissue −0.01495
GTEx: Sigmoid colon −0.03492
GTEx: Transverse colon 0.08584
GTEx: Gastroesophageal junction −0.0003761
GTEx: Esophagus mucosa −0.04448
GTEx: Esophagus Muscularis 0.03161
GTEx: Atrial appendage (Heart) 0.008245
GTEx: Left ventricle (Heart) −0.009677
GTEx: Lung −0.01021
GTEx: Skeletal muscle −0.01465
GTEx: Tibial Nerve −0.01469
GTEx: Pancreas −0.01434
GTEx: Transformed fibroblasts 0.007162
GTEx: Sun exposed skin −0.01818
GTEx: Stomach −0.007172
GTEx: Testes −0.01753
GTEx: Thyroid 0.0007672
CARTaGENE: Whole Blood −0.0144
GTEx: Whole Blood −0.003129
TwinsUK: Whole Blood −0.02438635
GTEx: Lymphoblastoid cell lines −0.02612
TwinsUK: Lymphoblastoid cell lines −0.006063972
TwinsUK: Subcutaneous Adipose −0.0008547611
TwinsUK: Non−sun exposed skin −0.01702253
−0.125 −0.1 −0.075 −0.05 −0.025 0 0.025 0.05 0.075 0.1 0.125 0.15 0.175
Beta Coefficient

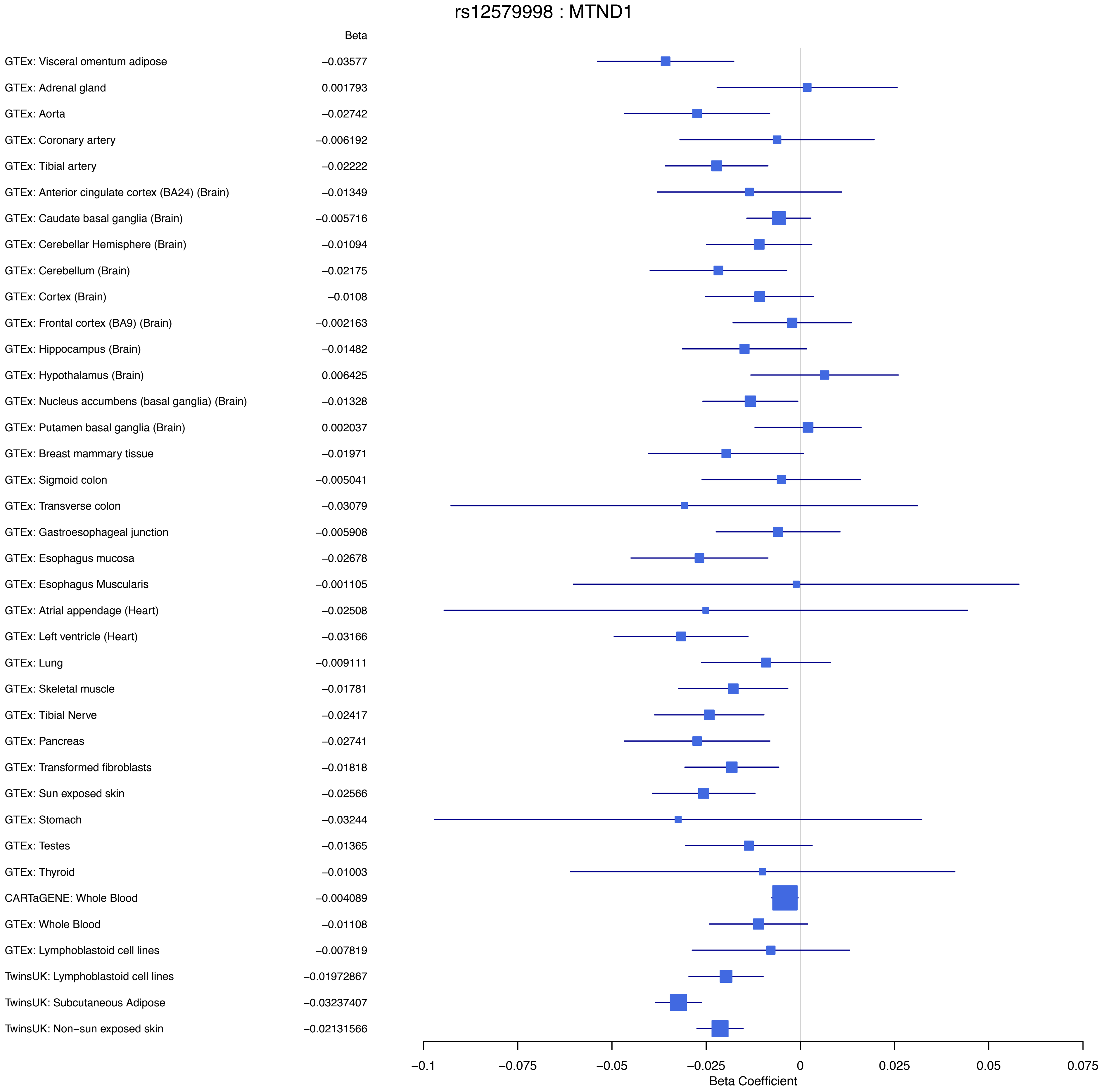
rs12579998 : MTND1
Beta
GTEx: Visceral omentum adipose –0.03577
GTEx: Adrenal gland 0.001793
GTEx: Aorta –0.02742
GTEx: Coronary artery –0.006192
GTEx: Tibial artery –0.02222
GTEx: Anterior cingulate cortex (BA24) (Brain) –0.01349
GTEx: Caudate basal ganglia (Brain) –0.005716
GTEx: Cerebellar Hemisphere (Brain) –0.01094
GTEx: Cerebellum (Brain) –0.02175
GTEx: Cortex (Brain) –0.0108
GTEx: Frontal cortex (BA9) (Brain) –0.002163
GTEx: Hippocampus (Brain) –0.01482
GTEx: Hypothalamus (Brain) 0.006425
GTEx: Nucleus accumbens (basal ganglia) (Brain) –0.01328
GTEx: Putamen basal ganglia (Brain) 0.002037
GTEx: Breast mammary tissue –0.01971
GTEx: Sigmoid colon –0.005041
GTEx: Transverse colon –0.03079
GTEx: Gastroesophageal junction –0.005908
GTEx: Esophagus mucosa –0.02678
GTEx: Esophagus Muscularis –0.001105
GTEx: Atrial appendage (Heart) –0.02508
GTEx: Left ventricle (Heart) –0.03166
GTEx: Lung –0.009111
GTEx: Skeletal muscle –0.01781
GTEx: Tibial Nerve –0.02417
GTEx: Pancreas –0.02741
GTEx: Transformed fibroblasts –0.01818
GTEx: Sun exposed skin –0.02566
GTEx: Stomach –0.03244
GTEx: Testes –0.01365
GTEx: Thyroid –0.01003
CARTaGENE: Whole Blood –0.004089
GTEx: Whole Blood –0.01108
GTEx: Lymphoblastoid cell lines –0.007819
TwinsUK: Lymphoblastoid cell lines –0.01972867
TwinsUK: Subcutaneous Adipose –0.03237407
TwinsUK: Non–sun exposed skin –0.02131566
–0.1
–0.075
–0.05
–0.025
0
0.025
0.05
0.075
Beta Coefficient

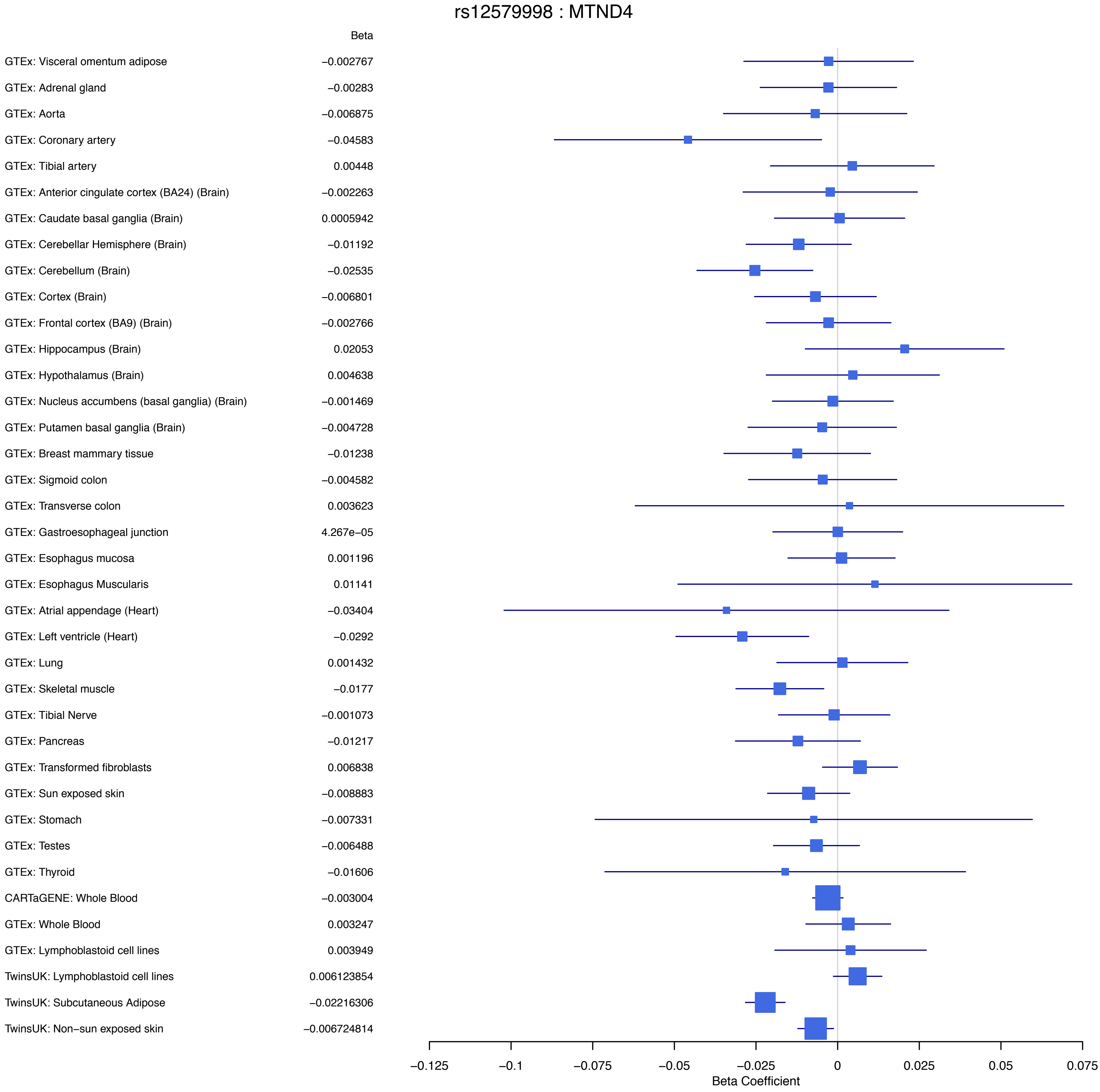
rs12579998 : MTND4
Beta
GTEx: Visceral omentum adipose −0.002767
GTEx: Adrenal gland −0.00283
GTEx: Aorta −0.006875
GTEx: Coronary artery −0.04583
GTEx: Tibial artery 0.00448
GTEx: Anterior cingulate cortex (BA24) (Brain) −0.002263
GTEx: Caudate basal ganglia (Brain) 0.0005942
GTEx: Cerebellar Hemisphere (Brain) −0.01192
GTEx: Cerebellum (Brain) −0.02535
GTEx: Cortex (Brain) −0.006801
GTEx: Frontal cortex (BA9) (Brain) −0.002766
GTEx: Hippocampus (Brain) 0.02053
GTEx: Hypothalamus (Brain) 0.004638
GTEx: Nucleus accumbens (basal ganglia) (Brain) −0.001469
GTEx: Putamen basal ganglia (Brain) −0.004728
GTEx: Breast mammary tissue −0.01238
GTEx: Sigmoid colon −0.004582
GTEx: Transverse colon 0.003623
GTEx: Gastroesophageal junction 4.267e−05
GTEx: Esophagus mucosa 0.001196
GTEx: Esophagus Muscularis 0.01141
GTEx: Atrial appendage (Heart) −0.03404
GTEx: Left ventricle (Heart) −0.0292
GTEx: Lung 0.001432
GTEx: Skeletal muscle −0.0177
GTEx: Tibial Nerve −0.001073
GTEx: Pancreas −0.01217
GTEx: Transformed fibroblasts 0.006838
GTEx: Sun exposed skin −0.008883
GTEx: Stomach −0.007331
GTEx: Testes −0.006488
GTEx: Thyroid −0.01606
CARTaGENE: Whole Blood −0.003004
GTEx: Whole Blood 0.003247
GTEx: Lymphoblastoid cell lines 0.003949
TwinsUK: Lymphoblastoid cell lines 0.006123854
TwinsUK: Subcutaneous Adipose −0.02216306
TwinsUK: Non−sun exposed skin −0.006724814
−0.125
−0.1
−0.075
−0.05
−0.025
0
0.025
0.05
0.075
Beta Coefficient

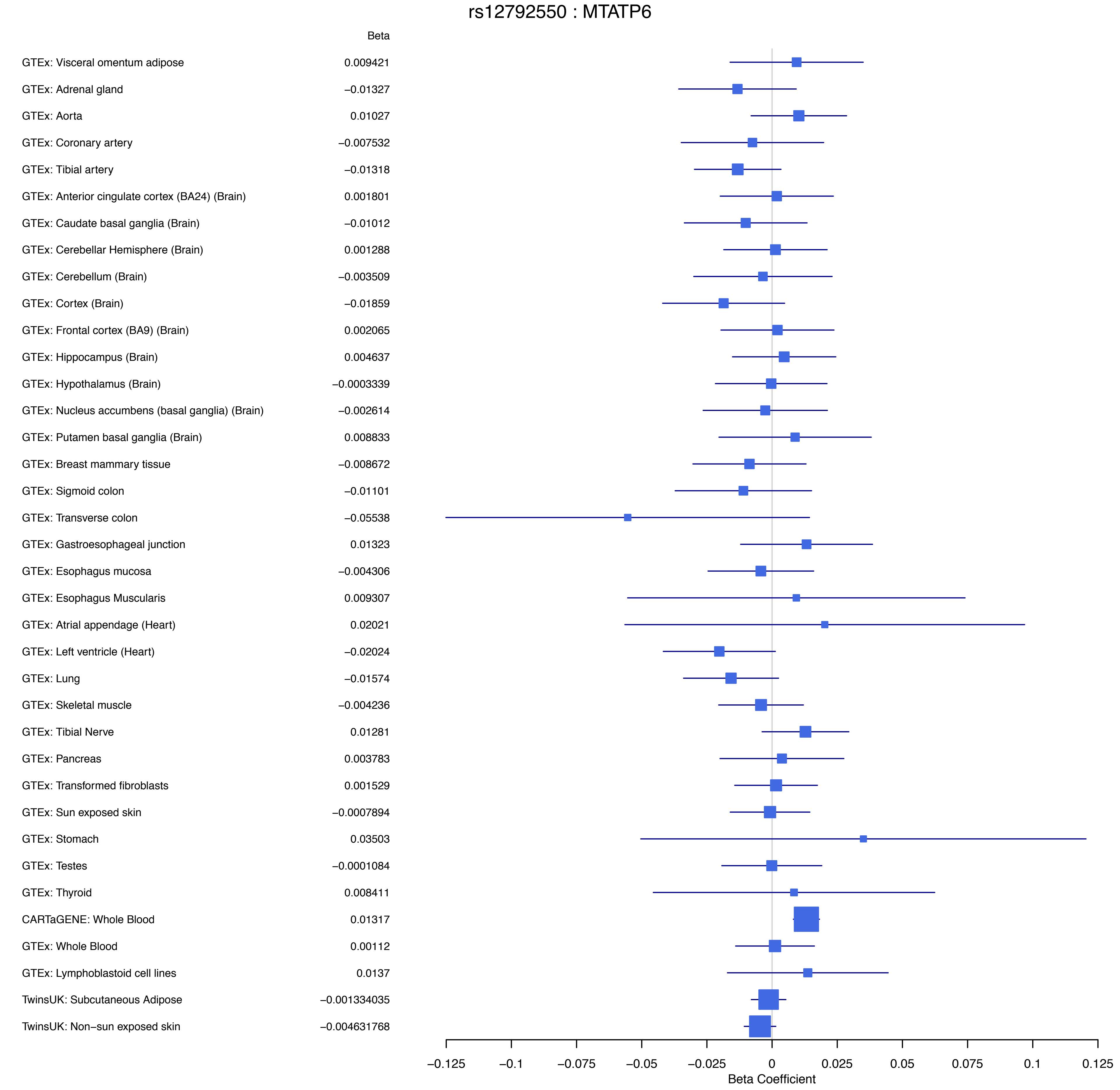
rs12792550 : MTATP6
Beta
GTEx: Visceral omentum adipose 0.009421
GTEx: Adrenal gland −0.01327
GTEx: Aorta 0.01027
GTEx: Coronary artery −0.007532
GTEx: Tibial artery −0.01318
GTEx: Anterior cingulate cortex (BA24) (Brain) 0.001801
GTEx: Caudate basal ganglia (Brain) −0.01012
GTEx: Cerebellar Hemisphere (Brain) 0.001288
GTEx: Cerebellum (Brain) −0.003509
GTEx: Cortex (Brain) −0.01859
GTEx: Frontal cortex (BA9) (Brain) 0.002065
GTEx: Hippocampus (Brain) 0.004637
GTEx: Hypothalamus (Brain) −0.0003339
GTEx: Nucleus accumbens (basal ganglia) (Brain) −0.002614
GTEx: Putamen basal ganglia (Brain) 0.008833
GTEx: Breast mammary tissue −0.008672
GTEx: Sigmoid colon −0.01101
GTEx: Transverse colon −0.05538
GTEx: Gastroesophageal junction 0.01323
GTEx: Esophagus mucosa −0.004306
GTEx: Esophagus Muscularis 0.009307
GTEx: Atrial appendage (Heart) 0.02021
GTEx: Left ventricle (Heart) −0.02024
GTEx: Lung −0.01574
GTEx: Skeletal muscle −0.004236
GTEx: Tibial Nerve 0.01281
GTEx: Pancreas 0.003783
GTEx: Transformed fibroblasts 0.001529
GTEx: Sun exposed skin −0.0007894
GTEx: Stomach 0.03503
GTEx: Testes −0.0001084
GTEx: Thyroid 0.008411
CARTaGENE: Whole Blood 0.01317
GTEx: Whole Blood 0.00112
GTEx: Lymphoblastoid cell lines 0.0137
TwinsUK: Subcutaneous Adipose −0.001334035
TwinsUK: Non−sun exposed skin −0.004631768
−0.125 −0.1 −0.075 −0.05 −0.025 0 0.025 0.05 0.075 0.1 0.125
Beta Coefficient

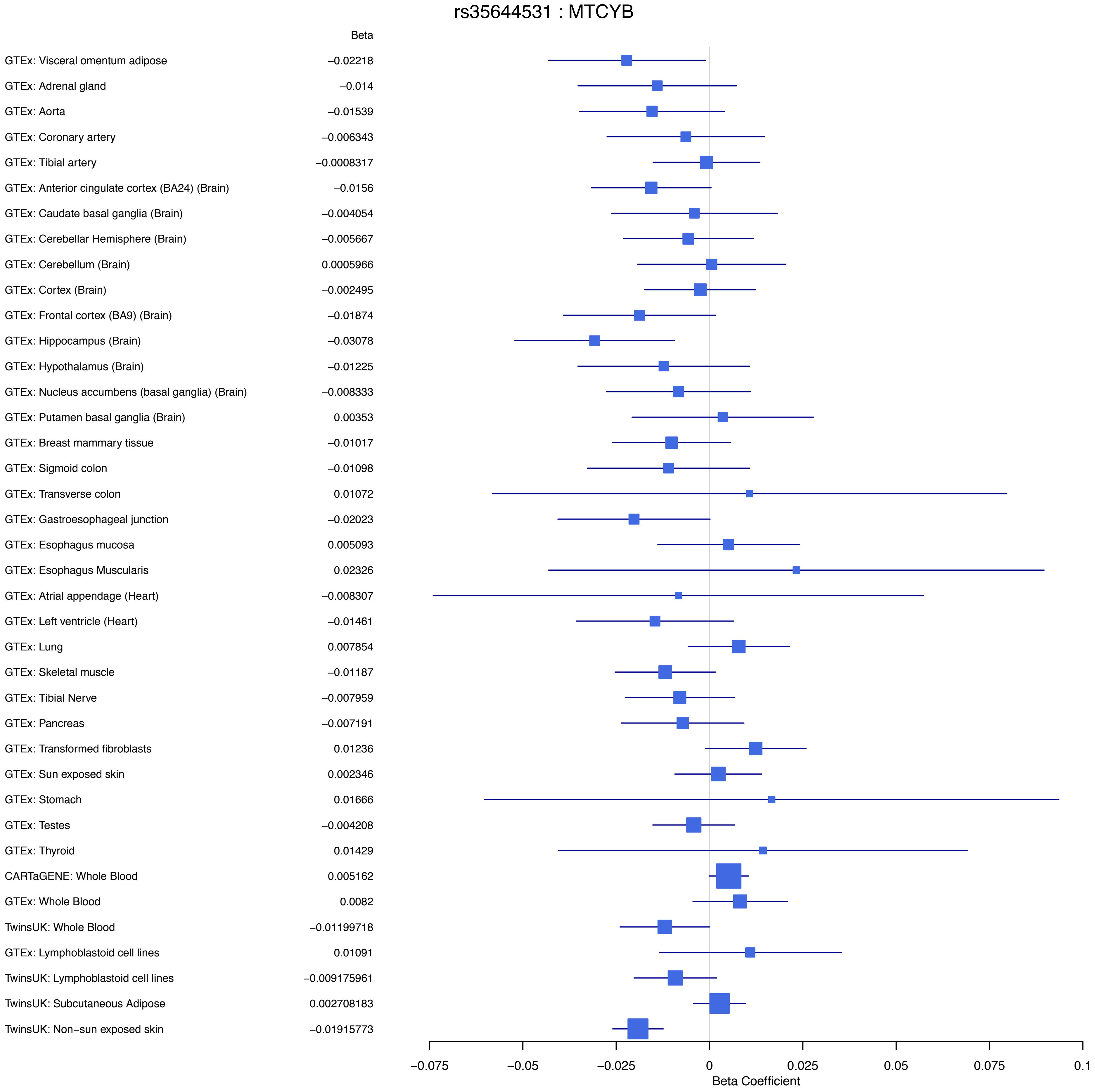
rs35644531 : MTCYB
Beta
GTEx: Visceral omentum adipose −0.02218
GTEx: Adrenal gland −0.014
GTEx: Aorta −0.01539
GTEx: Coronary artery −0.006343
GTEx: Tibial artery −0.0008317
GTEx: Anterior cingulate cortex (BA24) (Brain) −0.0156
GTEx: Caudate basal ganglia (Brain) −0.004054
GTEx: Cerebellar Hemisphere (Brain) −0.005667
GTEx: Cerebellum (Brain) 0.0005966
GTEx: Cortex (Brain) −0.002495
GTEx: Frontal cortex (BA9) (Brain) −0.01874
GTEx: Hippocampus (Brain) −0.03078
GTEx: Hypothalamus (Brain) −0.01225
GTEx: Nucleus accumbens (basal ganglia) (Brain) −0.008333
GTEx: Putamen basal ganglia (Brain) 0.00353
GTEx: Breast mammary tissue −0.01017
GTEx: Sigmoid colon −0.01098
GTEx: Transverse colon 0.01072
GTEx: Gastroesophageal junction −0.02023
GTEx: Esophagus mucosa 0.005093
GTEx: Esophagus Muscularis 0.02326
GTEx: Atrial appendage (Heart) −0.008307
GTEx: Left ventricle (Heart) −0.01461
GTEx: Lung 0.007854
GTEx: Skeletal muscle −0.01187
GTEx: Tibial Nerve −0.007959
GTEx: Pancreas −0.007191
GTEx: Transformed fibroblasts 0.01236
GTEx: Sun exposed skin 0.002346
GTEx: Stomach 0.01666
GTEx: Testes −0.004208
GTEx: Thyroid 0.01429
CARTaGENE: Whole Blood 0.005162
GTEx: Whole Blood 0.0082
TwinsUK: Whole Blood −0.01199718
GTEx: Lymphoblastoid cell lines 0.01091
TwinsUK: Lymphoblastoid cell lines −0.009175961
TwinsUK: Subcutaneous Adipose 0.002708183
TwinsUK: Non−sun exposed skin −0.01915773
−0.075
−0.05
−0.025
0
0.025
0.05
0.075
0.1
Beta Coefficient

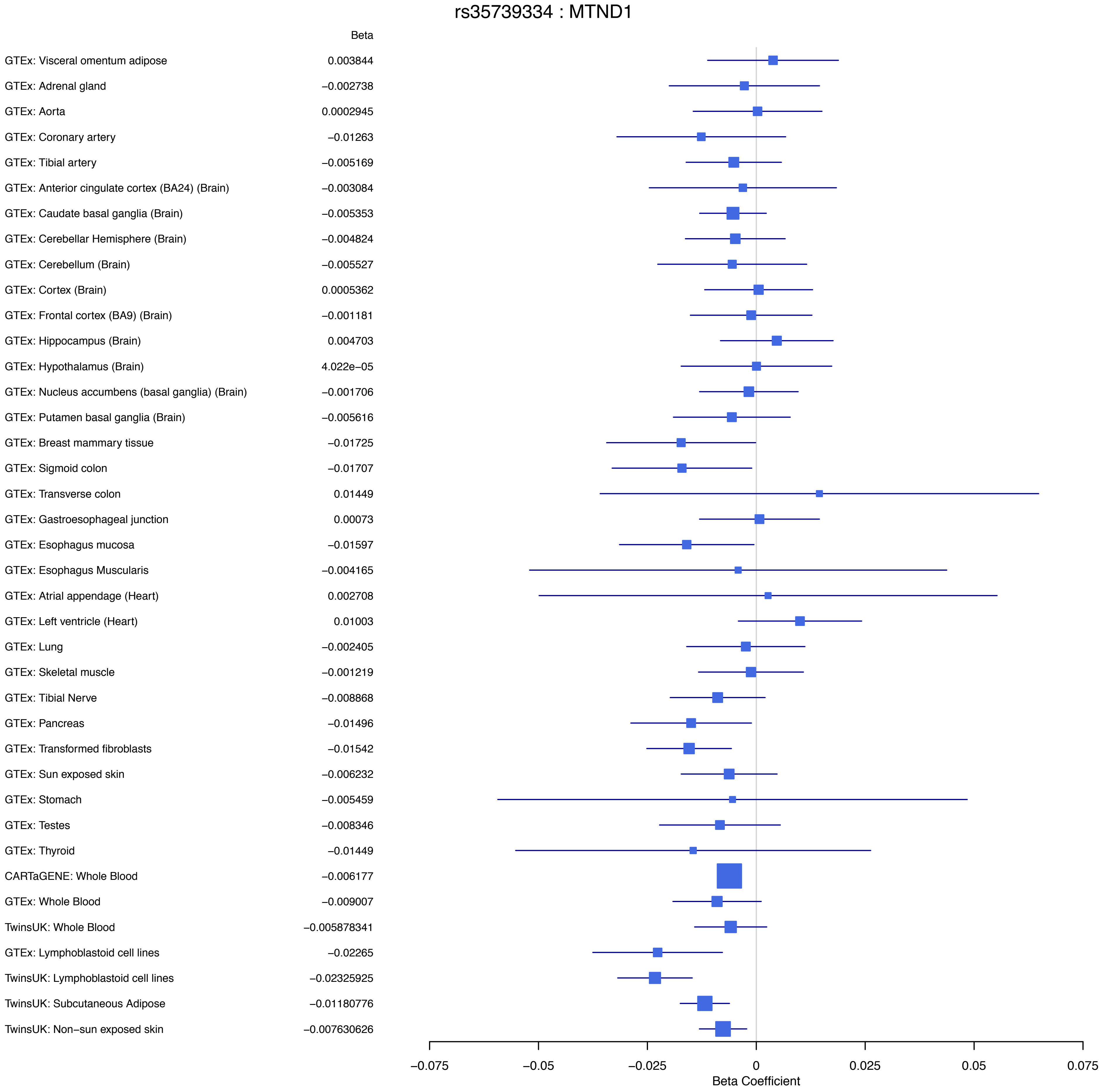

rs35739334 : MTND1
Beta
GTEx: Visceral omentum adipose 0.003844
GTEx: Adrenal gland −0.002738
GTEx: Aorta 0.0002945
GTEx: Coronary artery −0.01263
GTEx: Tibial artery −0.005169
GTEx: Anterior cingulate cortex (BA24) (Brain) −0.003084
GTEx: Caudate basal ganglia (Brain) −0.005353
GTEx: Cerebellar Hemisphere (Brain) −0.004824
GTEx: Cerebellum (Brain) −0.005527
GTEx: Cortex (Brain) 0.0005362
GTEx: Frontal cortex (BA9) (Brain) −0.001181
GTEx: Hippocampus (Brain) 0.004703
GTEx: Hypothalamus (Brain) 4.022e−05
GTEx: Nucleus accumbens (basal ganglia) (Brain) −0.001706
GTEx: Putamen basal ganglia (Brain) −0.005616
GTEx: Breast mammary tissue −0.01725
GTEx: Sigmoid colon −0.01707
GTEx: Transverse colon 0.01449
GTEx: Gastroesophageal junction 0.00073
GTEx: Esophagus mucosa −0.01597
GTEx: Esophagus Muscularis −0.004165
GTEx: Atrial appendage (Heart) 0.002708
GTEx: Left ventricle (Heart) 0.01003
GTEx: Lung −0.002405
GTEx: Skeletal muscle −0.001219
GTEx: Tibial Nerve −0.008868
GTEx: Pancreas −0.01496
GTEx: Transformed fibroblasts −0.01542
GTEx: Sun exposed skin −0.006232
GTEx: Stomach −0.005459
GTEx: Testes −0.008346
GTEx: Thyroid −0.01449
CARTaGENE: Whole Blood −0.006177
GTEx: Whole Blood −0.009007
TwinsUK: Whole Blood −0.005878341
GTEx: Lymphoblastoid cell lines −0.02265
TwinsUK: Lymphoblastoid cell lines −0.02325925
TwinsUK: Subcutaneous Adipose −0.01180776
TwinsUK: Non−sun exposed skin −0.007630626
−0.075
−0.05
−0.025
0
0.025
0.05
0.075
Beta Coefficient

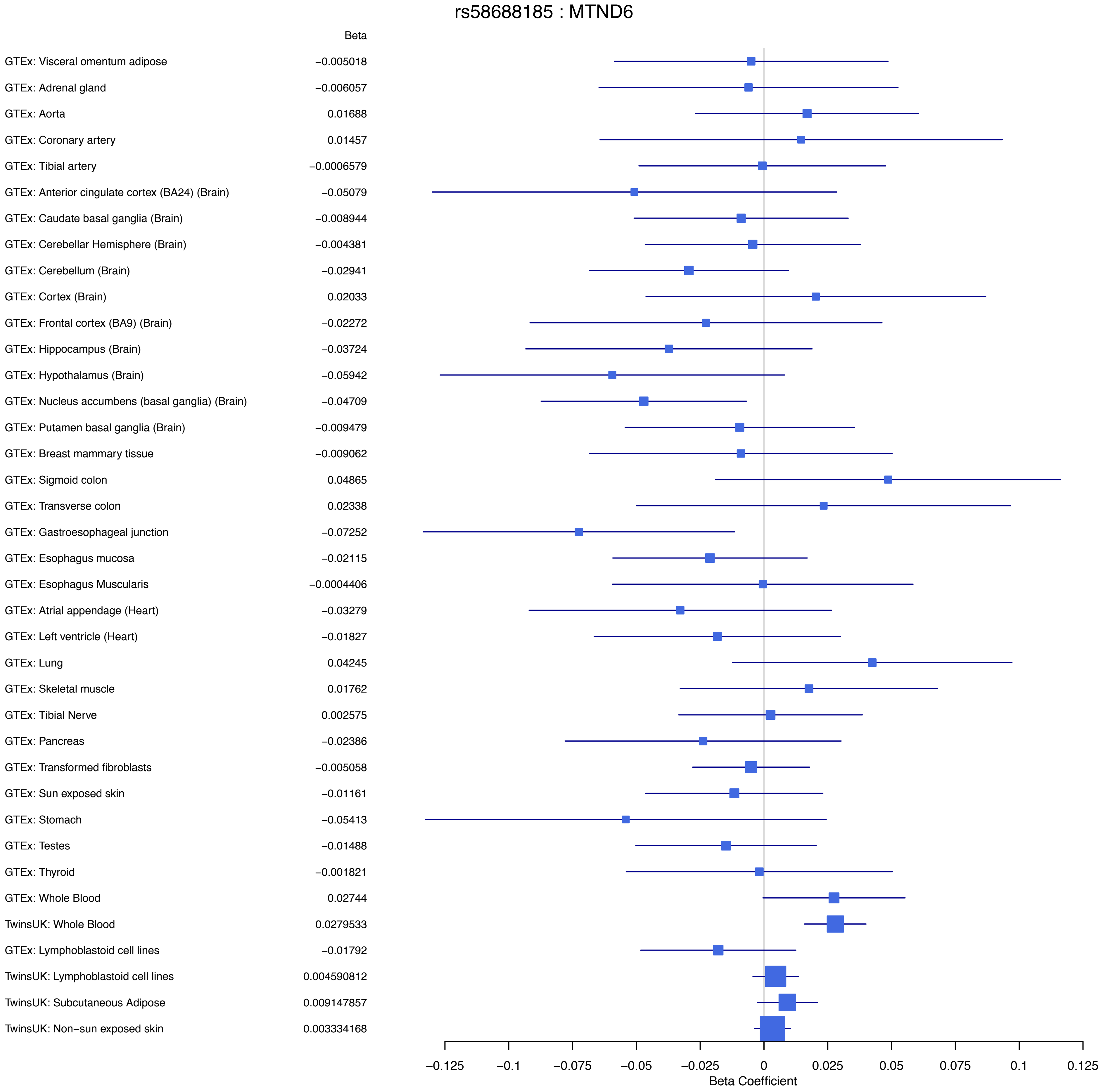
rs58688185 : MTND6
Beta
GTEx: Visceral omentum adipose −0.005018
GTEx: Adrenal gland −0.006057
GTEx: Aorta 0.01688
GTEx: Coronary artery 0.01457
GTEx: Tibial artery −0.0006579
GTEx: Anterior cingulate cortex (BA24) (Brain) −0.05079
GTEx: Caudate basal ganglia (Brain) −0.008944
GTEx: Cerebellar Hemisphere (Brain) −0.004381
GTEx: Cerebellum (Brain) −0.02941
GTEx: Cortex (Brain) 0.02033
GTEx: Frontal cortex (BA9) (Brain) −0.02272
GTEx: Hippocampus (Brain) −0.03724
GTEx: Hypothalamus (Brain) −0.05942
GTEx: Nucleus accumbens (basal ganglia) (Brain) −0.04709
GTEx: Putamen basal ganglia (Brain) −0.009479
GTEx: Breast mammary tissue −0.009062
GTEx: Sigmoid colon 0.04865
GTEx: Transverse colon 0.02338
GTEx: Gastroesophageal junction −0.07252
GTEx: Esophagus mucosa −0.02115
GTEx: Esophagus Muscularis −0.0004406
GTEx: Atrial appendage (Heart) −0.03279
GTEx: Left ventricle (Heart) −0.01827
GTEx: Lung 0.04245
GTEx: Skeletal muscle 0.01762
GTEx: Tibial Nerve 0.002575
GTEx: Pancreas −0.02386
GTEx: Transformed fibroblasts −0.005058
GTEx: Sun exposed skin −0.01161
GTEx: Stomach −0.05413
GTEx: Testes −0.01488
GTEx: Thyroid −0.001821
GTEx: Whole Blood 0.02744
TwinsUK: Whole Blood 0.0279533
GTEx: Lymphoblastoid cell lines −0.01792
TwinsUK: Lymphoblastoid cell lines 0.004590812
TwinsUK: Subcutaneous Adipose 0.009147857
TwinsUK: Non−sun exposed skin 0.003334168
−0.125
−0.1
−0.075
−0.05
−0.025
0
0.025
0.05
0.075
0.1
0.125
Beta Coefficient

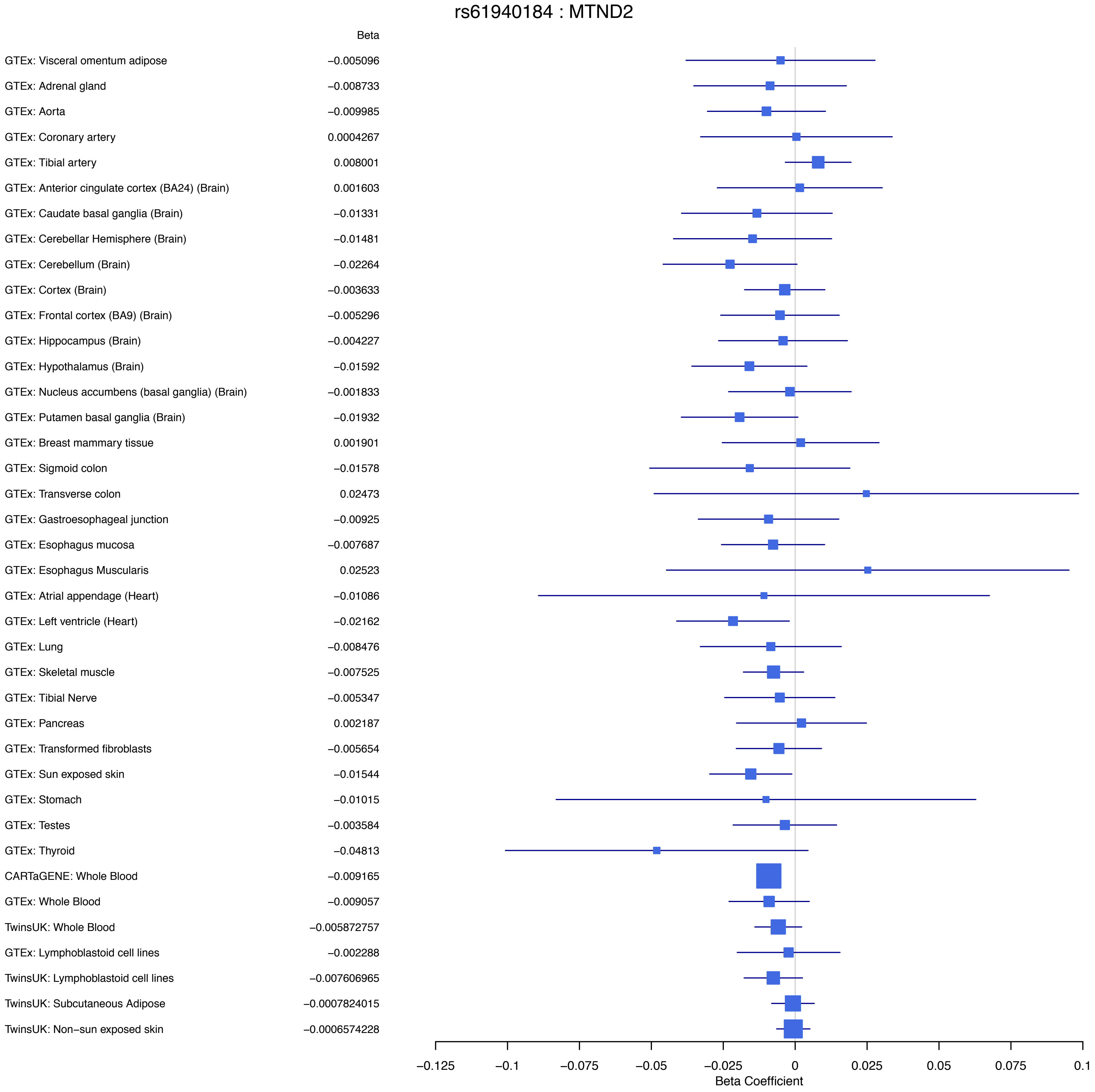
rs61940184 : MTND2
Beta
GTEx: Visceral omentum adipose −0.005096
GTEx: Adrenal gland −0.008733
GTEx: Aorta −0.009985
GTEx: Coronary artery 0.0004267
GTEx: Tibial artery 0.008001
GTEx: Anterior cingulate cortex (BA24) (Brain) 0.001603
GTEx: Caudate basal ganglia (Brain) −0.01331
GTEx: Cerebellar Hemisphere (Brain) −0.01481
GTEx: Cerebellum (Brain) −0.02264
GTEx: Cortex (Brain) −0.003633
GTEx: Frontal cortex (BA9) (Brain) −0.005296
GTEx: Hippocampus (Brain) −0.004227
GTEx: Hypothalamus (Brain) −0.01592
GTEx: Nucleus accumbens (basal ganglia) (Brain) −0.001833
GTEx: Putamen basal ganglia (Brain) −0.01932
GTEx: Breast mammary tissue 0.001901
GTEx: Sigmoid colon −0.01578
GTEx: Transverse colon 0.02473
GTEx: Gastroesophageal junction −0.00925
GTEx: Esophagus mucosa −0.007687
GTEx: Esophagus Muscularis 0.02523
GTEx: Atrial appendage (Heart) −0.01086
GTEx: Left ventricle (Heart) −0.02162
GTEx: Lung −0.008476
GTEx: Skeletal muscle −0.007525
GTEx: Tibial Nerve −0.005347
GTEx: Pancreas 0.002187
GTEx: Transformed fibroblasts −0.005654
GTEx: Sun exposed skin −0.01544
GTEx: Stomach −0.01015
GTEx: Testes −0.003584
GTEx: Thyroid −0.04813
CARTaGENE: Whole Blood −0.009165
GTEx: Whole Blood −0.009057
TwinsUK: Whole Blood −0.005872757
GTEx: Lymphoblastoid cell lines −0.002288
TwinsUK: Lymphoblastoid cell lines −0.007606965
TwinsUK: Subcutaneous Adipose −0.0007824015
TwinsUK: Non−sun exposed skin −0.0006574228
−0.125
−0.1
−0.075
−0.05
−0.025
0
0.025
0.05
0.075
0.1
Beta Coefficient

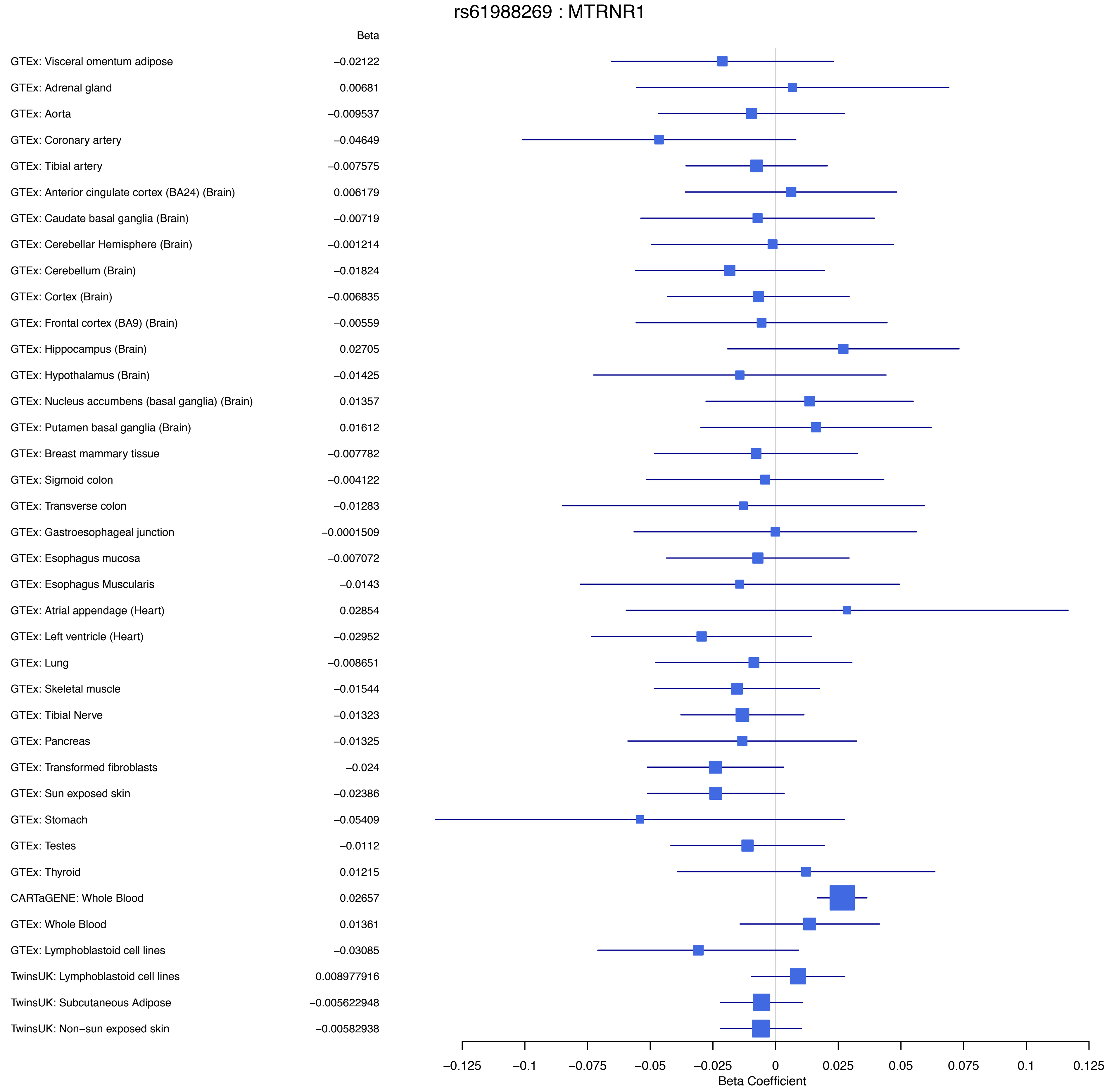
rs61988269 : MTRNR1
Beta
GTEx: Visceral omentum adipose -0.02122
GTEx: Adrenal gland 0.00681
GTEx: Aorta -0.009537
GTEx: Coronary artery -0.04649
GTEx: Tibial artery -0.007575
GTEx: Anterior cingulate cortex (BA24) (Brain) 0.006179
GTEx: Caudate basal ganglia (Brain) -0.00719
GTEx: Cerebellar Hemisphere (Brain) -0.001214
GTEx: Cerebellum (Brain) -0.01824
GTEx: Cortex (Brain) -0.006835
GTEx: Frontal cortex (BA9) (Brain) -0.00559
GTEx: Hippocampus (Brain) 0.02705
GTEx: Hypothalamus (Brain) -0.01425
GTEx: Nucleus accumbens (basal ganglia) (Brain) 0.01357
GTEx: Putamen basal ganglia (Brain) 0.01612
GTEx: Breast mammary tissue -0.007782
GTEx: Sigmoid colon -0.004122
GTEx: Transverse colon -0.01283
GTEx: Gastroesophageal junction -0.0001509
GTEx: Esophagus mucosa -0.007072
GTEx: Esophagus Muscularis -0.0143
GTEx: Atrial appendage (Heart) 0.02854
GTEx: Left ventricle (Heart) -0.02952
GTEx: Lung -0.008651
GTEx: Skeletal muscle -0.01544
GTEx: Tibial Nerve -0.01323
GTEx: Pancreas -0.01325
GTEx: Transformed fibroblasts -0.024
GTEx: Sun exposed skin -0.02386
GTEx: Stomach -0.05409
GTEx: Testes -0.0112
GTEx: Thyroid 0.01215
CARTaGENE: Whole Blood 0.02657
GTEx: Whole Blood 0.01361
GTEx: Lymphoblastoid cell lines -0.03085
TwinsUK: Lymphoblastoid cell lines 0.008977916
TwinsUK: Subcutaneous Adipose -0.005622948
TwinsUK: Non-sun exposed skin -0.00582938
-0.125 -0.1 -0.075 -0.05 -0.025 0 0.025 0.05 0.075 0.1 0.125
Beta Coefficient

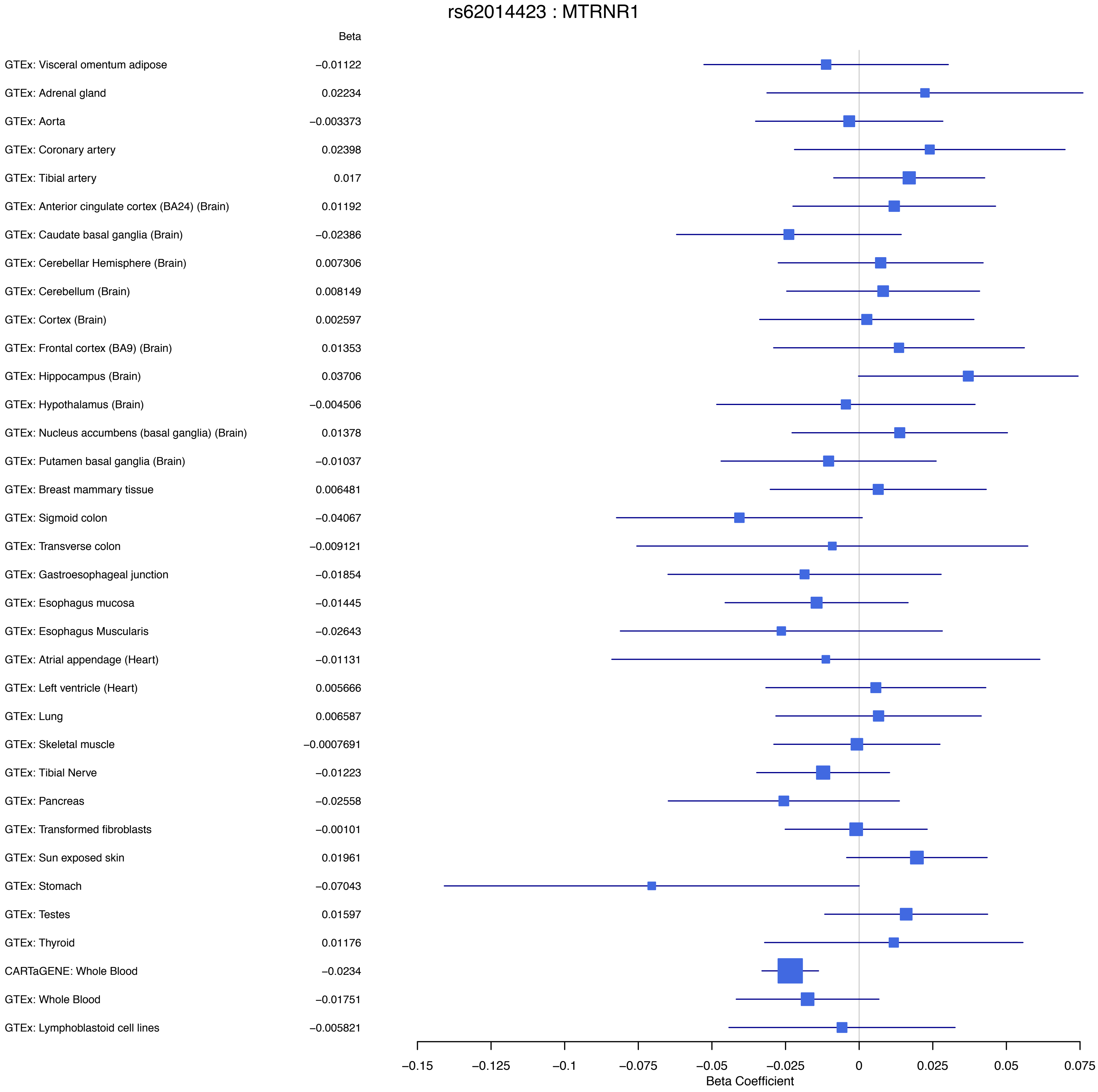
rs62014423 : MTRNR1
Beta
GTEx: Visceral omentum adipose −0.01122
GTEx: Adrenal gland 0.02234
GTEx: Aorta −0.003373
GTEx: Coronary artery 0.02398
GTEx: Tibial artery 0.017
GTEx: Anterior cingulate cortex (BA24) (Brain) 0.01192
GTEx: Caudate basal ganglia (Brain) −0.02386
GTEx: Cerebellar Hemisphere (Brain) 0.007306
GTEx: Cerebellum (Brain) 0.008149
GTEx: Cortex (Brain) 0.002597
GTEx: Frontal cortex (BA9) (Brain) 0.01353
GTEx: Hippocampus (Brain) 0.03706
GTEx: Hypothalamus (Brain) −0.004506
GTEx: Nucleus accumbens (basal ganglia) (Brain) 0.01378
GTEx: Putamen basal ganglia (Brain) −0.01037
GTEx: Breast mammary tissue 0.006481
GTEx: Sigmoid colon −0.04067
GTEx: Transverse colon −0.009121
GTEx: Gastroesophageal junction −0.01854
GTEx: Esophagus mucosa −0.01445
GTEx: Esophagus Muscularis −0.02643
GTEx: Atrial appendage (Heart) −0.01131
GTEx: Left ventricle (Heart) 0.005666
GTEx: Lung 0.006587
GTEx: Skeletal muscle −0.0007691
GTEx: Tibial Nerve −0.01223
GTEx: Pancreas −0.02558
GTEx: Transformed fibroblasts −0.00101
GTEx: Sun exposed skin 0.01961
GTEx: Stomach −0.07043
GTEx: Testes 0.01597
GTEx: Thyroid 0.01176
CARTaGENE: Whole Blood −0.0234
GTEx: Whole Blood −0.01751
GTEx: Lymphoblastoid cell lines −0.005821
−0.15 −0.125 −0.1 −0.075 −0.05 −0.025 0 0.025 0.05 0.075
Beta Coefficient

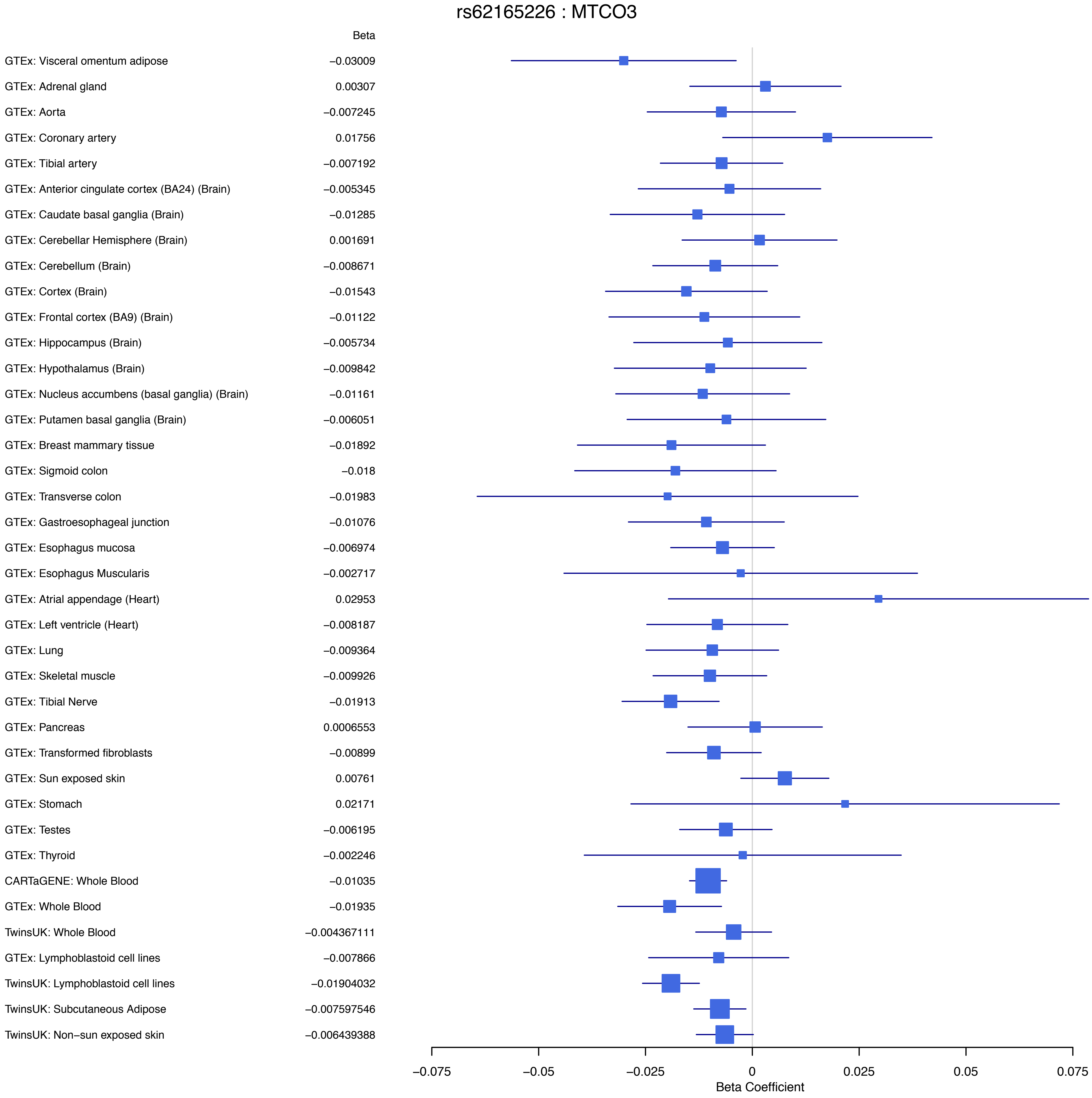
rs62165226 : MTCO3
Beta
GTEx: Visceral omentum adipose −0.03009
GTEx: Adrenal gland 0.00307
GTEx: Aorta −0.007245
GTEx: Coronary artery 0.01756
GTEx: Tibial artery −0.007192
GTEx: Anterior cingulate cortex (BA24) (Brain) −0.005345
GTEx: Caudate basal ganglia (Brain) −0.01285
GTEx: Cerebellar Hemisphere (Brain) 0.001691
GTEx: Cerebellum (Brain) −0.008671
GTEx: Cortex (Brain) −0.01543
GTEx: Frontal cortex (BA9) (Brain) −0.01122
GTEx: Hippocampus (Brain) −0.005734
GTEx: Hypothalamus (Brain) −0.009842
GTEx: Nucleus accumbens (basal ganglia) (Brain) −0.01161
GTEx: Putamen basal ganglia (Brain) −0.006051
GTEx: Breast mammary tissue −0.01892
GTEx: Sigmoid colon −0.018
GTEx: Transverse colon −0.01983
GTEx: Gastroesophageal junction −0.01076
GTEx: Esophagus mucosa −0.006974
GTEx: Esophagus Muscularis −0.002717
GTEx: Atrial appendage (Heart) 0.02953
GTEx: Left ventricle (Heart) −0.008187
GTEx: Lung −0.009364
GTEx: Skeletal muscle −0.009926
GTEx: Tibial Nerve −0.01913
GTEx: Pancreas 0.0006553
GTEx: Transformed fibroblasts −0.00899
GTEx: Sun exposed skin 0.00761
GTEx: Stomach 0.02171
GTEx: Testes −0.006195
GTEx: Thyroid −0.002246
CARTaGENE: Whole Blood −0.01035
GTEx: Whole Blood −0.01935
TwinsUK: Whole Blood −0.004367111
GTEx: Lymphoblastoid cell lines −0.007866
TwinsUK: Lymphoblastoid cell lines −0.01904032
TwinsUK: Subcutaneous Adipose −0.007597546
TwinsUK: Non−sun exposed skin −0.006439388
−0.075
−0.05
−0.025
0
0.025
0.05
0.075
Beta Coefficient

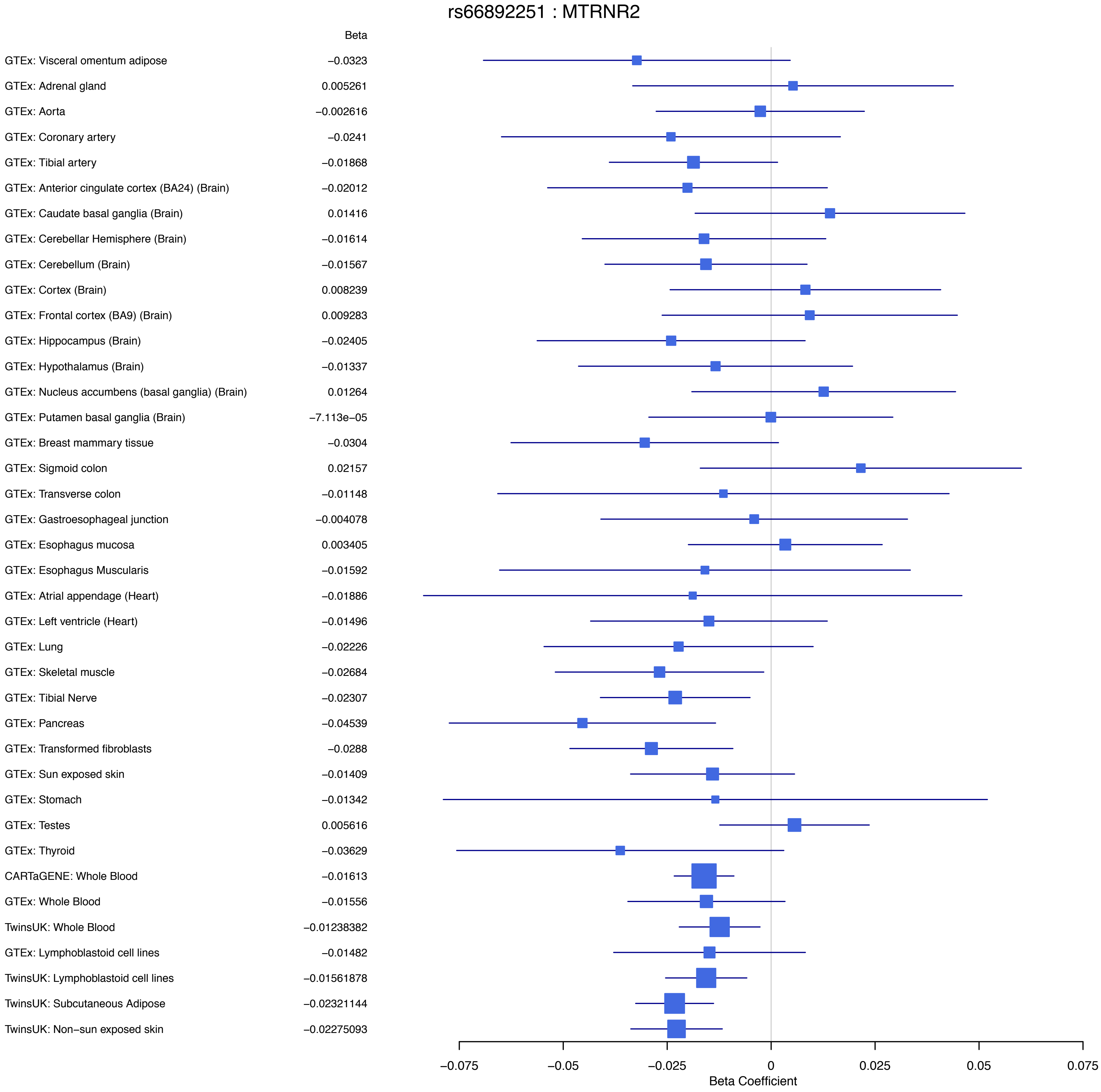
rs66892251 : MTRNR2
Beta
GTEx: Visceral omentum adipose −0.0323
GTEx: Adrenal gland 0.005261
GTEx: Aorta −0.002616
GTEx: Coronary artery −0.0241
GTEx: Tibial artery −0.01868
GTEx: Anterior cingulate cortex (BA24) (Brain) −0.02012
GTEx: Caudate basal ganglia (Brain) 0.01416
GTEx: Cerebellar Hemisphere (Brain) −0.01614
GTEx: Cerebellum (Brain) −0.01567
GTEx: Cortex (Brain) 0.008239
GTEx: Frontal cortex (BA9) (Brain) 0.009283
GTEx: Hippocampus (Brain) −0.02405
GTEx: Hypothalamus (Brain) −0.01337
GTEx: Nucleus accumbens (basal ganglia) (Brain) 0.01264
GTEx: Putamen basal ganglia (Brain) −7.113e−05
GTEx: Breast mammary tissue −0.0304
GTEx: Sigmoid colon 0.02157
GTEx: Transverse colon −0.01148
GTEx: Gastroesophageal junction −0.004078
GTEx: Esophagus mucosa 0.003405
GTEx: Esophagus Muscularis −0.01592
GTEx: Atrial appendage (Heart) −0.01886
GTEx: Left ventricle (Heart) −0.01496
GTEx: Lung −0.02226
GTEx: Skeletal muscle −0.02684
GTEx: Tibial Nerve −0.02307
GTEx: Pancreas −0.04539
GTEx: Transformed fibroblasts −0.0288
GTEx: Sun exposed skin −0.01409
GTEx: Stomach −0.01342
GTEx: Testes 0.005616
GTEx: Thyroid −0.03629
CARTaGENE: Whole Blood −0.01613
GTEx: Whole Blood −0.01556
TwinsUK: Whole Blood −0.01238382
GTEx: Lymphoblastoid cell lines −0.01482
TwinsUK: Lymphoblastoid cell lines −0.01561878
TwinsUK: Subcutaneous Adipose −0.02321144
TwinsUK: Non−sun exposed skin −0.02275093
−0.075
−0.05
−0.025
0
0.025
0.05
0.075
Beta Coefficient

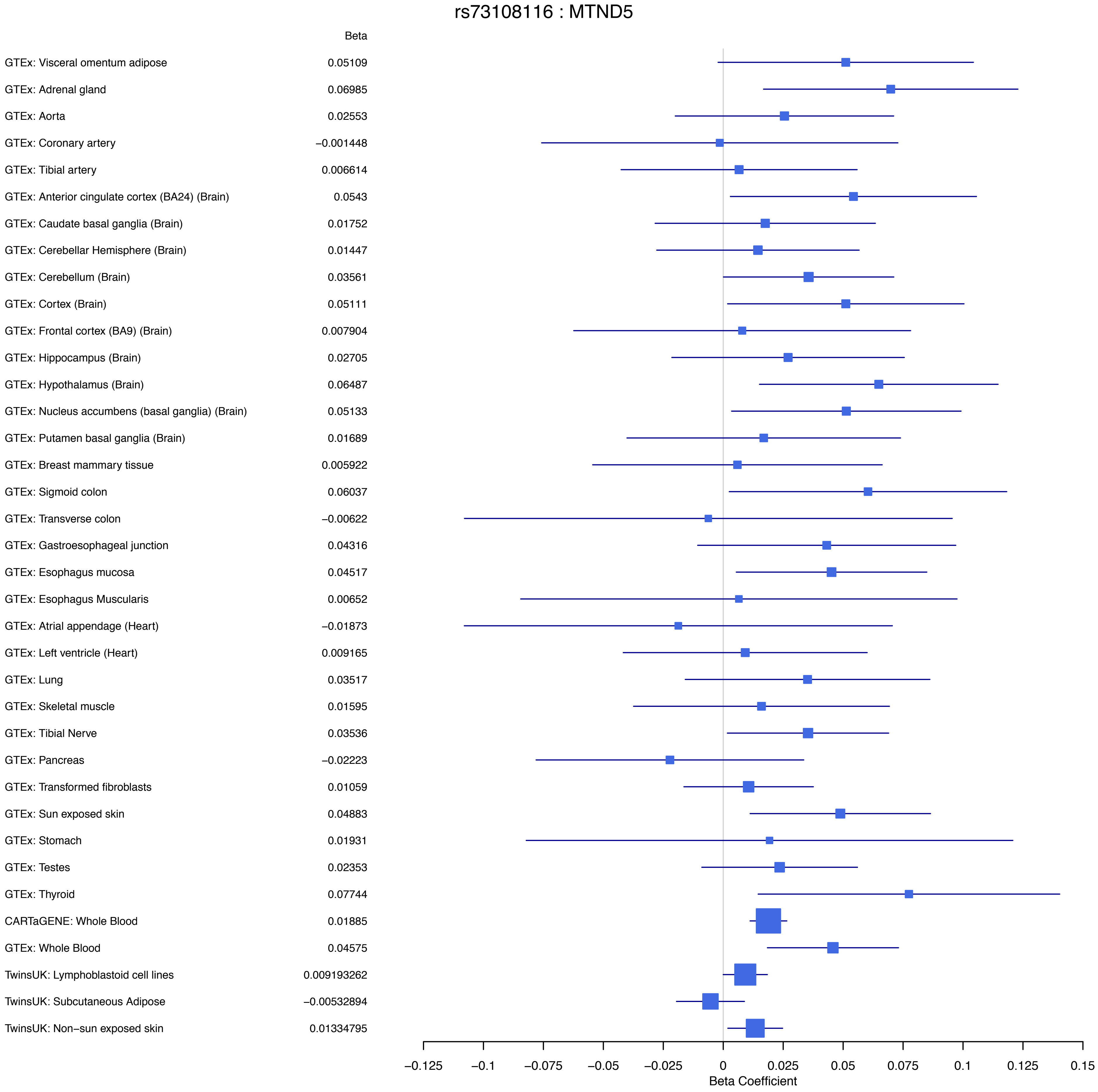

rs73108116 : MTND5
Beta
GTEx: Visceral omentum adipose 0.05109
GTEx: Adrenal gland 0.06985
GTEx: Aorta 0.02553
GTEx: Coronary artery −0.001448
GTEx: Tibial artery 0.006614
GTEx: Anterior cingulate cortex (BA24) (Brain) 0.0543
GTEx: Caudate basal ganglia (Brain) 0.01752
GTEx: Cerebellar Hemisphere (Brain) 0.01447
GTEx: Cerebellum (Brain) 0.03561
GTEx: Cortex (Brain) 0.05111
GTEx: Frontal cortex (BA9) (Brain) 0.007904
GTEx: Hippocampus (Brain) 0.02705
GTEx: Hypothalamus (Brain) 0.06487
GTEx: Nucleus accumbens (basal ganglia) (Brain) 0.05133
GTEx: Putamen basal ganglia (Brain) 0.01689
GTEx: Breast mammary tissue 0.005922
GTEx: Sigmoid colon 0.06037
GTEx: Transverse colon −0.00622
GTEx: Gastroesophageal junction 0.04316
GTEx: Esophagus mucosa 0.04517
GTEx: Esophagus Muscularis 0.00652
GTEx: Atrial appendage (Heart) −0.01873
GTEx: Left ventricle (Heart) 0.009165
GTEx: Lung 0.03517
GTEx: Skeletal muscle 0.01595
GTEx: Tibial Nerve 0.03536
GTEx: Pancreas −0.02223
GTEx: Transformed fibroblasts 0.01059
GTEx: Sun exposed skin 0.04883
GTEx: Stomach 0.01931
GTEx: Testes 0.02353
GTEx: Thyroid 0.07744
CARTaGENE: Whole Blood 0.01885
GTEx: Whole Blood 0.04575
TwinsUK: Lymphoblastoid cell lines 0.009193262
TwinsUK: Subcutaneous Adipose −0.00532894
TwinsUK: Non−sun exposed skin 0.01334795
−0.125 −0.1 −0.075 −0.05 −0.025 0 0.025 0.05 0.075 0.1 0.125 0.15
Beta Coefficient

rs74025341 : MTCYB

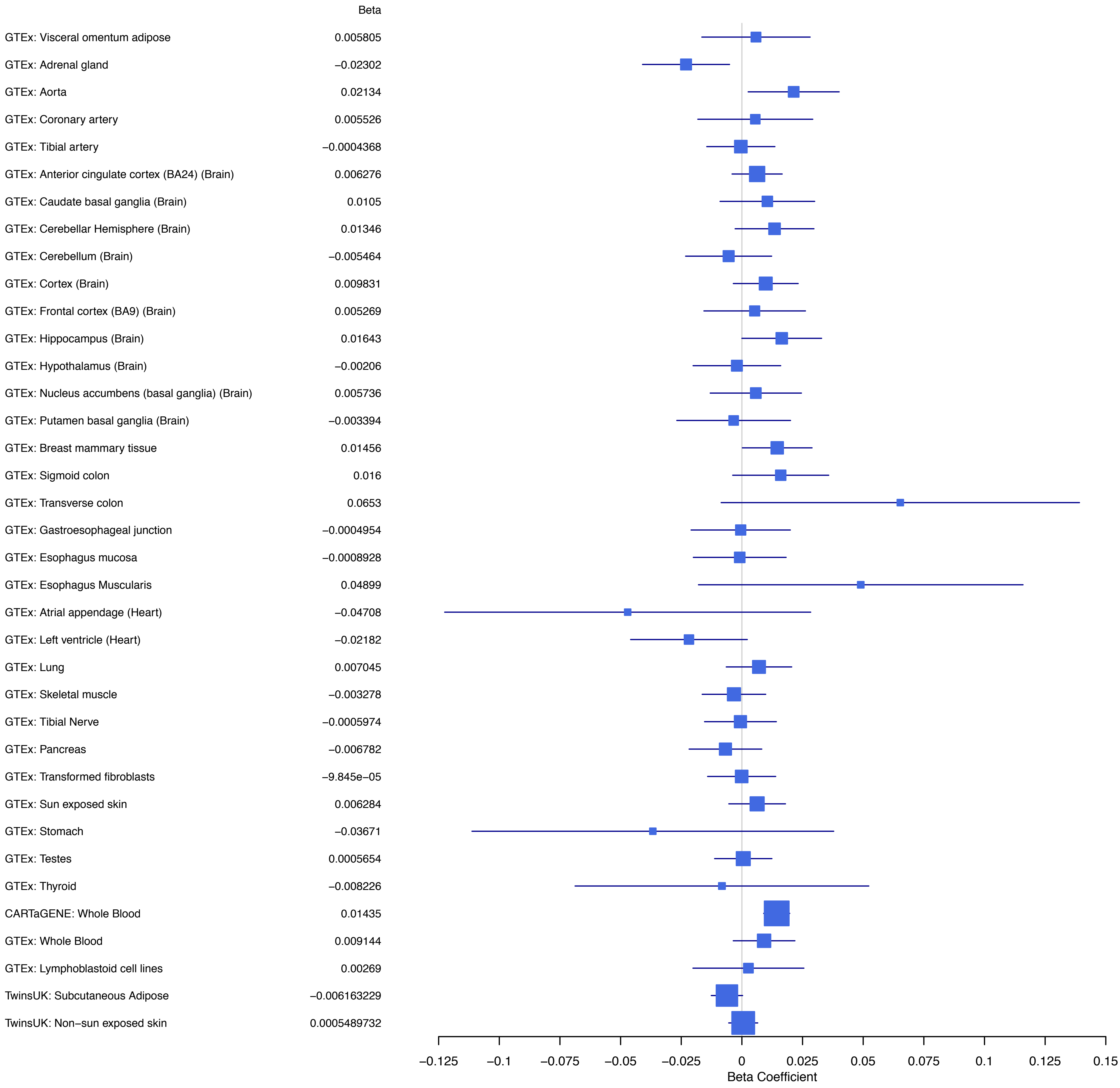

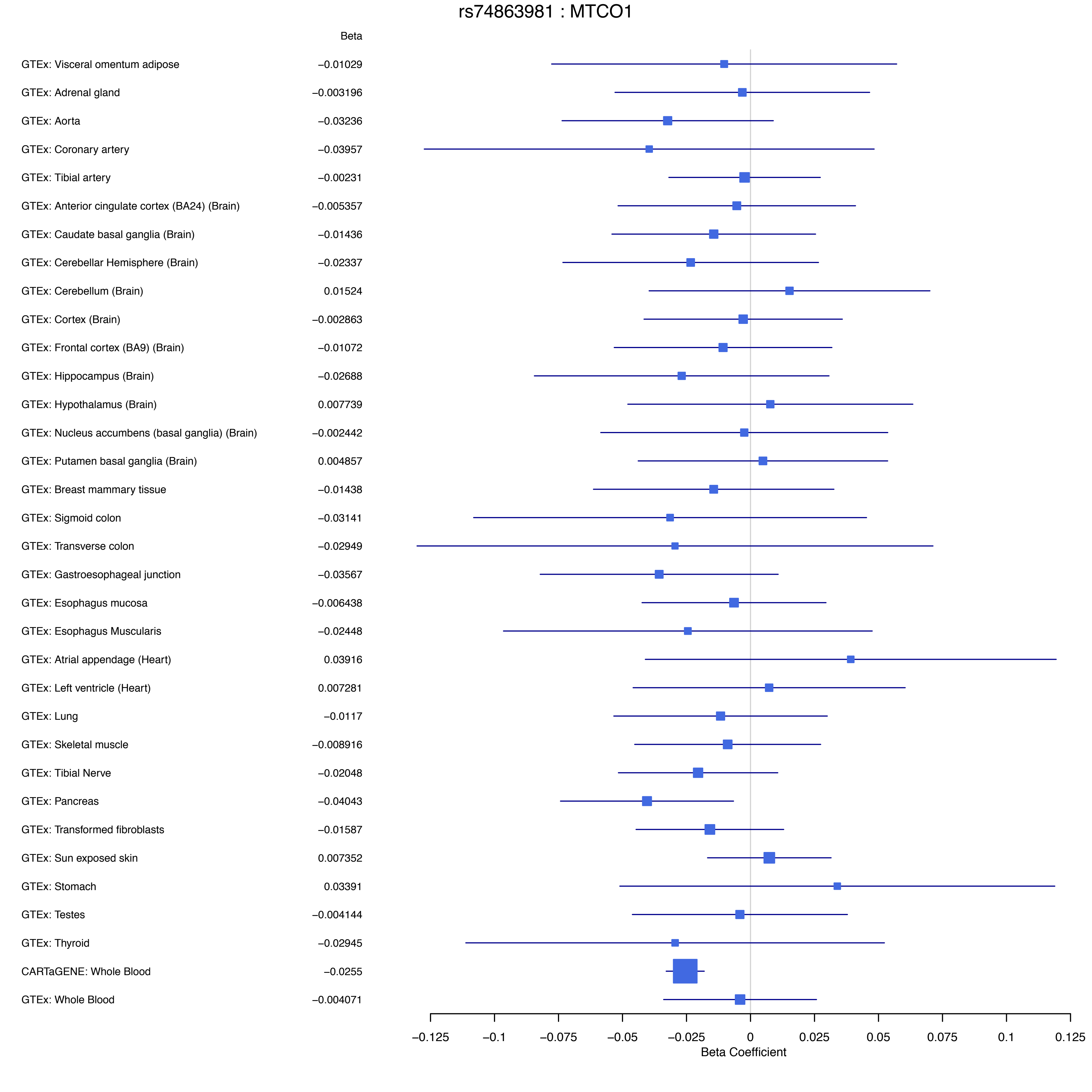
rs74863981 : MTCO1
Beta
GTEx: Visceral omentum adipose −0.01029
GTEx: Adrenal gland −0.003196
GTEx: Aorta −0.03236
GTEx: Coronary artery −0.03957
GTEx: Tibial artery −0.00231
GTEx: Anterior cingulate cortex (BA24) (Brain) −0.005357
GTEx: Caudate basal ganglia (Brain) −0.01436
GTEx: Cerebellar Hemisphere (Brain) −0.02337
GTEx: Cerebellum (Brain) 0.01524
GTEx: Cortex (Brain) −0.002863
GTEx: Frontal cortex (BA9) (Brain) −0.01072
GTEx: Hippocampus (Brain) −0.02688
GTEx: Hypothalamus (Brain) 0.007739
GTEx: Nucleus accumbens (basal ganglia) (Brain) −0.002442
GTEx: Putamen basal ganglia (Brain) 0.004857
GTEx: Breast mammary tissue −0.01438
GTEx: Sigmoid colon −0.03141
GTEx: Transverse colon −0.02949
GTEx: Gastroesophageal junction −0.03567
GTEx: Esophagus mucosa −0.006438
GTEx: Esophagus Muscularis −0.02448
GTEx: Atrial appendage (Heart) 0.03916
GTEx: Left ventricle (Heart) 0.007281
GTEx: Lung −0.0117
GTEx: Skeletal muscle −0.008916
GTEx: Tibial Nerve −0.02048
GTEx: Pancreas −0.04043
GTEx: Transformed fibroblasts −0.01587
GTEx: Sun exposed skin 0.007352
GTEx: Stomach 0.03391
GTEx: Testes −0.004144
GTEx: Thyroid −0.02945
CARTaGENE: Whole Blood −0.0255
GTEx: Whole Blood −0.004071
−0.125
−0.1
−0.075
−0.05
−0.025
0
0.025
0.05
0.075
0.1
0.125
Beta Coefficient

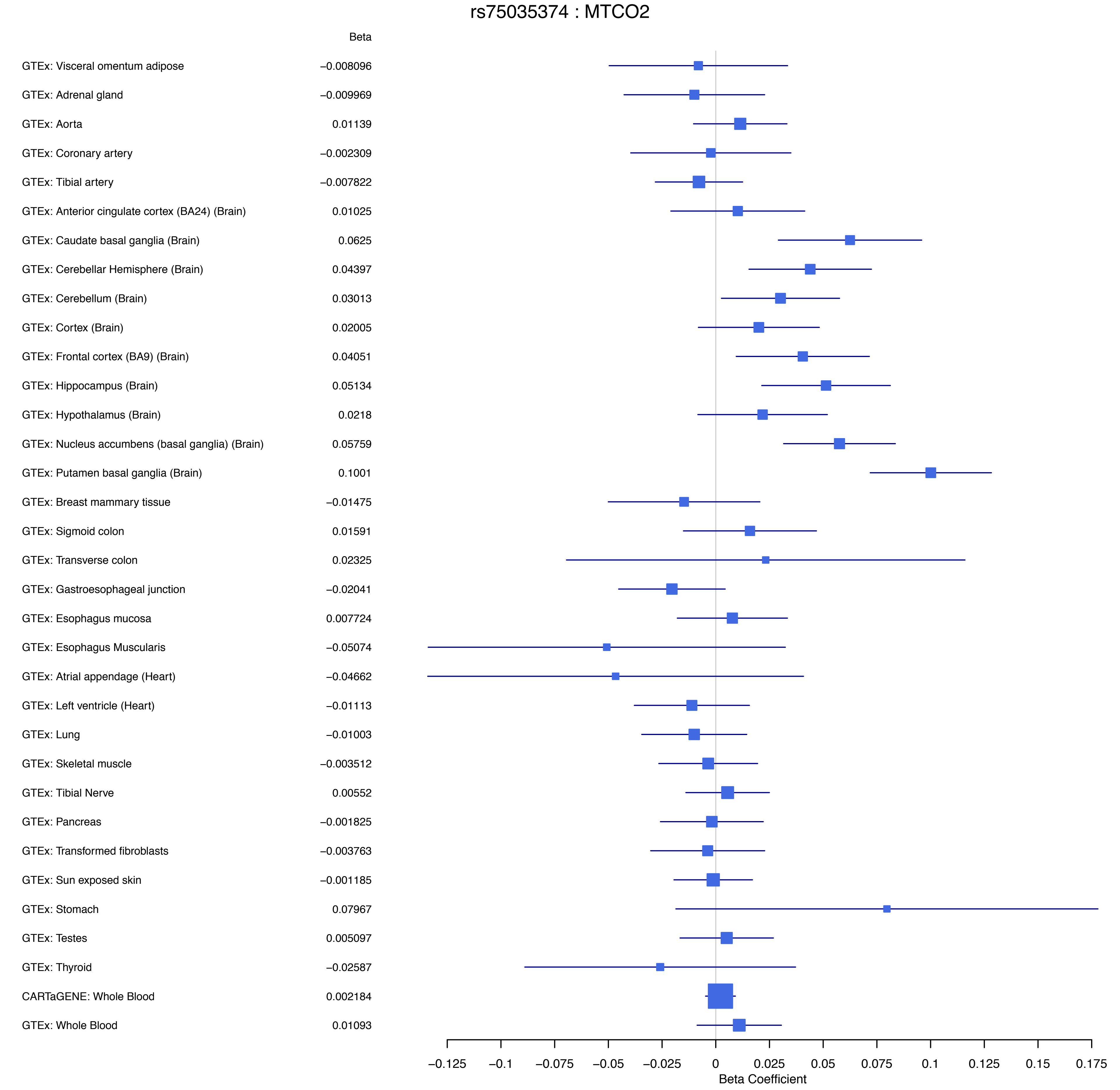
rs75035374 : MTCO2
Beta
GTEx: Visceral omentum adipose
−0.008096
GTEx: Adrenal gland
−0.009969
GTEx: Aorta
0.01139
GTEx: Coronary artery
−0.002309
GTEx: Tibial artery
−0.007822
GTEx: Anterior cingulate cortex (BA24) (Brain)
0.01025
GTEx: Caudate basal ganglia (Brain)
0.0625
GTEx: Cerebellar Hemisphere (Brain)
0.04397
GTEx: Cerebellum (Brain)
0.03013
GTEx: Cortex (Brain)
0.02005
GTEx: Frontal cortex (BA9) (Brain)
0.04051
GTEx: Hippocampus (Brain)
0.05134
GTEx: Hypothalamus (Brain)
0.0218
GTEx: Nucleus accumbens (basal ganglia) (Brain)
0.05759
GTEx: Putamen basal ganglia (Brain)
0.1001
GTEx: Breast mammary tissue
−0.01475
GTEx: Sigmoid colon
0.01591
GTEx: Transverse colon
0.02325
GTEx: Gastroesophageal junction
−0.02041
GTEx: Esophagus mucosa
0.007724
GTEx: Esophagus Muscularis
−0.05074
GTEx: Atrial appendage (Heart)
−0.04662
GTEx: Left ventricle (Heart)
−0.01113
GTEx: Lung
−0.01003
GTEx: Skeletal muscle
−0.003512
GTEx: Tibial Nerve
0.00552
GTEx: Pancreas
−0.001825
GTEx: Transformed fibroblasts
−0.003763
GTEx: Sun exposed skin
−0.001185
GTEx: Stomach
0.07967
GTEx: Testes
0.005097
GTEx: Thyroid
−0.02587
CARTaGENE: Whole Blood
0.002184
GTEx: Whole Blood
0.01093
−0.125
−0.1
−0.075
−0.05
−0.025
0
0.025
0.05
0.075
0.1
0.125
0.15
0.175
Beta Coefficient

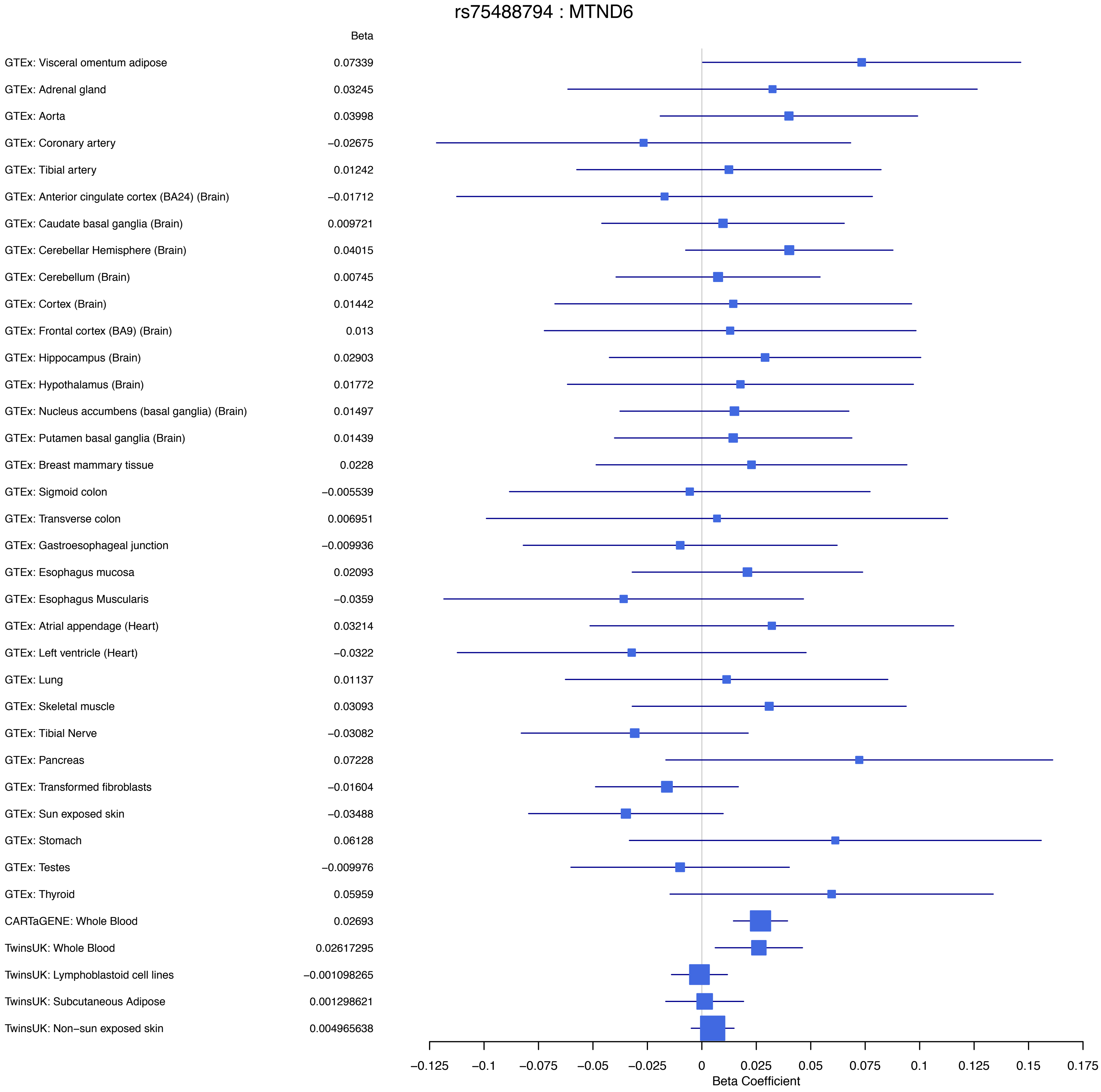
rs75488794 : MTND6
Beta
GTEx: Visceral omentum adipose 0.07339
GTEx: Adrenal gland 0.03245
GTEx: Aorta 0.03998
GTEx: Coronary artery −0.02675
GTEx: Tibial artery 0.01242
GTEx: Anterior cingulate cortex (BA24) (Brain) −0.01712
GTEx: Caudate basal ganglia (Brain) 0.009721
GTEx: Cerebellar Hemisphere (Brain) 0.04015
GTEx: Cerebellum (Brain) 0.00745
GTEx: Cortex (Brain) 0.01442
GTEx: Frontal cortex (BA9) (Brain) 0.013
GTEx: Hippocampus (Brain) 0.02903
GTEx: Hypothalamus (Brain) 0.01772
GTEx: Nucleus accumbens (basal ganglia) (Brain) 0.01497
GTEx: Putamen basal ganglia (Brain) 0.01439
GTEx: Breast mammary tissue 0.0228
GTEx: Sigmoid colon −0.005539
GTEx: Transverse colon 0.006951
GTEx: Gastroesophageal junction −0.009936
GTEx: Esophagus mucosa 0.02093
GTEx: Esophagus Muscularis −0.0359
GTEx: Atrial appendage (Heart) 0.03214
GTEx: Left ventricle (Heart) −0.0322
GTEx: Lung 0.01137
GTEx: Skeletal muscle 0.03093
GTEx: Tibial Nerve −0.03082
GTEx: Pancreas 0.07228
GTEx: Transformed fibroblasts −0.01604
GTEx: Sun exposed skin −0.03488
GTEx: Stomach 0.06128
GTEx: Testes −0.009976
GTEx: Thyroid 0.05959
CARTaGENE: Whole Blood 0.02693
TwinsUK: Whole Blood 0.02617295
TwinsUK: Lymphoblastoid cell lines −0.001098265
TwinsUK: Subcutaneous Adipose 0.001298621
TwinsUK: Non−sun exposed skin 0.004965638
−0.125 −0.1 −0.075 −0.05 −0.025 0 0.025 0.05 0.075 0.1 0.125 0.15 0.175
Beta Coefficient

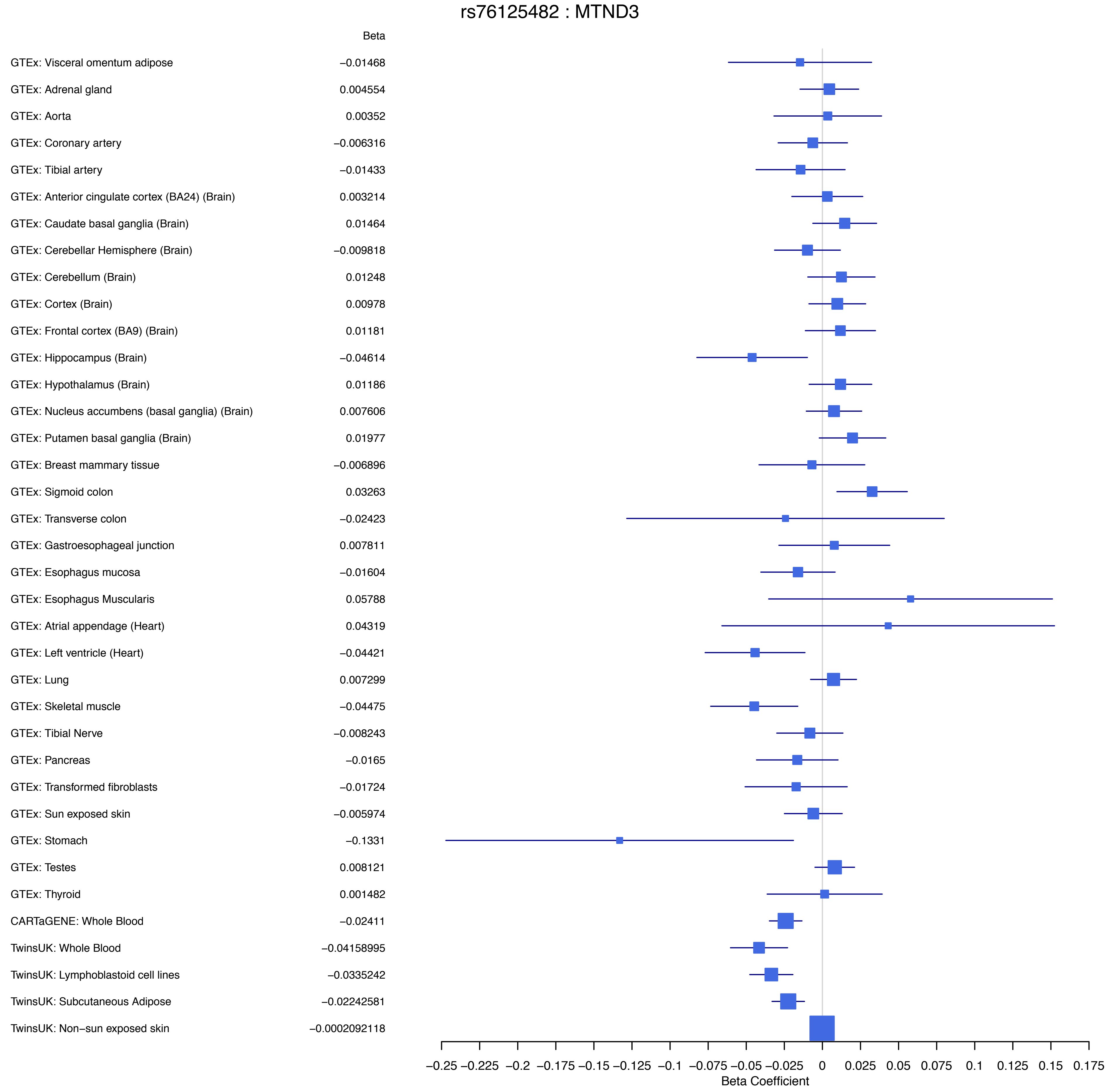
rs76125482 : MTND3
Beta
GTEx: Visceral omentum adipose −0.01468
GTEx: Adrenal gland 0.004554
GTEx: Aorta 0.00352
GTEx: Coronary artery −0.006316
GTEx: Tibial artery −0.01433
GTEx: Anterior cingulate cortex (BA24) (Brain) 0.003214
GTEx: Caudate basal ganglia (Brain) 0.01464
GTEx: Cerebellar Hemisphere (Brain) −0.009818
GTEx: Cerebellum (Brain) 0.01248
GTEx: Cortex (Brain) 0.00978
GTEx: Frontal cortex (BA9) (Brain) 0.01181
GTEx: Hippocampus (Brain) −0.04614
GTEx: Hypothalamus (Brain) 0.01186
GTEx: Nucleus accumbens (basal ganglia) (Brain) 0.007606
GTEx: Putamen basal ganglia (Brain) 0.01977
GTEx: Breast mammary tissue −0.006896
GTEx: Sigmoid colon 0.03263
GTEx: Transverse colon −0.02423
GTEx: Gastroesophageal junction 0.007811
GTEx: Esophagus mucosa −0.01604
GTEx: Esophagus Muscularis 0.05788
GTEx: Atrial appendage (Heart) 0.04319
GTEx: Left ventricle (Heart) −0.04421
GTEx: Lung 0.007299
GTEx: Skeletal muscle −0.04475
GTEx: Tibial Nerve −0.008243
GTEx: Pancreas −0.0165
GTEx: Transformed fibroblasts −0.01724
GTEx: Sun exposed skin −0.005974
GTEx: Stomach −0.1331
GTEx: Testes 0.008121
GTEx: Thyroid 0.001482
CARTaGENE: Whole Blood −0.02411
TwinsUK: Whole Blood −0.04158995
TwinsUK: Lymphoblastoid cell lines −0.0335242
TwinsUK: Subcutaneous Adipose −0.02242581
TwinsUK: Non−sun exposed skin −0.0002092118
−0.25 −0.225 −0.2 −0.175 −0.15 −0.125 −0.1 −0.075 −0.05 −0.025 0 0.025 0.05 0.075 0.1 0.125 0.15 0.175
Beta Coefficient

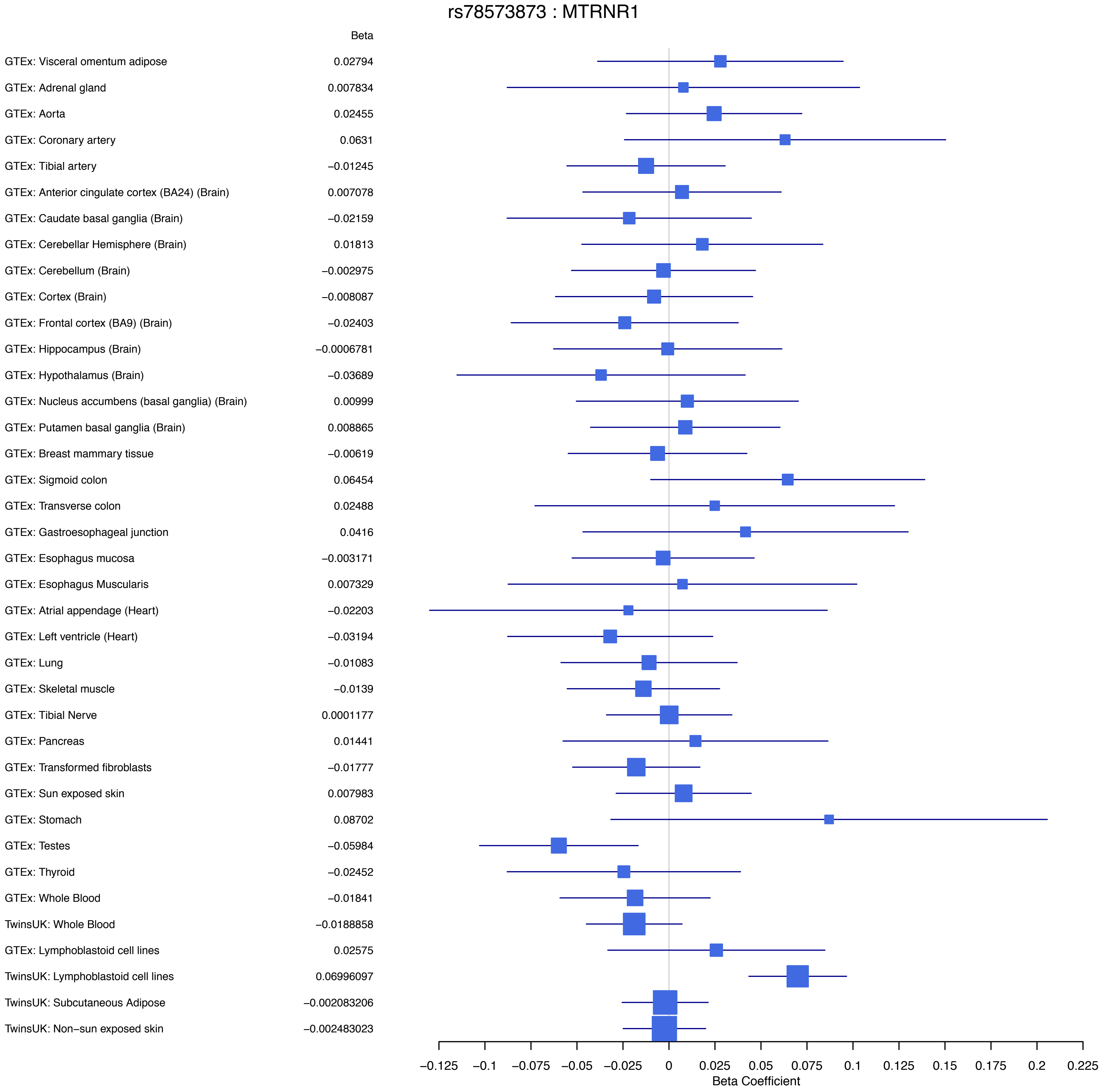
rs78573873 : MTRNR1
Beta
GTEx: Visceral omentum adipose 0.02794
GTEx: Adrenal gland 0.007834
GTEx: Aorta 0.02455
GTEx: Coronary artery 0.0631
GTEx: Tibial artery −0.01245
GTEx: Anterior cingulate cortex (BA24) (Brain) 0.007078
GTEx: Caudate basal ganglia (Brain) −0.02159
GTEx: Cerebellar Hemisphere (Brain) 0.01813
GTEx: Cerebellum (Brain) −0.002975
GTEx: Cortex (Brain) −0.008087
GTEx: Frontal cortex (BA9) (Brain) −0.02403
GTEx: Hippocampus (Brain) −0.0006781
GTEx: Hypothalamus (Brain) −0.03689
GTEx: Nucleus accumbens (basal ganglia) (Brain) 0.00999
GTEx: Putamen basal ganglia (Brain) 0.008865
GTEx: Breast mammary tissue −0.00619
GTEx: Sigmoid colon 0.06454
GTEx: Transverse colon 0.02488
GTEx: Gastroesophageal junction 0.0416
GTEx: Esophagus mucosa −0.003171
GTEx: Esophagus Muscularis 0.007329
GTEx: Atrial appendage (Heart) −0.02203
GTEx: Left ventricle (Heart) −0.03194
GTEx: Lung −0.01083
GTEx: Skeletal muscle −0.0139
GTEx: Tibial Nerve 0.0001177
GTEx: Pancreas 0.01441
GTEx: Transformed fibroblasts −0.01777
GTEx: Sun exposed skin 0.007983
GTEx: Stomach 0.08702
GTEx: Testes −0.05984
GTEx: Thyroid −0.02452
GTEx: Whole Blood −0.01841
TwinsUK: Whole Blood −0.0188858
GTEx: Lymphoblastoid cell lines 0.02575
TwinsUK: Lymphoblastoid cell lines 0.06996097
TwinsUK: Subcutaneous Adipose −0.002083206
TwinsUK: Non−sun exposed skin −0.002483023
−0.125 −0.1 −0.075 −0.05 −0.025 0 0.025 0.05 0.075 0.1 0.125 0.15 0.175 0.2 0.225
Beta Coefficient

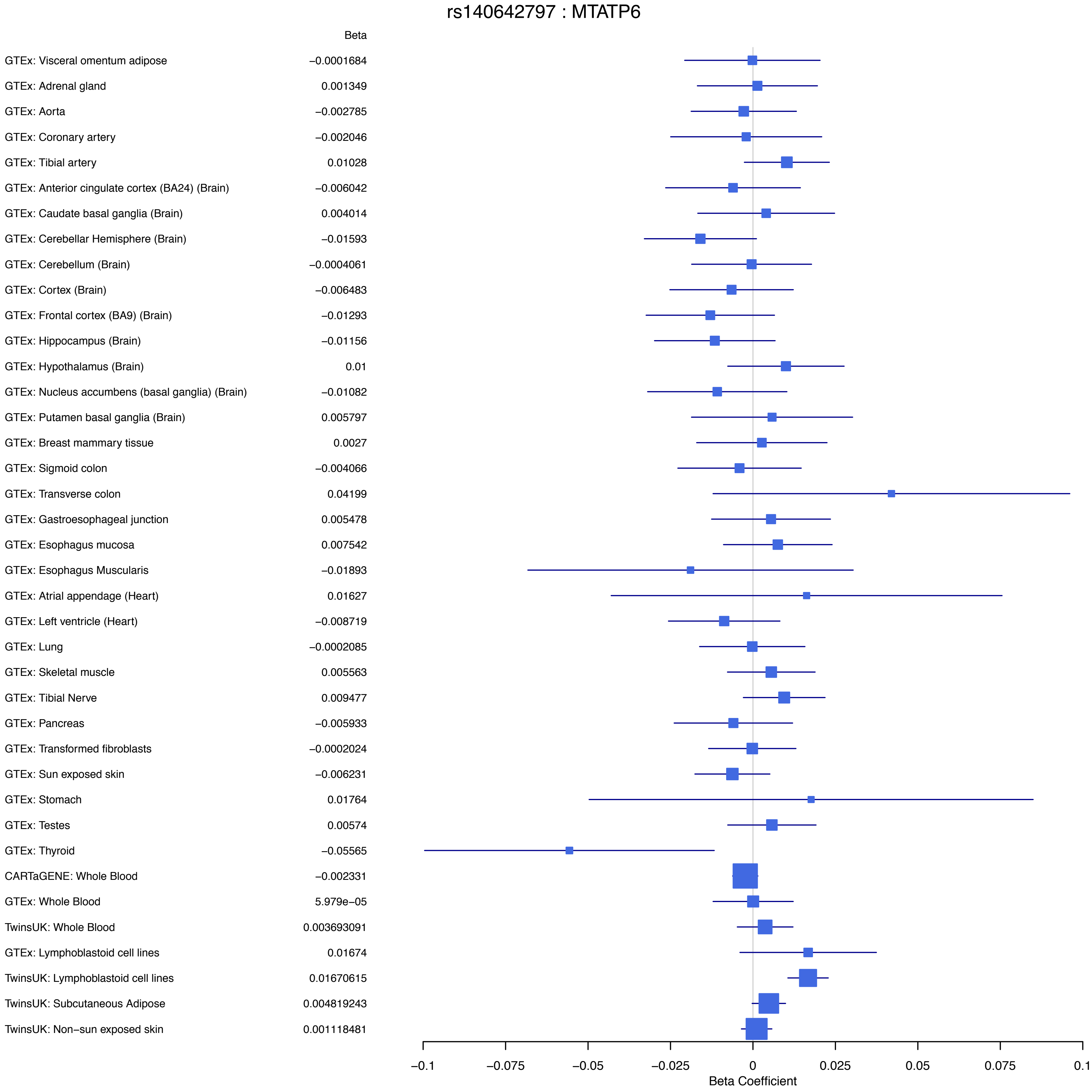
rs140642797 : MTATP6
Beta
GTEx: Visceral omentum adipose –0.0001684
GTEx: Adrenal gland 0.001349
GTEx: Aorta –0.002785
GTEx: Coronary artery –0.002046
GTEx: Tibial artery 0.01028
GTEx: Anterior cingulate cortex (BA24) (Brain) –0.006042
GTEx: Caudate basal ganglia (Brain) 0.004014
GTEx: Cerebellar Hemisphere (Brain) –0.01593
GTEx: Cerebellum (Brain) –0.0004061
GTEx: Cortex (Brain) –0.006483
GTEx: Frontal cortex (BA9) (Brain) –0.01293
GTEx: Hippocampus (Brain) –0.01156
GTEx: Hypothalamus (Brain) 0.01
GTEx: Nucleus accumbens (basal ganglia) (Brain) –0.01082
GTEx: Putamen basal ganglia (Brain) 0.005797
GTEx: Breast mammary tissue 0.0027
GTEx: Sigmoid colon –0.004066
GTEx: Transverse colon 0.04199
GTEx: Gastroesophageal junction 0.005478
GTEx: Esophagus mucosa 0.007542
GTEx: Esophagus Muscularis –0.01893
GTEx: Atrial appendage (Heart) 0.01627
GTEx: Left ventricle (Heart) –0.008719
GTEx: Lung –0.0002085
GTEx: Skeletal muscle 0.005563
GTEx: Tibial Nerve 0.009477
GTEx: Pancreas –0.005933
GTEx: Transformed fibroblasts –0.0002024
GTEx: Sun exposed skin –0.006231
GTEx: Stomach 0.01764
GTEx: Testes 0.00574
GTEx: Thyroid –0.05565
CARTaGENE: Whole Blood –0.002331
GTEx: Whole Blood 5.979e–05
TwinsUK: Whole Blood 0.003693091
GTEx: Lymphoblastoid cell lines 0.01674
TwinsUK: Lymphoblastoid cell lines 0.01670615
TwinsUK: Subcutaneous Adipose 0.004819243
TwinsUK: Non–sun exposed skin 0.001118481
–0.1
–0.075
–0.05
–0.025
0
0.025
0.05
0.075
0.1
Beta Coefficient

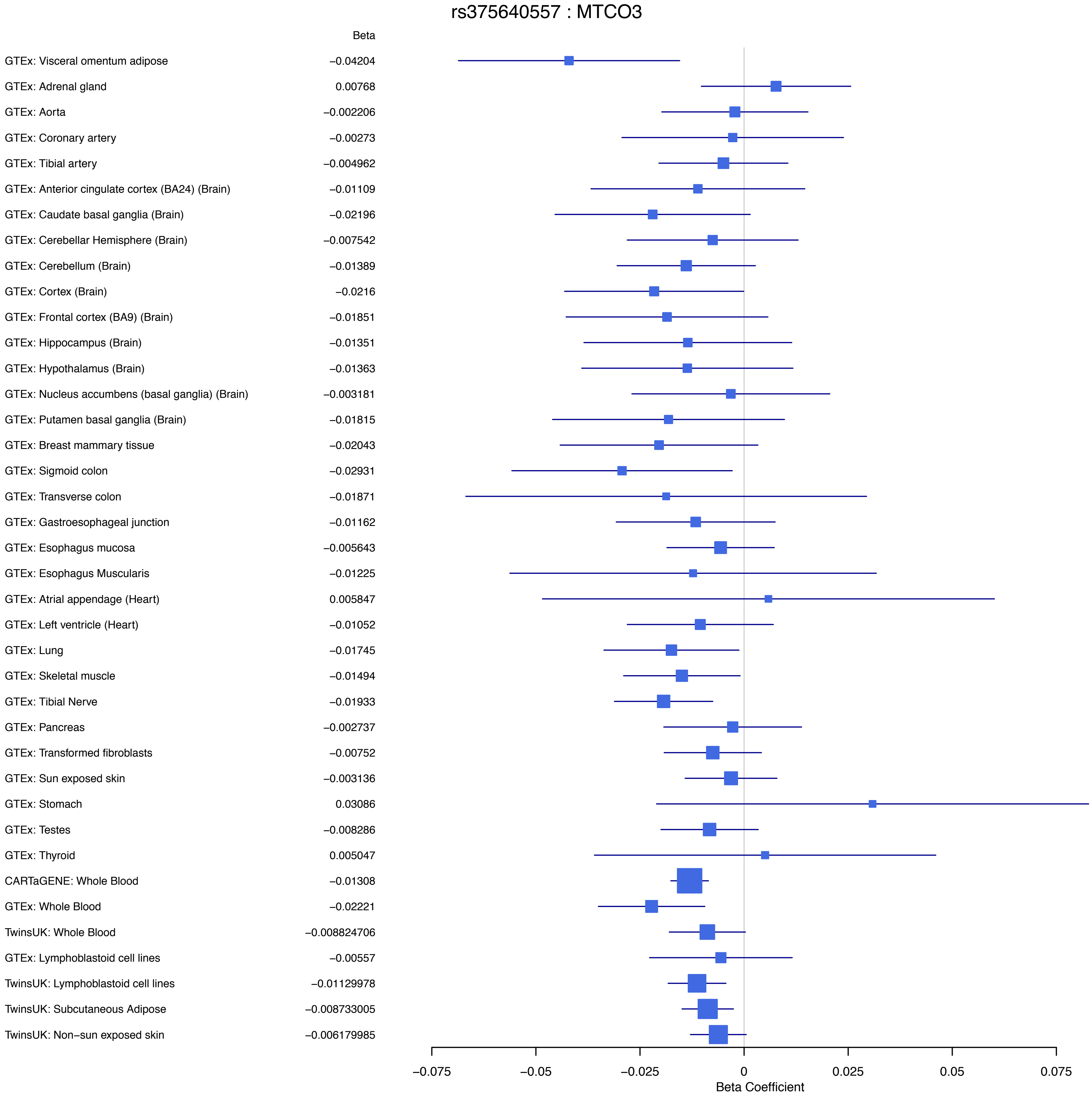
rs375640557 : MTCO3
Beta
GTEx: Visceral omentum adipose
−0.04204
GTEx: Adrenal gland
0.00768
GTEx: Aorta
−0.002206
GTEx: Coronary artery
−0.00273
GTEx: Tibial artery
−0.004962
GTEx: Anterior cingulate cortex (BA24) (Brain)
−0.01109
GTEx: Caudate basal ganglia (Brain)
−0.02196
GTEx: Cerebellar Hemisphere (Brain)
−0.007542
GTEx: Cerebellum (Brain)
−0.01389
GTEx: Cortex (Brain)
−0.0216
GTEx: Frontal cortex (BA9) (Brain)
−0.01851
GTEx: Hippocampus (Brain)
−0.01351
GTEx: Hypothalamus (Brain)
−0.01363
GTEx: Nucleus accumbens (basal ganglia) (Brain)
−0.003181
GTEx: Putamen basal ganglia (Brain)
−0.01815
GTEx: Breast mammary tissue
−0.02043
GTEx: Sigmoid colon
−0.02931
GTEx: Transverse colon
−0.01871
GTEx: Gastroesophageal junction
−0.01162
GTEx: Esophagus mucosa
−0.005643
GTEx: Esophagus Muscularis
−0.01225
GTEx: Atrial appendage (Heart)
0.005847
GTEx: Left ventricle (Heart)
−0.01052
GTEx: Lung
−0.01745
GTEx: Skeletal muscle
−0.01494
GTEx: Tibial Nerve
−0.01933
GTEx: Pancreas
−0.002737
GTEx: Transformed fibroblasts
−0.00752
GTEx: Sun exposed skin
−0.003136
GTEx: Stomach
0.03086
GTEx: Testes
−0.008286
GTEx: Thyroid
0.005047
CARTaGENE: Whole Blood
−0.01308
GTEx: Whole Blood
−0.02221
TwinsUK: Whole Blood
−0.008824706
GTEx: Lymphoblastoid cell lines
−0.00557
TwinsUK: Lymphoblastoid cell lines
−0.01129978
TwinsUK: Subcutaneous Adipose
−0.008733005
TwinsUK: Non−sun exposed skin
−0.006179985
−0.075
−0.05
−0.025
0
0.025
0.05
0.075
Beta Coefficient

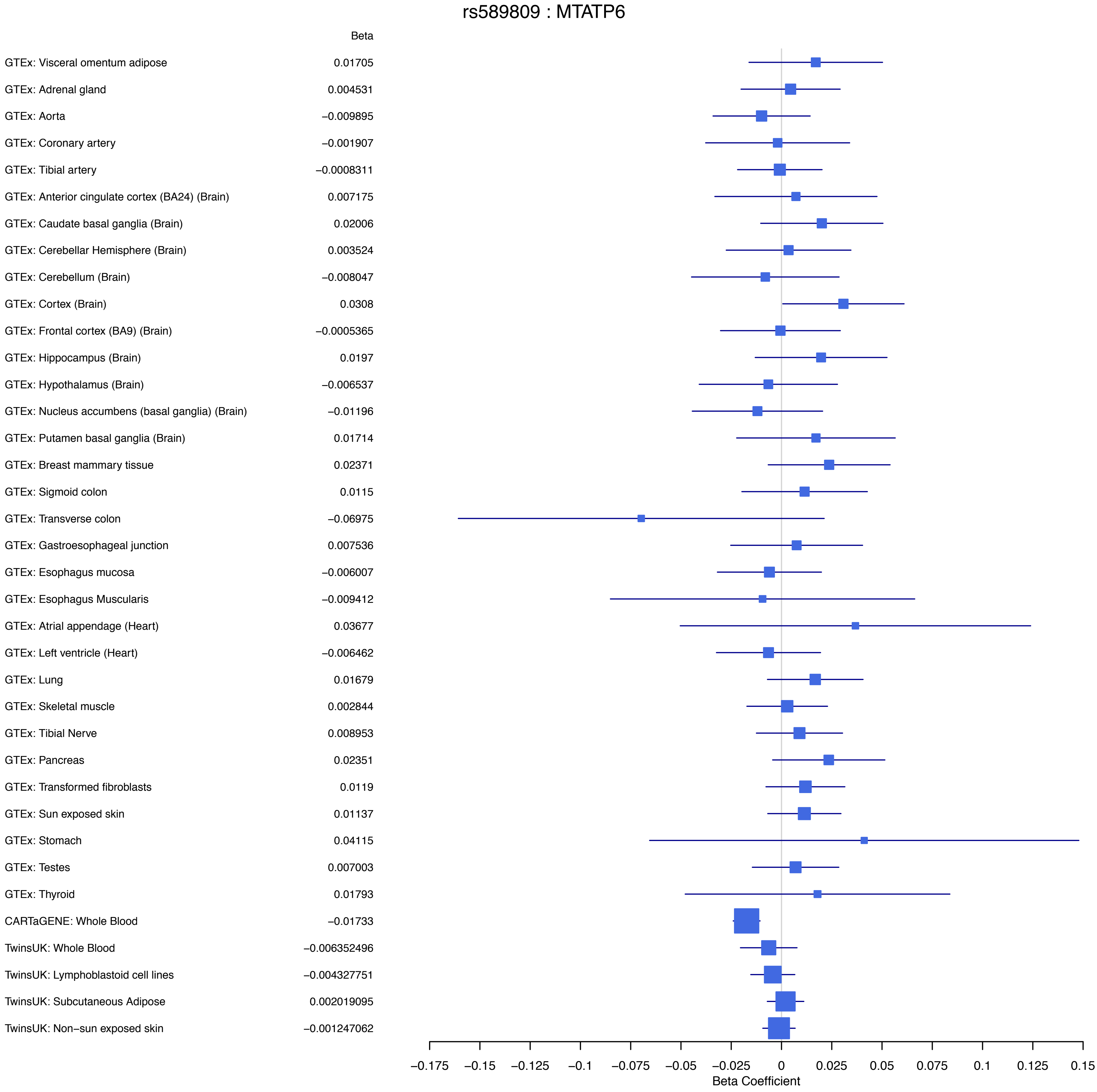
rs589809 : MTATP6
Beta
GTEx: Visceral omentum adipose 0.01705
GTEx: Adrenal gland 0.004531
GTEx: Aorta −0.009895
GTEx: Coronary artery −0.001907
GTEx: Tibial artery −0.0008311
GTEx: Anterior cingulate cortex (BA24) (Brain) 0.007175
GTEx: Caudate basal ganglia (Brain) 0.02006
GTEx: Cerebellar Hemisphere (Brain) 0.003524
GTEx: Cerebellum (Brain) −0.008047
GTEx: Cortex (Brain) 0.0308
GTEx: Frontal cortex (BA9) (Brain) −0.0005365
GTEx: Hippocampus (Brain) 0.0197
GTEx: Hypothalamus (Brain) −0.006537
GTEx: Nucleus accumbens (basal ganglia) (Brain) −0.01196
GTEx: Putamen basal ganglia (Brain) 0.01714
GTEx: Breast mammary tissue 0.02371
GTEx: Sigmoid colon 0.0115
GTEx: Transverse colon −0.06975
GTEx: Gastroesophageal junction 0.007536
GTEx: Esophagus mucosa −0.006007
GTEx: Esophagus Muscularis −0.009412
GTEx: Atrial appendage (Heart) 0.03677
GTEx: Left ventricle (Heart) −0.006462
GTEx: Lung 0.01679
GTEx: Skeletal muscle 0.002844
GTEx: Tibial Nerve 0.008953
GTEx: Pancreas 0.02351
GTEx: Transformed fibroblasts 0.0119
GTEx: Sun exposed skin 0.01137
GTEx: Stomach 0.04115
GTEx: Testes 0.007003
GTEx: Thyroid 0.01793
CARTaGENE: Whole Blood −0.01733
TwinsUK: Whole Blood −0.006352496
TwinsUK: Lymphoblastoid cell lines −0.004327751
TwinsUK: Subcutaneous Adipose 0.002019095
TwinsUK: Non−sun exposed skin −0.001247062
−0.175 −0.15 −0.125 −0.1 −0.075 −0.05 −0.025 0 0.025 0.05 0.075 0.1 0.125 0.15
Beta Coefficient
