# Supplementary material for: Nuclear genetic regulation of the human mitochondrial transcriptome
Source: eLife. 2019 Feb 18;8:e41927. doi: 10.7554/eLife.41927 (PMC6420317; doi:10.7554/eLife.41927)
Supplement: Transparent reporting form [file elife-41927-transrepform.pdf]

eLife's transparent reporting form

We encourage authors to provide detailed information *within their submission* to facilitate the interpretation and replication of experiments. Authors can upload supporting documentation to indicate the use of appropriate reporting guidelines for health-related research (see EQUATOR Network), life science research (see the BioSharing Information Resource), or the ARRIVE guidelines for reporting work involving animal research. Where applicable, authors should refer to any relevant reporting standards documents in this form.

If you have any questions, please consult our Journal Policies and/or contact us: editorial@elifesciences.org.

Sample-size estimation

- You should state whether an appropriate sample size was computed when the study was being designed
- You should state the statistical method of sample size computation and any required assumptions
- If no explicit power analysis was used, you should describe how you decided what sample (replicate) size (number) to use

Please outline where this information can be found within the submission (e.g., sections or figure legends), or explain why this information doesn't apply to your submission:

Association analyses for the detection of expression quantitative trait loci (eQTLs) were performed using pre-existing RNA sequencing and genotyping datasets, which were designed to be sufficiently powered to detect eQTLs in the original studies.

Replicates

- You should report how often each experiment was performed
- You should include a definition of biological versus technical replication
- The data obtained should be provided and sufficient information should be provided to indicate the number of independent biological and/or technical replicates
- If you encountered any outliers, you should describe how these were handled
- Criteria for exclusion/inclusion of data should be clearly stated
- High-throughput sequence data should be uploaded before submission, with a private link for reviewers provided (these are available from both GEO and ArrayExpress)

Please outline where this information can be found within the submission (e.g., sections or figure legends), or explain why this information doesn't apply to your submission:

eLIFE
1st Floor
24 Hills Road
Cambridge CB2 1JP, UK
P 01223 855340
W elifesciences.org
T @elife

A full description of the data used is provided in the Materials and Methods, under the header ‘Data’. This section describes sample sizes and sequencing/genotyping technologies used for each data source.

Removal of samples for quality control reasons is fully described in the Materials and Methods, at the beginning of the two sections detailing how data was processed (‘Processing of RNA sequencing data’ and ‘Processing of Genotyping Data’). For dataset and tissue type eQTL analyses, we remove gene expression outliers that are three interquartile ranges above or below the upper and lower quartile respectively to avoid spurious statistical associations. This is detailed in the ‘Association Analyses’ section of the Materials and Methods. For validation of genetic associations using qPCR, we remove outliers that are three interquartile ranges above or below the upper and lower quartile respectively within each genotypic category. This is described in the ‘Validation’ section of the Materials and Methods.

Anonymized processed mitochondrial encoded gene expression matrices are available in supplementary file 4, which is noted in the ‘Association Analyses’ section of the Materials and Methods, and also in the Acknowledgements. We have also uploaded these to the Gene Expression Omnibus under accession GSE125013.

Statistical reporting

- Statistical analysis methods should be described and justified
- Raw data should be presented in figures whenever informative to do so (typically when N per group is less than 10)
- For each experiment, you should identify the statistical tests used, exact values of N, definitions of center, methods of multiple test correction, and dispersion and precision measures (e.g., mean, median, SD, SEM, confidence intervals; and, for the major substantive results, a measure of effect size (e.g., Pearson's r, Cohen's d)
- Report exact p-values wherever possible alongside the summary statistics and 95% confidence intervals. These should be reported for all key questions and not only when the p-value is less than 0.05.

Please outline where this information can be found within the submission (e.g., sections or figure legends), or explain why this information doesn't apply to your submission:

To compare gene expression values along the mitochondrial transcriptome, we detail the use of a one-way ANOVA, with accompanying P values within the text in the 'Variation in Mitochondrial Gene Expression' of the Results.

For eQTL association analyses, we detail the model and covariates used in each case, within the 'Association Analyses' section of the Materials and Methods, along with the correction method (FDR). P-values and associated Beta values for significant associations are provided in supplementary file 1 and table 2, as are the corresponding P values and betas for replication analyses. QQ plots for replicated associations are also provided as Figure 2 – figure supplement 2. The criteria for cross-tissue comparison and replication of associations (Bonferroni correction, same direction of effect) are detailed in each relevant section ('Association Analyses' and 'Replication and Validation of Associations' respectively).

For mediation analysis, we detail the criteria, methods used and P-value correction (FDR) in the 'Functional Annotation and links to Complex Disease' section of the Materials and Methods.

For validation of genetic associations using qPCR, we detail the P value, test used (one-way ANOVA) and sample sizes in the 'Replication and Validation of Associations' section of the Results and 'Validation' section of the Materials and Methods.

(For large datasets, or papers with a very large number of statistical tests, you may upload a single table file with tests, Ns, etc., with reference to sections in the manuscript.)

Group allocation

- Indicate how samples were allocated into experimental groups (in the case of clinical studies, please specify allocation to treatment method); if randomization was used, please also state if restricted randomization was applied
- Indicate if masking was used during group allocation, data collection and/or data analysis

Please outline where this information can be found within the submission (e.g., sections or figure legends), or explain why this information doesn't apply to your submission:

eLife Sciences Publications, Ltd is a limited liability non-profit non-stock corporation incorporated in the State of Delaware, USA, with company number 5030732, and is registered in the UK with company number FC030576 and branch number BR015634 at the address 1st Floor, 24 Hills Road, Cambridge CB2 1JP | August 2014

Not generally applicable – for all expression QTL association analyses, samples were grouped by genotype for comparison with gene expression levels.

Additional data files ("source data")

- We encourage you to upload relevant additional data files, such as numerical data that are represented as a graph in a figure, or as a summary table
- Where provided, these should be in the most useful format, and they can be uploaded as "Source data" files linked to a main figure or table
- Include model definition files including the full list of parameters used
- Include code used for data analysis (e.g., R, MatLab)
- Avoid stating that data files are "available upon request"

Please indicate the figures or tables for which source data files have been provided:

Anonymized processed mitochondrial encoded gene expression matrices are available in supplementary file 4, which is noted in the 'Association Analyses' section of the Materials and Methods, and also in the Acknowledgements. We have also uploaded these to the Gene Expression Omnibus under accession GSE125013.
